# Can mHealth Technology Help Mitigate the Effects of the COVID-19 Pandemic?

**DOI:** 10.1109/OJEMB.2020.3015141

**Published:** 2020-08-07

**Authors:** Catherine P. Adans-Dester, Stacy Bamberg, Francesco P. Bertacchi, Brian Caulfield, Kara Chappie, Danilo Demarchi, M. Kelley Erb, Juan Estrada, Eric E. Fabara, Michael Freni, Karl E. Friedl, Roozbeh Ghaffari, Geoffrey Gill, Mark S. Greenberg, Reed W. Hoyt, Emil Jovanov, Christoph M. Kanzler, Dina Katabi, Meredith Kernan, Colleen Kigin, Sunghoon I. Lee, Steffen Leonhardt, Nigel H. Lovell, Jose Mantilla, Thomas H. McCoy, Nell Meosky Luo, Glenn A. Miller, John Moore, Derek O'Keeffe, Jeffrey Palmer, Federico Parisi, Shyamal Patel, Jack Po, Benito L. Pugliese, Thomas Quatieri, Tauhidur Rahman, Nathan Ramasarma, John A. Rogers, Guillermo U. Ruiz-Esparza, Stefano Sapienza, Gregory Schiurring, Lee Schwamm, Hadi Shafiee, Sara Kelly Silacci, Nathaniel M Sims, Tanya Talkar, William J. Tharion, James A. Toombs, Christopher Uschnig, Gloria P. Vergara-Diaz, Paul Wacnik, May D. Wang, James Welch, Lina Williamson, Ross Zafonte, Adrian Zai, Yuan-Ting Zhang, Guillermo J. Tearney, Rushdy Ahmad, David R. Walt, Paolo Bonato

**Affiliations:** Paolo Bonato is with the Department of Physical Medicine and RehabilitationHarvard Medical School at Spaulding Rehabilitation Hospital 24498 Boston MA 02129 USA; Wyss InstituteHarvard University 1812 Cambridge MA 02138 USA

**Keywords:** COVID-19, digital contact tracing, electronic patient reported outcomes (ePRO), mHealth technology, wearable sensors

## Abstract

*Goal:* The aim of the study herein reported was to review mobile health (mHealth) technologies and explore their use to monitor and mitigate the effects of the COVID-19 pandemic. *Methods:* A Task Force was assembled by recruiting individuals with expertise in electronic Patient-Reported Outcomes (ePRO), wearable sensors, and digital contact tracing technologies. Its members collected and discussed available information and summarized it in a series of reports. *Results:* The Task Force identified technologies that could be deployed in response to the COVID-19 pandemic and would likely be suitable for future pandemics. Criteria for their evaluation were agreed upon and applied to these systems. *Conclusions:* mHealth technologies are viable options to monitor COVID-19 patients and be used to predict symptom escalation for earlier intervention. These technologies could also be utilized to monitor individuals who are presumed non-infected and enable prediction of exposure to SARS-CoV-2, thus facilitating the prioritization of diagnostic testing.

## I. Introduction

In the wake of the COVID-19 pandemic, the potential role of mobile wireless technology for public health, commonly referred to as mHealth [1], has gained the attention of the public at large. mHealth technology could be used to monitor patients with mild symptoms who have tested positive for COVID-19. These patients are typically instructed to self-quarantine at home [2] or undergo monitoring at community treatment centers [3]. However, a portion of them eventually experience an exacerbation, namely the sudden occurrence of severe symptoms, and require hospitalization. In a recent report from South Korea, approximately 2% of those initially experiencing mild symptoms, and hence treated in community centers, were eventually admitted to a hospital as they developed more severe symptoms [3]. In this context, mHealth technology could enable early detection of such exacerbations, allowing clinicians to deliver necessary interventions in a timely manner thus improving clinical outcomes [4]. Smartphone applications enabling self-reports [5], [6] and wearable sensors enabling physiological data collection [7] could be used to monitor clinical personnel and detect early signs of an outbreak in the hospital/healthcare settings [8]. Similarly, in the community, early detection of COVID-19 cases could be achieved by building upon prior studies which showed that by using wearable sensors to capture resting heart rate and sleep duration it is possible to predict influenza-like illness rates [9] as well as COVID-19 epidemic trends [10]. Furthermore, cellular phone network functionalities could provide the means to identify hotspots (e.g., crowded areas in skilled nursing facilities and food processing plants [11]). Smartphone applications for digital contact tracing could be used to monitor the population in regions at risk for an outbreak and identify as well as isolate COVID-19 cases and those who may have been exposed [12]. Finally, mHealth technology could be used to monitor COVID-19 survivors, establish phenotypes associated with the long-term sequalae of COVID-19, and deploy clinical interventions [13].

To discuss these and other potential applications of mHealth technology in the context of the COVID-19 pandemic, a Task Force was established as part of the Mass General Brigham (MGB) Center for COVID Innovation [14]. The Task Force identified several use cases and generated a series of reports on related topics. These reports are available as Sections of the Supplementary Materials of this manuscript. Specifically, Section 1 provides an overview of the clinical presentation and needs related to COVID-19. Section 2 examines the use of mHealth and other technologies in field hospitals set up to respond to the COVID-19 pandemic. Section 3 discusses the use of electronic Patient-Reported Outcomes (ePRO) to screen and monitor COVID-19 cases. Section 4 provides an overview of sensing technologies to monitor patients and frontline workers. Section 5 highlights new technologies, most of which still requires substantial development efforts, that carry great potential to help address the current and future potential pandemics. Sections 6 and 7 discuss contact tracing technologies and their application in the hospital and the community settings. Section 8 reviews the role of data integration platforms. Finally, Section 9 provides a summary of the Task Force's findings.

It is worth noting that mHealth technology could help health officials address also the broader public health impact of the pandemic (given social distancing, shelter in place, work from home, etc.) on activity, nutrition, sleep, and stress management, as well as on chronic disease management when access to traditional care is limited. The impact of these factors on the population at large should not be underestimated. mHealth solutions could help people to improve activity, nutrition, sleep, and stress management as well as chronic disease management (included mental health conditions) during these challenging times in novel ways. However, the work done by the Task Force was intentionally primarily focused on the potential use of mHealth technology to mitigate transmission of SARS-CoV-2 as well as morbidity and mortality due to COVID-19 itself.

## II. Survey of ${\mathsf{m}}$Health Technologies

The technologies examined in the Task Force reports fall primarily under three broad categories: 1) ePRO systems, 2) wearable sensors, and 3) digital contact tracing technologies.

ePRO systems are digital systems to collect Patient-Reported Outcomes [15]. In the context of the COVID-19 pandemic, these systems are used to collect self-reports of signs and symptoms that the Centers for Disease Control and Prevention (CDC) has recommended adopting to determine if a diagnostic test is needed [16]. Additionally, ePRO systems can be utilized to monitor patients with mild symptoms who have tested positive for COVID-19. Tracking symptom severity in these patients is important to detect early signs of exacerbations and indicate when to provide appropriate medical intervention before severe complications arise [4].

**Fig. 1.**
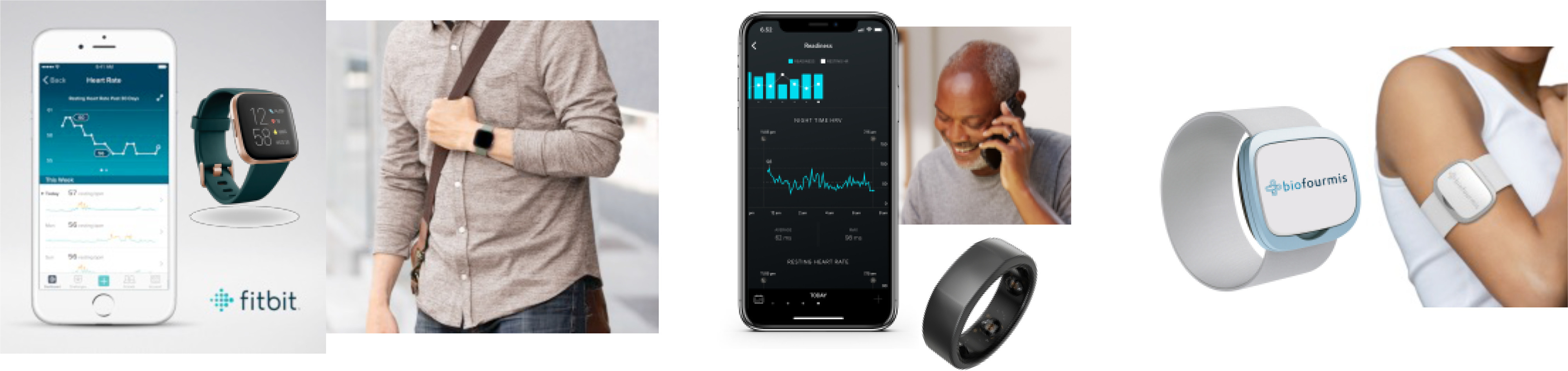
Examples of mHealth technologies: Fitbit system using the VERSA smartwatch (left panel), Oura ring sensor (panel in the middle), and the Everion system by Biofourmis (right panel). Images reproduced with permission from the manufacturers.

Wearable sensors, like the ones shown in Fig. 1, have been used to monitor physiological data and detect abnormal trends such as an excessive increase in body temperature, an increase in resting heart rate and respiratory rate, and a decrease in oxygen saturation (i.e., peripheral blood oxygen saturation; SpO2%) [17]. These types of abnormalities in physiological data have been observed in COVID-19 patients [18]. Sensor data complements ePRO data and enables the detection of subtle changes in physiological parameters that, although clinically significant, might not be perceived by patients and therefore go unreported. Monitoring individuals using wearable sensors is relevant both to detecting infection and to predicting exacerbations in patients with mild symptoms who have tested positive for COVID-19.

Digital contact tracing technologies have been designed for identifying individuals who might have been in contact with a patient who tested positive for COVID-19 [12]. For instance, smartphone applications for digital contact tracing in the community would typically exchange encrypted identifiers via Bluetooth wireless communication with smartphones of other individuals that are within the Bluetooth radio communication range. When subjects who are using the application test positive for COVID-19, individuals who have been in their proximity receive an alert and are instructed to self-quarantine and, if possible, undergo diagnostic testing.

The Task Force surveyed mHealth systems in the above-mentioned categories. Details are provided in the Supplementary Materials, not for the purpose of endorsing specific systems, but rather to present a framework to evaluate the suitability of mHealth technologies in the context of the COVID-19 pandemic. As new mHealth systems are developed and new knowledge about COVID-19 becomes available, the proposed framework and criteria for the selection of mHealth systems should be revisited and modified appropriately.

## III. Choosing the Right Technology

A significant challenge in the selection of a suitable mHealth technology in the context of the COVID-19 pandemic arises from the complexity of the disease and the fact that many of its clinical aspects are still unclear [19]. For instance, whereas the disease was originally thought of as a respiratory illness alone [20], recent findings suggest that the SARS-CoV-2, which causes COVID-19, is a vasculotropic virus [21], namely a virus that affects the blood vessels. As additional clinical data becomes available [22], new mechanisms underlying the disease are revealed. This provides the opportunity to identify symptoms and associated physiological variables suitable to detect and track disease progression.

Another challenge is the fact that COVID-19 patients may be infectious prior to being symptomatic [23]. This renders the self-report of symptoms meaningless in identifying these cases. Concerns have been raised following reports of possible asymptomatic transmission [24]. Researchers have hypothesized that, although asymptomatic, these individuals would display subtle changes in their physiology that could be detected with wearable sensors [25]. This hypothesis has been supported by observations, for instance, of low oxygen saturation in COVID-19 cases. Among others, Petrilli et al [26] analyzed data from more than 4,000 patients with COVID-19 at NYU Langone Health facilities and identified low oxygen saturation (<88%) at admission as the most important predictor of critical illness. Are low levels of oxygen saturation the result of a gradual decline that could have been detected before patients displayed clear symptoms? Results in support of this hypothesis are still limited.

Digital contact tracing technologies are not affected by the limitations associated with relying on symptom self-reports that one can collect using ePRO platforms or subtle changes in the subject's physiology detectable using wearable sensors. They are designed to identify individuals who have been in the proximity of patients who have tested positive for COVID-19 [12]. Unfortunately, it is estimated that this technology would ultimately be effective in suppressing the epidemic only if about 80% or more of smartphone users utilize it [27]. It is intuitive that, if only a small percentage of individuals use a given smartphone application for digital contact tracing, the likelihood of an outbreak being caused by a person who did not install the application is too high to make this approach viable. In addition, being within Bluetooth radio range of the smartphone of a person who tested positive for COVID-19 does not necessarily imply that a viral transmission took place. Measures of proximity and duration of contact would be relevant in this context. Moreover, in large metropolitan areas (e.g., among people using public transportation), the use of digital contact tracing technology is likely to lead to an unmanageable number of “false positives”.

## IV. mHealth Technology as a Source of Information to Prioritize Diagnostic Testing

Relying solely on data collected using mHealth technology is unlikely to be sufficient to prevent a future surge of COVID-19 cases. However, an interesting question is whether ePRO, wearable sensor, and digital contact tracing data could be aggregated and utilized as input to a probabilistic model to estimate the likelihood of infection on an individual basis and thus prioritize diagnostic testing accordingly.

**Fig. 2.**
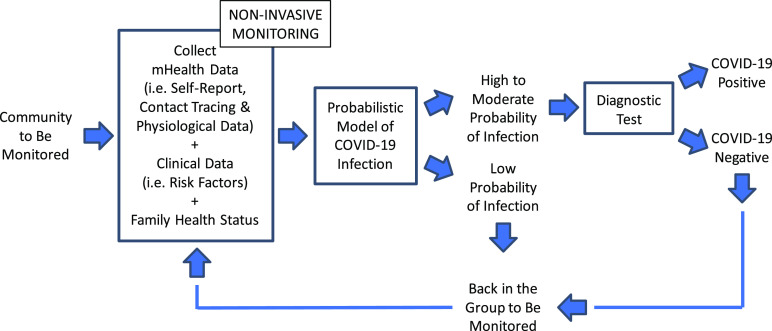
Schematic representation of a potential approach to combining mHealth technology and diagnostic testing to identify subjects who have been infected with COVID-19.

Fig. 2 shows a schematic representation of the above-described approach. In this hypothetical situation, the community of interest undergoes monitoring using ePRO, wearable sensor, and digital contact tracing technologies. In addition, individual clinical data and other factors relevant to assessing the probability of infection (e.g., the presence of co-morbid conditions or the health status of family members) are collected. This information is gathered in compliance with existing privacy laws and relevant regulations as well as attention to privacy concerns. A probabilistic model is then utilized to estimate the likelihood of infection. When the model output indicates a moderate to high probability of infection, subjects are instructed to undergo diagnostic testing.

The simplified schema shown in Fig. 2 is meant to illustrate what we believe is an important concept, namely the need for combining mHealth technology with the biology of testing. Many additional factors would have to be taken into account in any real-life deployment.

For instance, cultural and economic barriers to the adoption of mHealth technologies would need to be overcome to reach communities that are typically underrepresented in biomedical research [28], as these communities appear to be disproportionally affected by COVID-19 [29]. Privacy concerns would need to be addressed to avoid a negative impact on adoption and compliance [30], [31]. Reimbursement mechanisms would need to be identified [32], [33]. Policies to grant access to the individual data would need to be established while striking a balance between empowering patients and allowing clinicians and public health officials to deploy early interventions [34], [35]. In this context, the integration of mHealth technologies and electronic health record (EHR) systems is of paramount importance [36].

The analysis of the massive amount of data that would be collected in the context of the proposed approach is also a significant challenge. For instance, algorithms would need to be developed to identify trends and deviations from normative data in the physiological variables monitored using wearable sensors [37]. ePRO data would need to be translated into actionable items via algorithms designed to process self-reported symptoms [38], [39]. Digital contact tracing data will need to be analyzed relying on knowledge and models developed for other infectious diseases [40], [41] and, more recently, for COVID-19 [42]–[44]. Finally, this information would need to be merged and combined with epidemiological data and available data concerning risk factors affecting the individuals undergoing monitoring with the objective of generating accurate estimates of the likelihood of infection on a subject-by-subject basis. To our knowledge, models to combine all these sources of information have not been developed yet for COVID-19.

Furthermore, the approach to testing depicted in Fig. 2 is an oversimplification of the complex process of detecting infections and related information in a real-life deployment of the proposed approach. For instance, integrating available immune status tests in the schema shown in Fig. 2 would be highly desirable [45]. Similarly, procedures for random sampling of the population [46] should be implemented to minimize the likelihood that infected individuals who are asymptomatic might cause a surge in COVID-19 cases [47]. Rapid and accurate testing methodologies need to be identified. Still, testing procedures should be streamlined. mHealth technology can play an important role in this context. As it has been shown that a single positive COVID-19 sample can be detected by qRT-PCR in pools of up to 32 samples [48], [49], mHealth data could be used to predict which individuals are at low risk and whose samples could therefore be pooled together to increase testing capacity.

## V. Conclusion

mHealth technology can play an important role in monitoring individuals who could be COVID-19 positive and are instructed to self-quarantine at home, as they experience mild symptoms. During their quarantine, some of these individuals experience an exacerbation of symptoms and require hospitalization. mHealth technology could enable early detection of exacerbations and the deployment of clinical interventions before further complications arise.

When combined with diagnostic and immune status testing, mHealth technology could be a valuable tool to help mitigate, if not prevent, the next surge of COVID-19 cases. Specifically, mHealth technology could provide the means to estimate the probability of infection and prioritize diagnostic testing in individuals whose data suggests a moderate to high probability of infection. Three mHealth technologies suitable to achieve this goal were discussed in this manuscript and the Supplementary Materials section: 1) ePRO systems, 2) wearable sensors, and 3) digital contact tracing technologies. We believe that combining these technologies into an integrated, holistic mHealth solution would provide the opportunity to deploy an end-to-end solution incorporating tools for screening, risk profiling, achieving early detection, generating referrals for testing, tracking infections, tracking isolation management/quarantine, assuring social distance compliance, proving remote care, and tracking recovery.

As we witness a digital transformation of the healthcare system, mHealth technologies are expected to become better integrated in the clinical workflow. During the COVID-19 pandemic, this transformation of the healthcare system has been dramatically accelerated by new clinical demands [50] including the need to assure continuity of clinical care services. This trend is likely to make us better prepared to address the challenges of future surges of COVID-19 cases and to minimize the effects of future pandemics on routine clinical service [51], [52].

## VI. Supplementary Materials

The Supplementary Materials section of the manuscript contains the Task Force reports, as well as the affiliations and conflict of interest statements of all co-authors (pp. 65--70 of the Supplementary Materials section). The document is available in IEEE Xplore under “media”.



## VII. Disclaimer

The opinions or assertions contained in this manuscript are the private views of the authors and are not to be construed as official or as reflecting the views of the MGB network, the MGB Center for COVID Innovation, the Army, the Department of Defense, or any other Institutions the authors are affiliated with. This document was compiled using a consensus process that provided all co-authors with the opportunity to discuss and contribute to its content. Specific product depictions, illustrations or descriptions should not be considered endorsements, recommendations or specific criticisms on the part of the MGB network, its affiliated institutions, the Army, the Department of Defense, or any other Institutions the authors are affiliated with.
