# Supplementary material for: Can mHealth Technology Help Mitigate the Effects of the COVID-19 Pandemic?
Source: IEEE Open J Eng Med Biol. 2020 Aug 7;1:243–8. doi: 10.1109/OJEMB.2020.3015141 (PMC8023427; doi:10.1109/OJEMB.2020.3015141)
Supplement: Supplementary file 1 [file supp1-3015141.pdf]

Supplementary Materials

Can mHealth Technology Help Mitigate the Effects of the COVID-19 Pandemic?

Catherine P. Adans-Dester, Stacy Bamberg, Francesco P. Bertacchi, Brian Caulfield, Kara Chappie, Danilo Demarchi, M. Kelley Erb, Juan Estrada, Eric E. Fabara, Michael Freni, Karl E. Friedl, Roozbeh Ghaffari, Geoffrey Gill, Mark S. Greenberg, Reed W. Hoyt, Emil Jovanov, Christoph M. Kanzler, Dina Katabi, Meredith Kernan, Colleen Kigin, Sunghoon I. Lee, Steffen Leonhardt, Nigel H. Lovell, Jose Mantilla, Thomas H. McCoy, Jr., Nell Meosky Luo, Glenn A. Miller, John Moore, Derek O'Keeffe, Jeffrey Palmer, Federico Parisi, Shyamal Patel, Jack Po, Benito L. Pugliese, Thomas Quatieri, Tauhidur Rahman, Nathan Ramasarma, John A. Rogers, Guillermo U. Ruiz-Esparza, Stefano Sapienza, Gregory Schiurring, Lee Schwamm, Hadi Shafiee, Sara Kelly Silacci, Nathaniel M Sims, Tanya Talkar, William J. Tharion, James A. Toombs, Christopher Uschnig, Gloria P. Vergara-Diaz, Paul Wacnik, May D. Wang, James Welch, Lina Williamson, Ross Zafonte, Adrian Zai, Yuan-Ting Zhang, Guillermo J. Tearney, Rushdy Ahmad, David R. Walt, Paolo Bonato

THE sections of this document provide a summary of the findings of the Task Force on mHealth Technology that was assembled as part of the Mass General Brigham (MGB) Center for COVID Innovation. The Task Force included experts in the field of mHealth technology from MGB affiliated hospitals as well as other academic institutions and companies across the world.

The document is organized in the following nine sections.

Section Title	Page #
Section 1 – COVID-19 Related Clinical Issues, mHealth Technology Applications, and the Acceleration of the Digital Health Transformation	2
Section 2 – Monitoring Service Providers and Patients in a Disaster Scenario	10
Section 3 –ePRO Solutions to Screen and Monitor COVID-19 Cases	17
Section 4 – Remote Monitoring of Patients with COVID-19 and Frontline Healthcare Workers Using mHealth Technologies	23
Section 5 – Emerging mHealth Technologies for Monitoring and Prevention of COVID-19	32
Section 6 – Technology-assisted Contact Tracing in the Hospital Setting	40
Section 7 – Technology-Based Contact Tracing Solutions to Contain the Spread of COVID-19 in the Community	48
Section 8 – mHealth Data Integration Platforms	55
Section 9 – Summary Comments Including Issues and Opportunities	62

The affiliations of the individual Task Force members as well as their financial and non-financial disclosures are provided in a separate section at the end of the Supplementary Materials (pp. 65-70).

The opinions or assertions contained in this manuscript are the private views of the authors and are not to be construed as official or as reflecting the views of the MGB network, the MGB Center for COVID Innovation, the Army, the Department of Defense, or any other Institution the authors are affiliated with. This document was compiled using a consensus process that provided all co-authors with the opportunity to discuss and contribute to its content. Specific product depictions, illustrations or descriptions should not be considered endorsements, recommendations or specific criticisms on the part of MGB network or its affiliated institutions, the Army, the Department of Defense, or any other Institution the authors are affiliated with.

This report was completed as many clinical aspects of COVID-19 remain unclear and as mHealth technology researchers are responding to the COVID-19 crisis by generating new knowledge at an unprecedented pace. Hence, specific considerations concerning the technologies surveyed in this report will, in all likelihood, become obsolete in a relatively short period of time. However, the approach utilized by the Task Force to assess and compare mHealth technologies, in the context of the current and potential future pandemics, is expected to remain valid and to provide a framework to evaluate new mHealth systems as they become available.

COVID-19 Related Clinical Issues, mHealth Technology Applications, and the Acceleration of the Digital Health Transformation

1.1 INTRODUCTION

The world is currently confronting a major global pandemic. The alarming growth of COVID-19 cases has highlighted the shortcomings of healthcare systems, governmental policies, and exposed wider societal issues. The response to COVID-19 has shown how crucial it is to ensure appropriate preparedness and precautions, such as: social distancing, travel restrictions, availability of protective equipment, the ability to immediately implement and scale-up testing and diagnostic efforts, the capability of tracking the movements of individuals to monitor exposure, and the capability to swiftly adapt and expand the capacity of healthcare facilities [1].

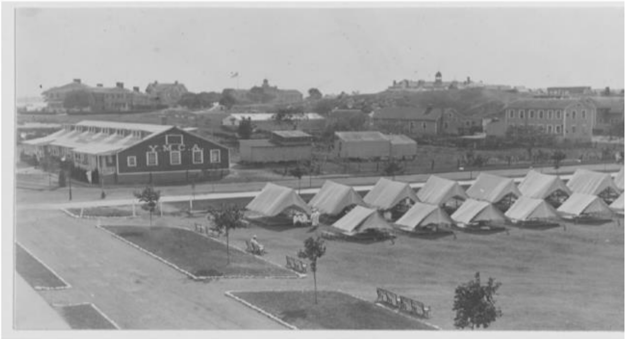

Fig. S1.1. The Lingering Sickness of War and the Pandemic of 1918-1920; *"Under the shadow of the Naval War College at the Naval Training Station. Newport, Rhode Island 1918".* Naval War College Press; monograph: Kohnen D; Jellicoe N, and Sims N. (Image available in the public domain.)

COVID-19 has brought these issues to light especially in parts of the world where other recent global viral outbreaks, such as SARS (2000-2004), H1N1 (2009), MERS (2012), and Ebola (2013-2016), had limited impact save for isolated exercises in preparedness by government agencies, certain cities, and provider organizations.

Though the field of medicine, and health-related technologies in particular, have evolved exponentially over the last century, the proposed recommendations to manage the current COVID-19 pandemic remain almost entirely unchanged from those made during the outbreak of the Pandemic of 1918-1920 [2]. These include social distancing, hand hygiene and avoiding touching the face, along with a dramatic expansion of hospital beds and field hospitals to care for the severely ill and those unable to implement social distancing (Fig. S1.1).

As such, mHealth technologies offer a new paradigm that can be leveraged to assist in managing these crises ranging from an individual level (e.g., reminders to wash your hands, alerts on hand position to avoid touching the face) to institutional responses to address other essential clinical issues such as prevention, treatment, and monitoring.

This working group has identified three areas of clinical need that span three distinct but interconnected phases of the COVID-19 pandemic:

1) *Pandemic COVID-19 clinical care* (i.e., the emergency response phase and disease detection and monitoring)
2) *Recovery or post-COVID-19 clinical care* (i.e., people in recovery in need of monitoring)
3) *Preventive care and actions* (i.e., public health measures such as handwashing, social distancing, or vaccines)

mHealth technology solutions are applicable across the span of the indicated phases of the pandemic (Fig. S1.2).

- The rapid increase in COVID-19 related inpatient admissions requires creating alternative care sites in the most affected states to allow for overflow of patients to those sites (Phase 1)
- The vast majority (~80%) of COVID-19 positive patients have mild or moderate symptoms, are quarantined at home, and need to be monitored (Deployment in Phases 1 and 2).
- The highly contagious character of the disease requires monitoring and protecting frontline healthcare-workers (Phases 1, 2 and 3).
- The rapid spread of the virus in the population at-large demands that public health officials inform, monitor and trace individuals (Phases 1, 2 and 3).
- The high incidence of COVID-19 cases in nursing homes, assisted living facilities, shelters and prisons highlight the need to more closely monitor individuals in these institutions (Phases 1, 2 and 3).
- The essential need to maintain non-COVID, non-critical clinical services for the general population, at times when access to clinical sites is limited, requires new modalities of intervention (Phases 1, 2 and 3).

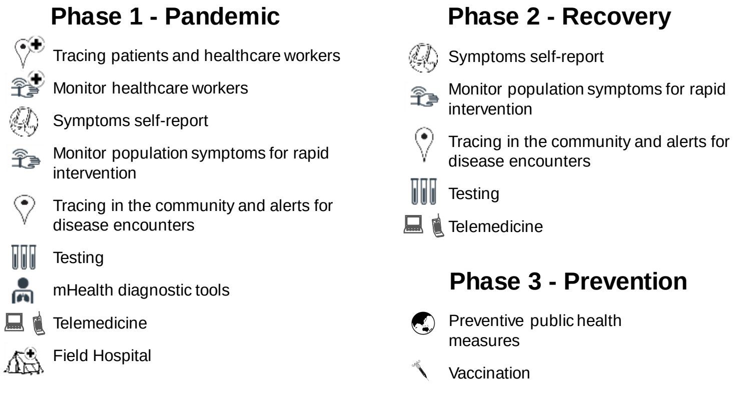

Fig. S1.2. Clinical needs and mHealth applications during COVID-19 pandemic

A crucial component of the clinical response to a crisis-situation like the COVID-19 pandemic is the sharing of knowledge and coordination of available resources within a healthcare system and the larger geographic region. This need will require unparalleled cooperation when attaining and retaining equipment, technology, facilities and frontline healthcare-workers. This section of the Supplementary Materials summarizes the clinical issues in which mHealth technologies could have a meaningful impact, if not at the current state of the health crisis, then perhaps in future deployments.

1.2 The COVID-19 Pandemic

COVID-19 is a recently described disease caused by the severe acute respiratory syndrome coronavirus 2 (SARS-CoV-2) that quickly spread across borders, and rapidly became a pandemic [3]. Of note, asymptomatic infected persons are contagious. As of June 28th 2020, the disease has already affected 10 million people and caused nearly 500,000 deaths worldwide [4]. These figures are increasing daily, and are distributed across age groups in the population [5]. While the mortality rate is notably higher in the elderly; even younger populations have seen wide hospitalizations and intensive care unit admissions. The date, the proportion of severe or fatal infections has differed significantly by geographic location [4]. The reason for this difference is still unclear, and may be influenced by demographic distributions of comorbidities and risk factors, as well as the underlying societal infrastructure that ultimately affects disease progression and fatality [6].

The transmission of SARS-CoV-2 is still being studied, but direct contact, spreading via droplets bigger than 5 microns in diameter (able to travel up to 6 feet) and fomites are considered to be the primary means of transmission during close, unprotected contact with infected individuals [3]. Airborne transmission and other possible means of viral spread need further investigation as the virus has been found viable in aerosols for up to 3 hours and stable on various surfaces between 4 to 72 hours [7].

The clinical presentation of COVID-19 is not specific and cannot reliably be distinguished from other viral infections [3]. The description of the symptomatology has evolved during the course of the pandemic, and new symptoms and complications have been reported since the first description of the disease was published (Table S1.1). The disease’s clinical presentation can range from being asymptomatic or presenting with very mild symptoms, to severe illness or even death of affected patients [3]. Current findings show that the symptoms may develop five days to two weeks after exposure to the virus [3], [8] and the presentation can vary throughout the duration of the disease. Recent literature has indicated that the most common symptoms are fever, dry cough, fatigue, sputum production, and shortness of breath [3], [9], [10]. But also other non-specific symptoms such as sore throat, myalgia or arthralgia, chills, headache, gastrointestinal issues, nasal congestion, hemoptysis, and conjunctival congestion have been observed [3], [9], [10]. Anosmia and ageusia have also recently been reported as early symptoms of the COVID-19 disease [11]. No typical skin reaction was initially ascribed to COVID-19, but, as the number of cases have increased, some characteristic patterns have been reported [12]. Additionally, the viral infection may either induce new cardiac pathologies (e.g., acute coronary syndromes, myocardial injuries, arrythmias) or exacerbate underlying cardiovascular diseases, including heart failure [13]. Cardiovascular comorbidities are common in patients with COVID-19 and such patients are at a higher risk of morbidity and mortality [14]. Neurological effects have been reported to occur in COVID-19 patients, such as headache, dizziness, prominent agitation and confusion, impairment of consciousness, ataxia, epileptic manifestations, corticospinal tract signs, stroke and symptoms of peripheral nerve involvement [15], [16]. Anecdotal data has surfaced that patients post-ICU have had significant deficits in motor planning and balance, and increased incidence of depression. There is still a need to establish which, if any, of those symptoms are specific to the SARS-CoV-2 infection [16], [17].

COVID-19 SYMPTOMS			
**Symptoms**	**Reported Frequency**	**Initially Reported**	**Recently Reported**
**Fever**	87.9 - 98%[2,7]	✔	
**Dry Cough**	67.7 – 76%[2,7,8]	✔	
**Fatigue**	38.1%[2]	✔	
**Sputum Production**	28 - 33.4%[2,7]	✔	
**Shorten of Breath**	18.6[2] - 55[7]%[2,7]	✔	
**Myalgia or Arthralgia**	14.8%[2]	✔	
**Sore Throat**	13.9%[2]	✔	
**Chills**	11.4%[2]	✔	
**Headache**	8 - 13.6%[2]	✔	
**Nausea or vomiting**	5%[2,8]	✔	
**Diarrhea**	3-3.8%[2,7,8]	✔	
**Nasal Congestion**	4.8%[2]	✔	
**Hemoptysis**	0.9 - 5%%[2,8]	✔	
**Conjunctival congestion**	0.8%[2]	✔	
**Malaise**	To be determined		✔
**Anosmia**	To be determined		✔
**Ageusia**	To be determined		✔
**Erythematous rash**	To be determined		✔
**Widespread urticaria**	To be determined		✔

Table S1.1. COVID-19 Clinical Presentation

Approximately 80% of COVID-19 positive individuals are asymptomatic or do not require hospitalization at the onset of the disease [3]. Despite this, the population should be quarantined and monitored at home due to the risk of infecting others as well as sudden exacerbation of symptoms that may require admission into an intensive care unit (ICU). ICU admission occurs in 5 to 8% of the overall affected population [18]. Based on preliminary data, the World Health Organization (WHO) estimates the average time period from onset to the development of severe disease to be about one week [3]. The most frequent complications are Acute Respiratory Distress

Syndrome (ARDS), sepsis, and cardiovascular and thromboembolic events [18]. The exacerbations observed in some patients evolve rapidly which may complicate monitoring outside acute hospital settings because of the need to gather frequent longitudinal measures. Therefore, it is necessary to provide clear information to patients on what might be ¨Red Flag¨ symptoms, such as difficulty breathing, chest pain, signs of hypoxia or shock (confusion, dizziness, reduced urine output, hypotension, cyanosis, and tachycardia) and to find a means of monitoring them via self-report or more objective physiological measures [19].

Criteria have been established for patients’ admission to the different healthcare settings. Admission criteria for higher-level of care units are based on abnormal sustained resting measures of physiological variables such as respiratory rate (≥ 30/min), heart rate (>120 bpm), blood oxygen saturation (≤93%), partial pressure of arterial oxygen to fraction of inspired oxygen ratio (≤300 mmHg), lung imaging (progression >50% within 24–48 hours), and the identification or development of severe chronic diseases such as hypertension, diabetes, coronary heart disease, cancer, structural lung disease, pulmonary heart disease, or immunosuppression [18], [20]. In addition, field hospitals have been set up during the pandemic often to absorb the influx of patients not critically ill as defined above, but not able to be discharged home or to another facility.

1.3 Clinical Needs In Alternative Care Sites

Surges in hospital admissions are anticipated, whether due to COVID-19 or future pandemics. These surges will require additional beds for inpatient stays, increased critical care capacity and large quantities of associated supplies. These last weeks have seen the emergence of alternative healthcare sites (or field hospitals, Fig. S1.3) created to absorb the overflow of patients either infected with COVID-19 or otherwise requiring hospitalization. Readers are referred to Section 2 (“Monitoring Service Providers and Patients in a Disaster Scenario”) for additional information. At these sites, patients are monitored by healthcare professionals, but they are not in need of ICU-level hospitalization. Most alternative care sites are repurposed facilities, with their own logistic challenges. Preparation for future healthcare crises can leverage the knowledge gained from the current pandemic to develop more sophisticated plans for deploying field hospitals.

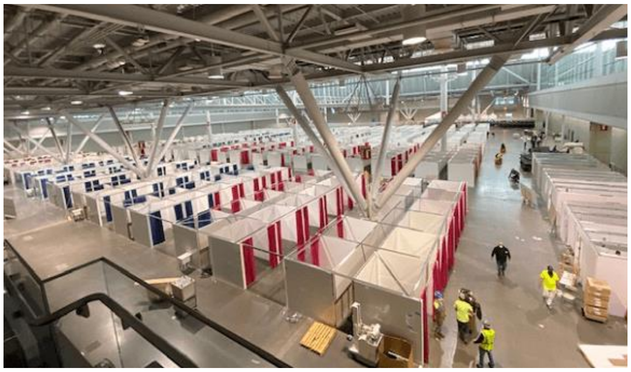

Fig. S1.3. Field hospital in the Boston Convention and Exhibition Center: the Boston Hope Center. (Image reproduced with permission from Massachusetts General Hospital.)

A major challenge in creating these emergency clinical spaces is often the unavailability of a physiological monitoring system which can be deployed quickly and for a large number of patients. Essential parameters to monitor, such as body temperature, blood pressure, oxygen saturation and heart rate, can be captured with wearable sensors that are readily available for deployment at a large scale. In field hospitals, ideally, only sensors that have been clinically validated should be used. Patient-Reported Outcome gathering could be accomplished with questionnaires sent directly to patients via a mHealth smart device application.

Given the demands of this environment, candidate mHealth solutions must have real-time, automated data extraction capabilities and data display tools to clearly present clinical information to the care team. For instance, the automated identification of anomalies or sudden changes in physiological measures can aid in informing rapid and effective medical intervention. There will also be a need for a centralized alert platform if the data is gathered from multiple sources.

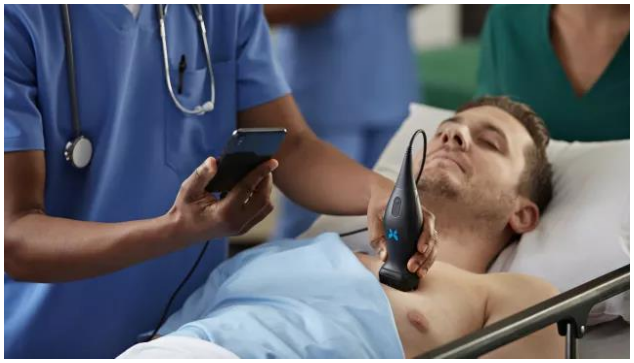

Fig. S1.4. Example of sophisticated technologies to enhance the diagnosis: a hand-held ultrasound. (Image reproduced with permission from Butterfly Network)

The deployment of mHealth technology in a field hospital will require a combination of 1) remote monitoring of physiological parameters with a centralized alert system capable of processing large amounts of data, 2) telehealth visits with off-site clinical specialists, 3) sophisticated technologies to enhance diagnoses (e.g. hand-held ultrasound [21], Fig. S1.4), while limiting unnecessary exposure of healthcare workers, and 4) sophisticated asset-tracking, patient-tracking, and personnel-tracking solutions to allow rapid evaluation and location of persons who trigger physiological monitoring alert thresholds.

1.4 Patient Home-Monitoring

The literature on and the use of mobile technologies and wearable physiological monitoring has significantly expanded in scope over the last two decades. In the context of the on-going COVID-19 pandemic, these technologies have become more relevant than ever due to the advantages they provide.

mHealth platforms are powerful tools that could aid a healthcare system straining to meet demand. If deployed, these technologies could be used by healthcare professionals to monitor patients’ conditions remotely and continuously. Additionally, such platforms, with appropriate information technology (IT) and health literacy, can empower patients to

better manage their condition themselves. Tools like electronic Patient-Reported Outcomes (ePROs) and telehealth could be implemented to stratify and triage patients, mitigating or preventing hospital surges. Readers are referred to Section 3 (“ePRO Solutions to Screen and Monitor COVID-19 Cases”) for additional information. The expansion of telehealth to the non-COVID population, in particular, will allow providers to continue caring for their non-critical patients while unburdening acute care hospitals in the wake of a viral outbreak-induced surge. It should be recognized that not every solution is appropriate for a given situation and that mHealth technologies must be properly tuned and validated before they are deployed in the field.

In China, it has been shown that closely monitoring symptoms of COVID-19 positive patients for signs of deterioration was an effective means of decreasing mortality rates [22]. This observation serves to highlight the advantage of regularly checking patients’ symptoms and acting according to each patient’s individual immediate needs. Unfortunately, the vast majority of COVID-19 positive patients, as well as people possibly exposed, are not hospitalized and the means to reliably monitor patients at home is not widely available.

For persons outside the hospital, the integration of mHealth technologies into our healthcare system can optimize patient care by enabling professionals to conveniently collect (Table S1.2) and use large amounts of patient data in their clinical practice during this pandemic and beyond. mHealth data could track people who have been exposed, monitor those infected for escalation of the disease, and assess those in recovery. These data integration platforms can inform healthcare professionals in making optimal clinical decisions based on extensive, objective, and reliable metrics, and allow them to determine monitoring status, to request a home visit, or to make a hospital referral [23]. Readers are referred to Section 8 (“mHealth Data Integration Platforms”) for additional details.

Self Reported Symptoms
General status (usual/weak)
Fever, chills (present/absent)
Shortness of breath, dyspnea (VAS 0-10)
Cough (mild/mod./severe; dry/productive)
Appetite (usual/perturbed/absent)
Smell (usual/perturbed/absent)
Taste (usual/perturbed/absent)
Sleep (usual/perturbed/somnolence)
Bladder activity (<12h)
Bowel activity (#; consistency)

Physiological Measures
Cardiac: HR (BPM), ECG
Body Temperature (Degree)
Respiratory Rate: RR (resp/min)
Oxygen Saturation - SpO2 (%)
Blood Pressure (mmHg)
Cough
Sleep (hours, quality, REM/non-REM)
Physical Activity (Activity level)
Stress (Galvanic skin response, HR variability)
Weight: Kg/lbs

Table S1.2. Symptoms and physiological measures that can be collected by mHealth technologies.

While not currently routinely deployed by healthcare providers, there do exist technologies that allow one to gather objective physiological measures of pulmonary function, such as tidal volume and lung perfusion, that would be useful in the context of preventing or mitigating the effects of COVID-19. Such technologies could be used to better understand the effects of the virus and to monitor for early signs and symptoms. For example, the nation of Liechtenstein is deploying nighttime biometric wristbands (Fig. S1.5) to monitor 5% of its population and explore whether such devices can be used as a means of early detection of outbreaks. If successful, Liechtenstein intends to expand the program to the entire population [24].

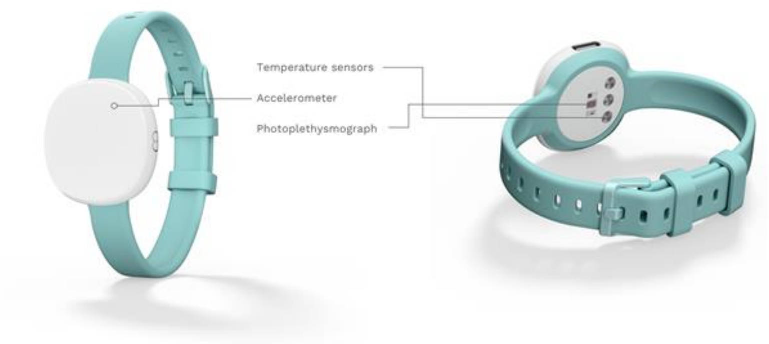

Fig. S1.5. Biometric wristband (Image reproduced with permission from Ava Science)

Monitoring at-risk populations could be essential for guiding hospital admission or readmission, due to comorbidities (e.g., hypertension, diabetes, severe obesity, severe heart disease, chronic lung disease or moderate to severe asthma, chronic kidney disease undergoing dialysis, and liver disease) or other risk factors associated with worse COVID-19 prognosis (e.g., being over the age of 65, immunocompromised, and/or institutionalized) [20], [25]. A recent report demonstrated that resting heart rate and sleep data collected with wearable sensors correlate with seasonal respiratory infection trends in a population [26]. Readers are referred to Sections 4 (“Remote Monitoring of Patients with COVID-19 and Frontline Healthcare Workers Using mHealth Technologies”) and 5 (“Emerging mHealth Technologies for Monitoring and Prevention of COVID-19”) for further details.

1.5 FRONTLINE HEALTHCARE WORKER MONITORING

As information on healthcare workers’ exposure to the virus has become more widely available, up to 3.8% of positive cases of COVID-19 have been accounted for by healthcare workers [27]. A retrospective report from China identified that approximately 40% of the COVID-19 cases were presumed to have been infected in a hospital, either via infected healthcare workers or other asymptomatic patients. This report noted that 77% of healthcare workers were infected while working on general wards, 17% in the emergency department, and only 5% in the intensive care unit [28]. High transmission rates of SARS-CoV-2 have been reported to occur in healthcare

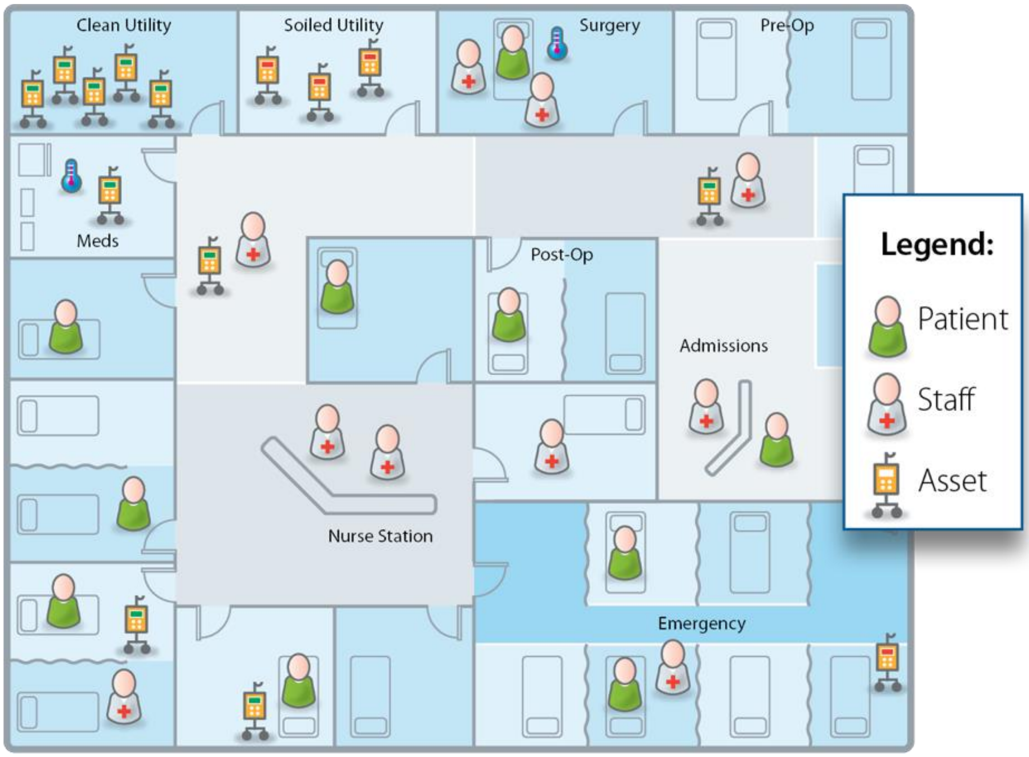

Fig. S1.6. Hospital real time location system (Image reproduced with permission from Centrak®)

workers caring for patients requiring ventilatory assistance [29], especially when adequate personal protective equipment (PPE) was not available.

In confronting COVID-19, the prevention of infectious disease transmission from patients and the protection of frontline healthcare workers is a priority [30]. There is a notable decrease in viral transmission when appropriate hygiene standards and PPE (gloves, N95 respirator and other face masks, gowns) is available and used [31]. The presence of asymptomatic COVID-19-positive cases [3], [8] complicates the standard precautions and begets an urgent need to explore 1) using technologies to detect subtle symptoms [32] (e.g., asymptomatic hypoxic events, elevated resting heart rate, or body temperature variations), that might indicate exposure, and 2) tracing hospital workers to retrospectively identify encounters with averred COVID-19 patients.

In implementing a sophisticated system to monitor the well-being of clinical staff, it may be beneficial to also track the stress levels of workers to mitigate "burnout". Such a system could provide reassurances to healthcare workers who may rightfully be concerned with being unknowingly exposed to the virus and placing their own families at risk. If all frontline caregivers were equipped with badges that track their location using a real-time location system (RTLS) with privacy protections (Fig. S1.6), then after an unexpected viral exposure, relevant location data could be retrieved to inform workers of possible viral contact. These workers could then be traced, contacted, and tested to try to reduce further spread. Readers are referred to Sections 4 ("Remote Monitoring of Patients with COVID-19 and Frontline Healthcare Workers Using mHealth Technologies") and 6 ("Technology-Assisted Contact Tracing in the Hospital Setting") for further details.

Frontline healthcare workers represent a unique opportunity to gather data longitudinally, including self-reported symptoms and physiological measures assessed with unobtrusive wearable sensors. This population will be frequently tested for exposure and immunity to the virus, which will enable retrospective analyses to identify physiological markers of early infection. The results could be applied prospectively in the event of a second wave of the COVID-19 outbreak or in future pandemics. However, the vast majority of healthcare systems are not equipped with the adequate infrastructure to trace their personnel and track physiological measures continuously. The integration of such systems into clinical practice may be critical in preparation for future public health crises. Hospitals that already have RTLS systems deployed for asset-tracking should consider extending and upgrading these systems to permit personnel tracking. Institutions that are constructing new facilities or extending their capability into field hospitals should consider all available options for personnel tracking, with the appropriate technology for each use case. For more information regarding mHealth and disaster response, readers are suggested to view Section 2 ("Monitoring Service Providers and Patients in a Disaster Scenario").

1.6 General Population Monitoring

Public health measures, such as self-isolation, the quarantining of infected patients and early detection of the disease, are critical for containing and monitoring infectious diseases such as COVID-19 [33]. To that end, "big data", when applied to addressing public health crises, has the potential to improve disease surveillance while monitoring for decline and adverse events in a target population. Improved surveillance

could lead to better-informed means of tracking disease transmission [34].

In the on-going COVID-19 crisis, researchers have identified a need for a combination of 1) community tracing, 2) disease-specific symptom tracking via self-reports, 3) telehealth consultations, and 4) physiological monitoring using wearable sensors to mitigate, if not prevent, COVID-19 outbreaks.

1) Community tracing has the goal to reduce the spread of SARS-CoV-2 while allowing for a return to a degree of normalcy. Digital contact tracing methods can be implemented via wearable and connected devices to collect information relevant to identifying possible COVID-19 exposures. The establishment of a robust community-tracing program should be considered due to the prevalence of asymptomatic carriers in the larger population [8]. Readers are referred to Section 7 (“Technology-Based Contact Tracing Solutions to Contain the Spread of COVID-19 in the Community”) for additional information about mHealth solutions for community-tracing including the discussion of design approaches meant to address potential privacy concerns.
2) Asynchronous self-reporting platforms rely on a population to voluntarily sign up to participate in a global surveillance program. In Europe, such a concept has been in use for a few years (Influenzanet) in an effort to strengthen established Sentinel physician-based systems [35]. In the United States and Canada, a volunteer disease surveillance system (Flu Near You) was created in 2011 to provide weekly reports of the occurrence of Influenza-like Illnesses (ILI) [36]. In Australia, the Department of Health monitors influenza activity and severity in the community through the National Influenza Surveillance Committee, which publishes the Influenza Syndromic Surveillance report on a weekly basis between April and October, and less frequently during the warmer months [37].
3) Telehealth should be leveraged to reduce unnecessary exposure between patients and healthcare-workers, and to triage patients to determine the degree of care they require [38].
4) Wearable sensors such as wrist-worn devices are already widely used by individuals to track their health and activity levels. Wearable sensing technologies have rapidly evolved to relatively inexpensive and unobtrusive consumer products that monitor physiological parameters (i.e., heart rate, sleep stage and oxygenation level). A recent report examined heart rate and sleep data from Fitbit users compared to influenza-like illness outbreaks; the findings suggest that wearable sensors could improve real-time monitoring and geographic surveillance of infectious disease outbreaks [26]. As previously described, there are nations already taking steps in that direction [24].

We acknowledge the need to protect personal information and that this must be considered when looking at mHealth solutions. Others have raised technical, practical and even ethical questions that highlight the challenges of implementing such solutions to monitor the spread of infectious diseases [39]. In the meantime, the collection of such data during public health emergencies could help generate projections of the pandemic’s impact.

1.7 Maintaining Non-Covid Related, Non-Critical Clinical Services

While the world’s immediate attention is focused on facing the COVID-19 pandemic, most hospitals and healthcare systems have suspended routine visits and elective surgeries to prevent hospital outbreaks as well as free up beds and equipment for COVID-19 patients.

Beyond the pandemic, other patients suffering from acute non-life-threatening illnesses or chronic conditions still have healthcare concerns to manage. And in light of the system-wide suspensions, they are relying on phone and other technologies to contact their healthcare providers to seek care.

More than 50 U.S. health systems are already using telehealth visits to allow clinicians to see patients who are at home [40]. Previously, payment for such care has sometimes been an issue, but policies were rapidly adjusted to allow increased reimbursement during public health emergencies. This may be the beginning of a paradigm shift towards an era of further expansion into digital health, in which in-clinic visits are no longer the default method of interaction between patients and clinical personnel. The regulatory issues and reimbursement restrictions that have now been relaxed will need to be the new norm in order for this new standard to take root.

To improve the delivery of care, mHealth technologies such as sensors, diagnostic kits or self-reported symptom questionnaires can be deployed directly to patients to limit physical interaction. Clinicians can rely on the technology to gather objective physiological measures from their patients, deliver interventions, track compliance, and update treatments. Of note, the advent of the Centers for Medicare and Medicaid Services (CMS) reimbursement for remote patient monitoring during the current pandemic [41], may allow health systems to develop, implement, and refine such practices and technologies for a variety of acute and health conditions over the next several years, as part of routine care techniques. These technologies could be stockpiled and rapidly deployed to the community in the event of a new pandemic or other disaster.

1.8 Conclusions

The lessons learned during this COVID-19 pandemic may shape the future of healthcare systems around the world. We believe that the integration of mHealth technologies will be a cornerstone of this new era of clinical practice.

There will be complications and hurdles to overcome. Clinicians will need to be familiarized with telehealth visit tools and learn how to leverage the power of wearable sensors to monitor their patients. Healthcare systems will need to be equipped with the appropriate monitoring technology for their workers and have the means of collecting and managing the output of automated physiological measurement systems to monitor both patients and staff.

Our Task Force is making recommendations to identify key mHealth technologies that healthcare systems should consider investigating and adopting as part of their clinical routine for both inpatient and outpatient care. By investing into mHealth,

healthcare systems will ideally lay the groundwork for an adaptable response to the next wide-ranging emergency. Future deployments of mHealth technologies during public health emergencies would be as smooth as possible and support hospitals and institutions in providing care at times of need.

REFERENCES

[1] P. Shetty, “Preparation for a Pandemic: Influenza A H1N1,” *Lancet Infect. Dis.*, vol. 9, no. 6, pp. 339–340, Jun. 2009
[2] G. A. Soper, “The Lessons of the Pandemic,” *Science*, vol. 49, no. 1274, pp. 501–506, May 1919
[3] B. Aylward and W. Liang, “Report of the WHO-China Joint Mission on Coronavirus Disease 2019 (COVID-19),” 2020. [Online]. Available: https://www.who.int/docs/default-source/coronaviruse/who-china-joint-mission-on-covid-19-final-report.pdf. [Accessed: 28-June-2020]
[4] Center for Systems Science and Engineering at Johns Hopkins University, “COVID-19 Dashboard.” Available: https://coronavirus.jhu.edu/map.html. [Accessed: 28-June-2020]
[5] Massachusetts Department of Public Health, “Coronavirus Disease 2019 (COVID-19) Cases in MA,” 2020. Available: https://www.mass.gov/doc/covid-19-cases-in-massachusetts-as-of-april-17-2020/download. [Accessed: 28-June-2020]
[6] M. A. Khafaie and F. Rahim, “Cross-Country Comparison of Case Fatality Rates of COVID-19/SARS-COV-2,” *Osong Public Heal. Res. Perspect.*, vol. 11, no. 2, pp. 74–80, Apr. 2020
[7] N. van Doremalen *et al.*, “Aerosol and Surface Stability of SARS-CoV-2 as Compared with SARS-CoV-1,” *N. Engl. J. Med.*, vol. 382, no. 16, pp. 1564–1567, Apr. 2020
[8] P. An, P. Song, Y. Wang, and B. Liu, “Asymptomatic Patients with Novel Coronavirus Disease (COVID-19),” *Balkan Med. J.*, no. April, pp. 19–20, Apr. 2020
[9] C. Huang *et al.*, “Clinical Features of Patients Infected with 2019 Novel Coronavirus in Wuhan, China,” *Lancet*, vol. 395, no. 10223, pp. 497–506, Feb. 2020
[10] W. Guan *et al.*, “Clinical Characteristics of Coronavirus Disease 2019 in China,” *N. Engl. J. Med.*, vol. 382, no. (18), pp. 1708-1720, April 2020
[11] L. A. Vaira, G. Salzano, G. Deiana, and G. De Riu, “Anosmia and Ageusia: Common Findings in COVID-19 Patients,” *Laryngoscope*, Apr. 2020 [ePUB]
[12] S. Recalcati, “Cutaneous Manifestations in COVID-19: A First Perspective,” *J. Eur. Acad. Dermatology Venereol.*, vol. 34, no. 5, pp. e212-e213, May 2020
[13] M. Madjid, P. Safavi-Naeini, S. D. Solomon, and O. Vardeny, “Potential Effects of Coronaviruses on the Cardiovascular System,” *JAMA Cardiol.*, Mar. 2020 [ePUB]
[14] K. J. Clerkin *et al.*, “Coronavirus Disease 2019 (COVID-19) and Cardiovascular Disease,” *Circulation*, vol. 141, no. 20, pp1648-1655, May 2020
[15] L. Mao *et al.*, “Neurologic Manifestations of Hospitalized Patients With Coronavirus Disease 2019 in Wuhan, China,” *JAMA Neurol.*, Apr. 2020 [ePUB]
[16] J. Helms *et al.*, “Neurologic Features in Severe SARS-CoV-2 Infection,” *N. Engl. J. Med.*, p. Apr. 2020 [ePUB]
[17] Y. Li, W. Bai, and T. Hashikawa, “The Neuroinvasive Potential of SARS-CoV2 May Be at Least Partially Responsible for the Respiratory Failure of COVID-19 Patients,” *J. Med. Virol.*, Mar. 2020 [ePUB]
[18] G. Anesi *et al*, *Coronavirus Disease 2019 (COVID-19): Critical Care Issues*. UptoDate, 2020. [Online]. Available: https://www.uptodate.com/contents/coronavirus-disease-2019-covid-19-critical-care-and-airway-management-issues. [Accessed: 28-June-2020]
[19] T. Greenhalgh, G. C. H. Koh, and J. Car, “Covid-19: A Remote Assessment in Primary Care,” *BMJ*, vol. 368, m1182, Mar. 2020.
[20] Centers for Disease Control and Prevention, “People Who Are at Higher Risk for Severe Illness.” [Online]. Available: https://www.cdc.gov/coronavirus/2019-ncov/need-extra-precautions/people-at-higher-risk.html. [Accessed: 28-June-2020]
[21] D. Buonsenso, D. Pata, and A. Chiaretti, “COVID-19 Outbreak: Less Stethoscope, More Ultrasound,” *Lancet Respir. Med.*, Mar. 2020 [ePUB}
[22] Q. Sun, H. Qiu, M. Huang, and Y. Yang, “Lower Mortality of COVID-19 by Early Recognition and Intervention: Experience from Jiangsu Province,” *Ann. Intensive Care*, vol. 10, no. 1, p. 33, 2020
[23] E.-H. Wang, L. Zhou, S.-H. K. Chen, K. Hill, and B. Parmanto, “An mHealth Platform for Supporting Clinical Data Integration into Augmentative and Alternative Communication Service Delivery: User-Centered Design and Usability Evaluation.,” *JMIR Rehabil. Assist. Technol.*, vol. 5, no. 2, p. e14, Jul. 2018
[24] S. Jones, “Liechtenstein Rolls Out Radical Covid-19 Bracelet Programme Biometric Wristbands Will Measure Vital Signs to Detect Early Indicators of Infection,” *Financial Times*, 15-Apr-2020
[25] K. McIntosh *et al*, *Coronavirus Disease 2019 (COVID-19): Epidemiology, Virology, Clinical Features, Diagnosis, and Prevention*. UpToDate, 2020. [Online]. Available: https://www.uptodate.com/contents/coronavirus-disease-2019-covid-19-epidemiology-virology-clinical-features-diagnosis-and-prevention/print. [Accessed: 28-June-2020]
[26] J. M. Radin, N. E. Wineinger, E. J. Topol, and S. R. Steinhubl, “Harnessing Wearable Device Data to Improve State-Level Real-Time Surveillance of Influenza-Like Illness in the USA: A Population-Based Study,” *Lancet Digit. Heal.*, vol. 2, no. 2, pp. e85–e93, Feb. 2020
[27] Z. Wu and J. M. McGoogan, “Characteristics of and Important Lessons From the Coronavirus Disease 2019 (COVID-19) Outbreak in China,” *JAMA*, vol.

323, no. 13, p. 1239, Apr. 2020
[28] D. Wang *et al.*, "Clinical Characteristics of 138 Hospitalized Patients With 2019 Novel Coronavirus–Infected Pneumonia in Wuhan, China," *JAMA*, vol. 323, no. 11, p. 1061, Mar. 2020
[29] R. A. Fowler *et al.*, "Transmission of Severe Acute Respiratory Syndrome during Intubation and Mechanical Ventilation.," *Am. J. Respir. Crit. Care Med.*, vol. 169, no. 11, pp. 1198–202, Jun. 2004
[30] Centers for Disease Control and Prevention (CDC), "Centers for Disease Control and Prevention: Healthcare-associated infections." [Online]. Available: https://www.cdc.gov/hai/ [Accessed: 28-June-2020]
[31] A. Bowdle and L. S. Munoz-Price, "Preventing Infection of Patients and Healthcare Workers Should Be the New Normal in the Era of Novel Coronavirus Epidemics," *Anesthesiology*, vol. 132, pp. 1292-1295, Mar. 2020
[32] X. Li *et al.*, "Digital Health: Tracking Physiomes and Activity Using Wearable Biosensors Reveals Useful Health-Related Information," *PLOS Biol.*, vol. 15, no. 1, p. e2001402, Jan. 2017.
[33] Centers for Disease Control and Prevention, "Coronavirus Disease 2019 (COVID-19)," 2020. [Online]. Available: https://www.cdc.gov/coronavirus/2019-ncov/index.html. [Accessed: 28-June-2020]
[34] S. Bansal, G. Chowell, L. Simonsen, A. Vespignani, and C. Viboud, "Big Data for Infectious Disease Surveillance and Modeling.," *J. Infect. Dis.*, vol. 214, suppl 4, pp. S375–S379, 2016
[35] C. Guerrisi *et al.*, "Participatory Syndromic Surveillance of Influenza in Europe.," *J. Infect. Dis.*, vol. 214, suppl 4, pp. S386–S392, 2016
[36] M. S. Smolinski *et al.*, "Flu Near You: Crowdsourced Symptom Reporting Spanning 2 Influenza Seasons," *Am. J. Public Health*, vol. 105, no. 10, pp. 2124–2130, Oct. 2015
[37] Australian Influenza Surveillance Report and Activity Updates. [Online]. Available: https://www1.health.gov.au/internet/main/publishing.nsf/Content/cda-surveil-ozflu-flucurr.htm?Open=&utm_source=health.gov.au&utm_medium=redirect&utm_campaign=digital_transformation&utm_content=flureport. [Accessed: 28-June-2020]
[38] J. E. Hollander and B. G. Carr, "Virtually Perfect? Telemedicine for Covid-19," *N. Engl. J. Med.*, vol. 382, pp. 1679-1681, Mar. 2020.
[39] E. C. Lee, J. M. Asher, S. Goldlust, J. D. Kraemer, A. B. Lawson, and S. Bansal, "Mind the Scales: Harnessing Spatial Big Data for Infectious Disease Surveillance and Inference.," *J. Infect. Dis.*, vol. 214, no. suppl_4, pp. S409–S413, 2016
[40] S. Duffy and T. H. Lee, "In-Person Health Care as Option B," *N. Engl. J. Med.*, vol. 378, no. 2, pp. 104–106, Jan. 2018
[41] "Covered Telehealth Services for PHE for the COVID-19 pandemic," *Center for Medicare and Medicaid Services*, 2020. [Online]. Available: https://www.cms.gov/Medicare/Medicare-General-Information/Telehealth/Telehealth-Codes. [Accessed: 28-June-2020]

Monitoring Service Providers and Patients in a Disaster Scenario

2.1 Introduction

As of June 28th 2020, the COVID-19 pandemic has infected nearly 10 million people worldwide [1] stretching many healthcare systems to their limit when rising to meet the exponentially increasing demand for hospitalizations and intensive care. Around the world, in order to expand hospital capacity, large facilities, such as gymnasiums, hotels, convention centers, and arenas have been converted into field hospitals (Fig. S2.1). These field hospitals relieve the stress on the healthcare system in two different ways: by accommodating patients with mild to moderate symptoms and by isolating healthy individuals who are particularly at risk. Even with these locations in place, critical care cases commonly remain assigned to conventional hospitals. While the purpose of field hospitals is well defined, the actual physical places they occupy can vary greatly, ranging from triage tents to converted convention centers.

Field hospital facilities have three key characteristics in common: rapid construction, massive scale, and low cost. By necessity, they must be built in a few days, accommodate large numbers of patients, and be able to be dismantled quickly once the emergency is over. Their core functions, the treatment they provide directly to patients and the type of needed support they provide to healthcare systems, are deeply connected with the features of the emergencies they were deployed to address. In this discussion, we focus on the COVID-19 pandemic, generated by a highly contagious novel coronavirus that causes, in the great majority of people, only mild to moderate symptoms [2] (see Section 1 “COVID-19 Related Clinical Issues, mHealth Technology Applications, and the Acceleration of the Digital Health Transformation” for additional clinical information).

Field hospitals provide a means of isolating infected individuals, thereby mitigating the spread of the infection seen within families when COVID-19-infected individuals are homebound [3]. In addition, field hospitals provide the patients with basic medical care, frequent monitoring, and rapid referral in case of worsening symptoms. Furthermore, as Chen et al. [3] noted in their description of the Chinese shelter hospitals built for the 2020 pandemic, these facilities have the additional benefit of relieving some of the stress patients experience by alleviating their concerns about acting as vectors for the disease, and by providing living arrangements in which limited social interactions are possible.

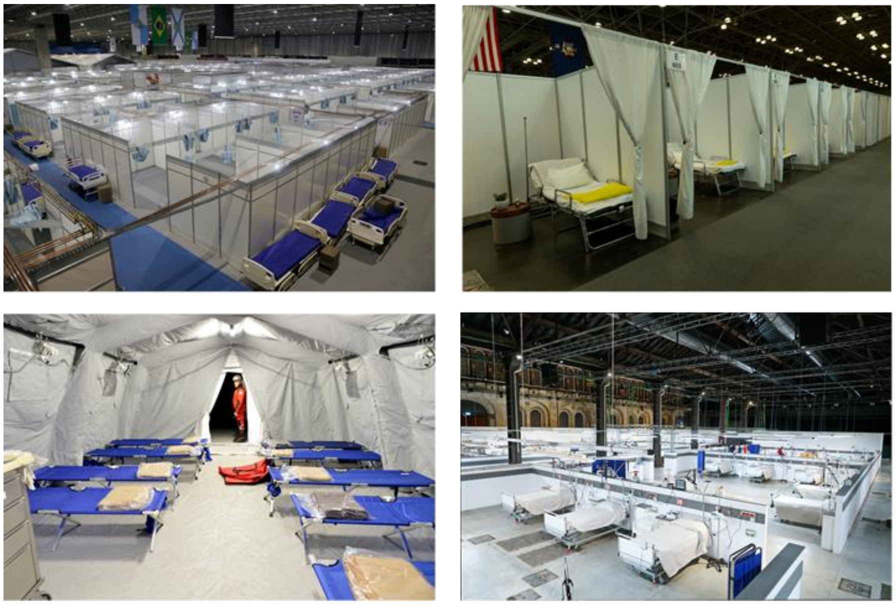

Fig. S2.1. From Top Left Clockwise: Riocentro exhibition and convention center, Rio de Janeiro, Brazil; Jacob Javits Convention Center New York City, Est Meadow in Central Park New York NY; Triage Tent in Bressanone Hospital, Italy; former industrial plant, Turin, Italy. (Images from Shutterstock.)

This section of the Supplementary Materials presents a preliminary analysis of the role mHealth technologies could play in a disaster-driven field hospital scenario. In Section 2.2, we review COVID-19 field hospital design considerations, drawing liberally from the Chinese experience; Section 2.3 describes examples of potential use of mHealth technologies; Section 2.4 describes the potential value of mHealth technologies in a COVID-19 field hospital scenario; Section 2.5 provides examples of portable medical equipment that could play important roles in field hospitals; Section 2.6 provides concluding remarks.

2.2 Field Hospitals: The Chinese Experience

A detailed description of the physical and operational organization of the “shelter hospitals” China constructed to address the COVID-19 outbreak in Wuhan can be found in [3], [4]. Wuhan, the capital of the Hubei province in China, was the epicenter of the pandemic. There, three shelter hospitals were opened within a 29 hour period by converting local exhibition centers and stadiums. These shelter hospitals played a vital triage role, providing accomodations for infected individuals with mild and moderate symptoms thus relieving some of the pressure from conventional hospitals. To ensure appropriate care, mobile medical imaging and laboratory assets were positioned adjacent to the shelter hospitals to provide on-site advanced diagnostic and imaging services.

Symptomatic individuals arriving at these field facilities were quickly triaged, with admission based on a positive COVID-19 nucleic acid test, age (<65 y), absence of serious chronic diseases (e.g., hypertension, diabetes, coronary heart disease), etc. Separate clean, semi-clean and contaminated zones were established, with designated passages for healthcare workers and patients. The contaminated isolation area was also divided into two sections: one for infected patients and the other for patients scheduled for discharge. Housed COVID-19 patients were allowed to have social interactions in dedicated areas of these temporary hospitals, reducing the mental and emotional stressors associated with isolation and infection.

Individuals apparently recovered from the disease were monitored for at least three days before being discharged. The criteria for discharge included: normal body temperature for more than three days, significant improvement of respiratory symptoms, lung imaging showing a reduction of inflammation, and negative COVID-19 tests on two consecutive occasions (with a sampling interval of at least one day). Individuals showing chronic obstructive pulmonary disease (COPD) exacerbation, or experiencing severe COVID-19 symptoms, were immediately transferred to conventional hospital facilities.

The three shelter hospitals deployed in Wuhan, totaling about 12,000 beds, were critical in China's response to the pandemic as approximately 9,000 patients were directed to these facilities. On March 10, 2020, the last patient was cleared, and the shelter hospitals were dismantled.

2.3 mHealth in COVID-19 Field Hospitals

In a scenario such as the one described above, mHealth technologies can assume a vital role in helping frontline healthcare workers successfully combat the pandemic. The first, and immediate, benefit is the continuous and real-time monitoring of patients' vital signs. This not only allows a higher granularity when monitoring the status of affected individuals inside a facility but, as importantly, provides the multi-factorial data needed for advanced analytics.

The use of rigorously trained and validated mathematical models may improve specificity and reduce the likelihood of false-positive or false-negative alerts regarding an individual's condition. The possibility of streaming data to nursing stations, in combination with automatic alerts, could significantly reduce the need for interactions between patients and staff. In turn, this could improve the coverage of a large number of patients with limited personnel and potentially improve the efficient deployment of clinical staff.

Monitoring is not only limited to bio-signals, but could be extended to tracing and real-time tracking of patient and personnel locations within a facility via indoor positioning systems (IPS) or real-time location systems (RTLS). These technologies are particularly important when healthy non-clinical individuals and COVID-19 patients are housed in the same facility. The IPS and RTLS can be used to establish geo-fencing boundaries, or virtual walls, within a facility to help ensure that individuals do not wander out of their designated areas, thereby mitigating the risk of unintentional contamination. Additionally, IPS and RTLS technologies enable contact tracing, which is the reconstruction of the chronology of movement and interactions among individuals (see Section 6 "Technology-Assisted Contact Tracing in the Hospital Setting" for additional information). This form of contact tracing is vital in scenarios involving a wide-ranging infectious disease. For example, if an infected patient were to stray into a clean zone of a shelter hospital, the tracking system would allow medical staff to accurately identify and isolate all persons that had contact with the infected patient.

A different, but no less important, application of mHealth technologies pertains to the use of mobile or portable health equipment which field hospitals often must substitute for permanently installed advanced diagnostic equipment that is used in conventional hospitals. While the functionality of these portable devices may be limited in comparison to standard hospital equipment, their portability and functionality has invaluable diagnostic utility in a field hospital. In recent years, excellent progress has been made developing portable x-ray machines, mobile ultrasound probes, magnetic resonance imaging (MRI) and computerized tomography (CT) scanners.

2.4 mHealth Solutions

Though addressing the on-going COVID-19 pandemic is the topic at hand, an opportunity also exists to consider applications of mHealth technologies to future disaster or emergency responses. In the near term, mHealth technologies could be applied to COVID-19 field hospitals to enhance the efficacy of care within these settings. Although many mHealth systems have received 510(k) clearance from the Food and Drug Administration (FDA), it is essential that pilot studies be conducted to ensure the validity and viability of the systems. This section is divided into two parts: 1) wearable sensors to monitor vital signs of personnel, and 2) tracking systems.

2.4.1 mHealth for Monitoring Vital Signs

Personnel to monitor in a medical facility can be divided into two categories:

- Medical and other facility staff who are healthy individuals, and non-medical non-infected individuals (for example, patients that have recovered or people that are not able to self-isolate).
- Patients who are infectious but experience mild to moderate symptoms.

For the first group of individuals, real-time physiological monitoring could help detect a potential symptomatic evolution of the disease from a healthy non-infected state to an infected state where the individual has mild symptoms. It is crucial to have periodic checks to ensure the health of this group. If any of these individuals were to become infected, they would be immediately isolated to minimize the spread of the virus.

Three parameters that should be monitored in this first group are: fever, heart rate (HR), and respiratory rate (RR). Fever is the most common symptom observed in new patients, followed by cough and fatigue [2]. Ideally, the best way to detect fever is through monitoring core body temperature [5]. However, an accurate measure of continuous core body temperature is not trivial to acquire. It can be obtained through rectal probes that emit temperature readings via Bluetooth to a computer-based system, or with ingestible pills like the VitalSense system

(Philips, Bend OR) [6]. Neither of these solutions are particularly practical in a clinical setting. The military has developed an algorithm to estimate core body temperature from time series HR data [7]. However, that algorithm was developed to estimate core temperature changes in response to high environmental temperatures and physical exertion. There is a possibility that in the future this algorithm could be validated and/or modified to estimate core body temperature increases related to fever, but, at the present time, this has not been done. For the above reasons, we suggest the use of skin temperature as a proxy for core body temperature, acknowledging its limitations.

The key factors to be considered in the selection of these devices are data type, data quality, battery life, and ease of use. Clinical staff remain in field hospitals for the entire duration of the pandemic, significantly longer than any patient would be expected to stay. Furthermore, due to the extreme pressure and stress they experience, the use of mHealth technology should not compound the difficulties they are already experiencing in performing their clinical duties. Possible solutions to monitor vital signs are smart watches, arm bands and bio-patches that could collect all the physiological parameters mentioned above.

In the following, we provide some examples of wearable sensors/systems that could be considered as candidates for monitoring clinical staff in a field hospital. The list of devices herein presented is by no means complete. As the devices are presented as examples of available technologies, inclusion of their description in this report should not be considered an endorsement or a suggestion that these devices might be preferable to other commercially available systems.

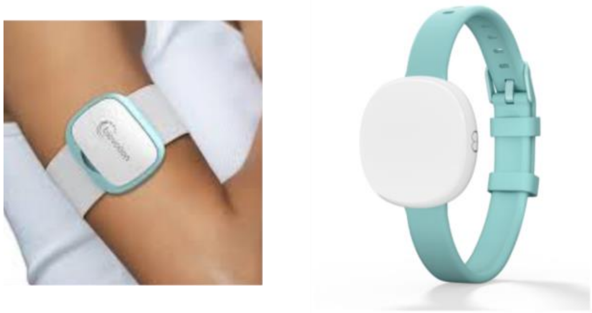

Fig. S2.2. Left: Everion arm band by Biofourmis. Right: Ava bracelet by Ava Science. (Images reproduced with permission from the manufacturers.)

The Ava bracelet (Ava Science Inc., San Francisco, CA) (Fig. S2.2) [8] is a wrist-worn sensor that has been tested in multiple research studies [9]. The Ava Bracelet was originally designed as a device to measure physiological changes across the menstrual cycle. However, the system's sensors function regardless of gender or age. The device continuously tracks breathing, skin temperature, resting pulse rate, skin perfusion, and heart rate variability (HRV) providing relevant insight into the wearer's status. However, wrist-worn devices may not be suitable for clinical staff, due to the heightened hygiene requirements of their duties. This form factor may impede or even negate hand and forearm washing. Due to this, an alternative such as the upper arm band worn Everion® (Biofourmis, Boston, MA) should be considered (Fig. S2.2). Everion® can measure HR, skin temperature, RR, peripheral blood oxygenation (SpO2), and other parameters including skin blood perfusion and blood pulse wave [10].

Bio-patches are generally more accurate than smart watches or other wrist-worn sensors and are thus consequently more applicable in cases where a precise measurement is desired (and when it is not practical nor appropriate for clinical staff to be wearing wristbands). There are two solutions in this category that have already been deployed for COVID-19 patient monitoring. These are the Biosticker™ (BioIntelliSense: Denver, CO) [11] and the Sensium® (Sensium; Oxford, UK) systems [12]-[14].

The Biosticker™ is a wearable sensor with 510(k) FDA clearance worn on the left-upper chest that provides continuous monitoring for up to 30 days; capturing HR, skin temperature and RR. All this data can be transmitted wirelessly and monitored in real-time. Although this device has not received 510(k) clearance from the FDA for applications beyond vital signs monitoring, it can provide some information about activity level and frequency of coughing, sneezing, and vomiting. These symptoms could be indicative of a relapsed patient or early signs of COVID-19.

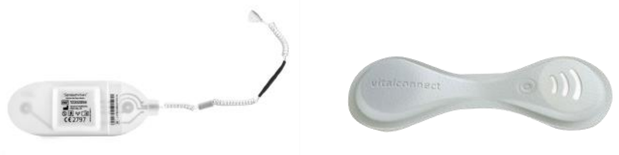

Fig. S2.3. Left: Sensium® wearable patch. Right: VitalPatch® biosensor by VitalConnect. (Images reproduced with permission from the manufacturers.)

The Sensium® wearable sensor (Fig. S2.3) monitors the wearer's temperature, HR and RR every two minutes for up to 5 days. All data can be transmitted wirelessly and viewed at a designated terminal (e.g. a nursing station) or via a dedicated smart application. This system is designed to be deployed in hospital settings and can be easily integrated into electronic medical records via industry standard Health Level 7 (HL7) protocols. However, for wireless communication, Sensium® transponders need to be placed throughout a ward or facility, increasing system cost and deployment time.

The second group of people who need to be monitored in a field hospital are those with mild to moderate symptoms. This category includes the most vulnerable people that require real-time monitoring to facilitate rapid intervention if the need arises. Due to the rapidly evolving nature of our shared knowledge of COVID-19, the selection of key clinical measures to monitor infected patients must contend with variables that are changing by the moment. However, research has converged on the following features: core temperature, electrocardiogram (ECG), blood pressure, RR, and SpO2. For these patients, measuring HR alone may not be sufficient. Rather, a single- or double-lead ECG may be needed to monitor and detect any cardiac complications that could occur with COVID-19 [15], [16].

For these patients, the Equivital™ EQ02+ LifeMonitoring system (Equivital™; Cambridge UK) [17], [18] and the VitalPatch® (VitalConnect, San Jose CA) [19], [20] may be appropriate monitoring systems. The Equivital™ EQ02+

LifeMonitor is an FDA 510(k) cleared and European CE certified wearable sensor system (Fig. S2.4) that includes an infrared skin temperature sensor, a tri-axial accelerometer, ECG, and RR. Additionally, compatible ancillary sensors can be integrated to measure: core temperature (thermometer pill), SpO2, wireless skin temperatures (skin temperature patch), and galvanic skin response (GSR). All of the recorded data is stored on an 8GB SD card, allowing data download and analysis. Data can be transmitted in real time from the sensor electronics module (SEM), via Class 1 Bluetooth, to a mobile phone or computer, for viewing and analysis. The Equivital™ Real Time Monitoring system has been used extensively by the U.S. Army to assess soldiers' physiological health [21].

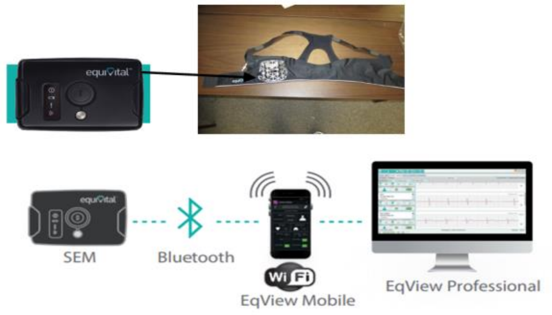

Fig. S2.4. Top: Left Sensor Electronics Module (SEM) inserts into the pocket on the harness. Bottom: from Left to Right: The SEM communicates via Bluetooth to an Android phone with Eqview Mobile installed, which then can communicate to a computer with EqView Professional installed. (Photo and graphic used with permission from the manufacturer).

The VitalPatch® is an FDA cleared biosensor developed by VitalConnect (San Jose, CA) (Fig. S2.3). This device was designed primarily for detection of heart arrhythmias. It can track up to 11 different vital signs. The system monitors real-time single-lead ECG, HR, HRV, skin temperature, and RR. Moreover, through third-party devices, this system can measure blood pressure and SpO2 for up to 120 hours. The company offers an interface platform, VistaSolution™, to visualize the data from multiple wearers with predictive analytics and an early warning system based on the National Early Warning Score (NEWS). The NEWS implementation can be tailored for different physiological states, helping to further individualize monitoring and clinical decision-making for each patient. Color-coded notifications provide both visual and numeric (0 to 20 scale) trigger levels to determine the urgency of clinical response.

2.4.2 Tracking Systems

Real-Time Locating Systems (RTLS) are an effective way of tracking personnel and equipment inside healthcare facilities. RTLS, for example CenTrak [22] and Infinite Leap [23], are already extensively used in hospitals to both track personnel and equipment (see Section 6 "Technology-Assisted Contact Tracing in the Hospital Setting" for additional information). These platforms usually require significant time and effort to deploy. As such, it may not be feasible to utilize RTLS in field hospitals where rapid deployment is essential. Ultimately, the constraints that affect the choice of tracking systems are material cost, power consumption, labor cost, and integration cost.

The type of technology utilized for tracking is of fundamental concern [24], as every platform has strengths and weaknesses. For example, infrared systems are robust against false readings, but are expensive due to the required infrastructure needed throughout the target facility. WiFi RTLS are comparatively cheaper but come with technical limitations of their own.

A solution that meets the common restrictions and needs seen in a field hospital is the Sewio system (Sewio, Brno, Czech Republic) [25]. This system exploits Ultra-Wide Band (UWB) radio pulses at very high frequency, in the order of GHz. It computes the packet's time of flight to estimate the distance between the transmitters (or anchors, positioned at known coordinates) and the receiver (or tag, positioned on the object/person to be tracked). Combining multiple units, utilizing trilateration techniques, one can estimate the location of the target with an accuracy that varies between one and thirty centimeters. UWB systems have the highest tracking accuracy but are not popular in clinical facilities. This is due to No Line of Sight (NLOS) issues, which cause measurement imprecision when obstructions occur between the anchors and the tag [26]. However, this technique has been successfully applied in open-space industrial environments, similar in structure to field hospital settings like convention centers or arenas, where it is possible to position multiple transceivers to mitigate the NLOS problem.

The Sewio's RTLS (Fig. S2.5) is based on the Decawave transceiver, which allows for the system to track human-worn tags with an accuracy of about 30 cm. Unique to this system is its advanced analytic interface, Sensmap. Sensmap is a visualization tool that allows for the creation of different zones, paths, and virtual walls. An unlimited number of zones can be set-up in great detail due to the precision of the tracking and geo-fencing functions. Alert and notification features can be easily implemented to evaluate personnel and patient position inside a facility. For additional material see Section 6 ("Technology-Assisted Contact Tracing in the Hospital Setting").

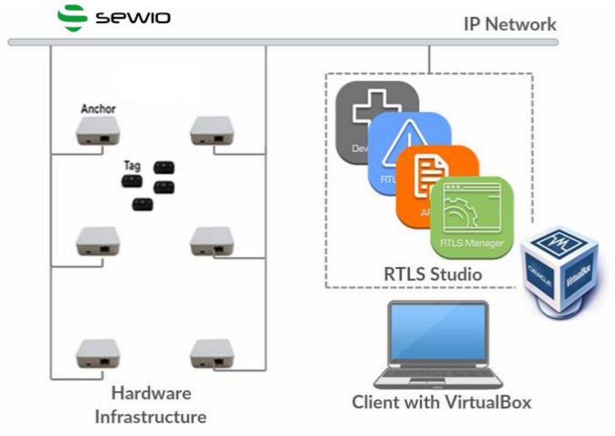

Fig. S2.5. Sewio RTLS architecture. (Image reproduced with permission from the company.)

2.5 Portable Medical Equipment

Wearable mHealth technologies capable of continuously monitoring patients or clinical personnel could be part of an early warning system. However, it is also vitally important to maintain the quality of care expected at a hospital, even in difficult circumstances. Thus, the need for advanced portable diagnostic devices. In instances of COVID-19, ultrasound scanners could be utilized to detect pleural effusion. The literature suggests that COVID-19-positive individuals display pulmonary ground-glass or ground-glass/consolidation lesions [27]. Research has shown that deep learning algorithms applied to CT or X-ray images are able to distinguish not only affected versus non-affected individuals, but also provide insight into the evolution of the disease [28]-[31]. Herein we provide details about some of the innovative portable imaging devices that could be deployed in a field hospital scenario.

A specific typology of portable devices that has shown promise when diagnosing COVID-19 are ultrasound scanners [27], [32]. The Butterfly iQ (New York, NY) [33] is an FDA-cleared lung ultrasound probe that can be used together with iOS or Android smart devices. The ultrasound chip is composed of a 2D-array of 9,000 micro machined sensors that allow a maximum depth scan of 30 cm. The device is capable of two hours of continuous scanning and has four different imaging modes: M-mode, B-mode, color Doppler and power Doppler.

An alternative solution is the Lumify [34] system by Philips, a portable ultrasound machine used by the US Army. This high-resolution imaging system is used for abdominal, lung, soft tissue, vascular, and cardiac applications. There are different types of available probes set at various frequency ranges (from 4 to 1 MHz, up to 12 to 4MHz) with differing modes of operation (2D, color Doppler, M-mode, advance XRES and multivariate harmonic imaging, SonoCT). This system is compatible with different types of Android and iOS smart devices.

An X-ray system is a valuable diagnostic tool for a variety of health issues. For field hospitals, the portable X-ray machine from OR Technology, the Amadeo M mini (Oehm und Rehbein GmbH, Germany), is a viable portable alternative to traditional machines [35]. Weighing 79 kg, the Amadeo M can be readily placed where necessary to provide the functionality, image quality, and productivity of a radiographic room at the point of care. This, in the context of COVID-19, would make this X-ray system ideal for patients unable to be taken to a traditional radiology room due to contamination concerns or other medical and logistical reasons.

For more intensive scans like CT and MRI, there are portable options available as well, such as the BodyTom [36] and the Hyperfine, respectively [37] (Fig. S2.6). The BodyTom (NeuroLogica Corp, Danvers MA, USA) is a completely portable full-body 32-slice CT scanner that features an 85 cm gantry and 60 cm field of view. The slice thickness is tunable in a range from 1.25 to 10 mm, with an image resolution of 512x512 pixels. The maximum scan length is 2 m. The integrated battery-powered motors allow a single person to move the system inside a facility. The Hyperfine system (New York NY) is a portable MRI system with motorized wheels that allows for quick deployment. This system can work with standard wall outlet power. The Hyperfine system has received FDA 510(k) clearance for MRI head scanning. Clinical trials are underway to validate the device for other applications.

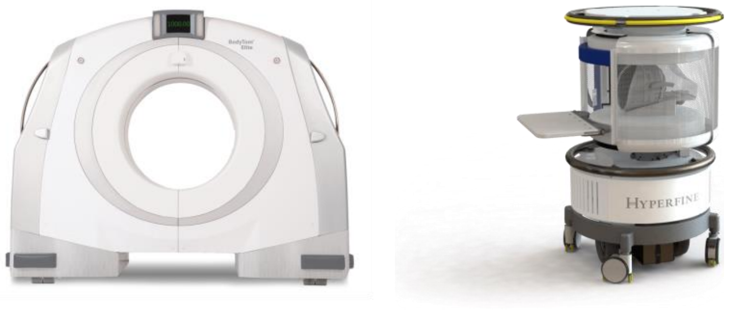

Fig. S2.6. Left: BodyTom CT scan by NeuroLogica. Right: Hyperfine portable MRI system. Images reproduced with permission from the companies.

2.6 Discussion and Conclusions

Choosing the optimal monitoring solution for a field hospital is a complicated problem, demanding a balance between available technology, facility type and location, personnel and patient populations and logistics (Fig. S2.7). Every crisis response is unique, and multiple independent factors must be considered when faced with a real case scenario. As such, the devices proposed in this section are intended as a starting point for researching and discussing the most suitable monitoring platforms for use in the COVID-19 pandemic response (for other possible solutions see Sections 4 " Remote Monitoring of Patients with COVID-19 and Frontline Healthcare Workers Using mHealth Technologies" and 5 "Emerging mHealth Technologies for Monitoring and Prevention of COVID-19" of the Supplementary Materials).

The shared experience of this pandemic, and the emergency responses deployed around the world, have shown the roles mHealth technologies could play in effectively and efficiently running field hospitals. mHealth technologies can: make simultaneous real-time monitoring of several vital signs; help ensure the safety of staff and patients through notifications of possible exposure; facilitate interaction between infected

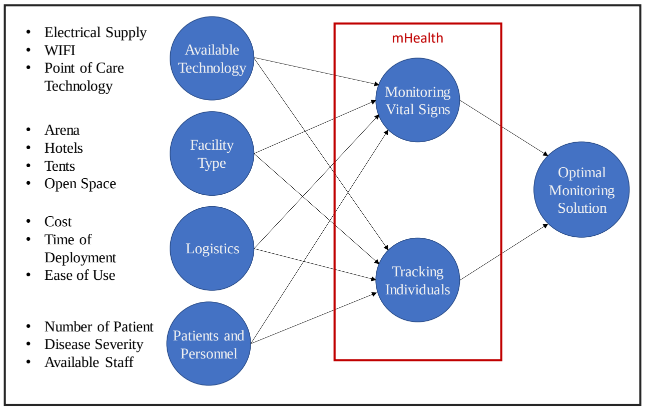

Fig. S2.7. Schematic representation of the variables affecting the choice of an optimal solution Monitoring solution in a field hospital.

individuals and clinical staff while mitigating the risk of spreading the virus; and act as a force multiplier for clinical personnel.

The adoption of these mHealth technologies will need to go hand-in-hand with the creation of new analytic techniques capable of integrating and relaying this vast amount of multi-dimensional data into useful clinical information. This is doubly important, given that new and emerging diagnostic tools are being developed in the wake of the COVID-19 crisis to diagnose infection via speech analysis [38], smell tests [39], and breath measurements [40] (for additional information, see Section 5 "Emerging mHealth Technologies for Monitoring and Prevention of COVID-19").

Abstracted from the emergency response, there is a major evolution in the Internet of Things (IoT) that may lead to the next generation of healthcare equipment; and will likely work in synergy with mHealth technologies. For example, there are already beds with embedded vital parameter monitoring [41]. Ultimately, in fighting this disease where the majority of patients are asymptomatic (or suffer only mild to moderate symptoms); field hospitals are necessary to absorb the overflow of patients from hospitals and isolate infected individuals and to slow the spread of the virus. mHealth technologies offer a viable means to aid these efforts by enchancing efficacy of improvised healthcare facilities. There are limits to addressing the immediate needs in dealing with this first wave of COVID-19, but through mHealth technologies we can be better prepared for the next wave or next pandemic by harnessing the power of real-time monitoring and wearable sensors to assist our healthcare workers by providing vital early warning decision tools.

References

[1] Center for Systems Science and Engineering at Johns Hopkins University, "COVID-19 Dashboard." Available: https://coronavirus.jhu.edu/map.html. [Accessed: 24-May-2020]

[2] B. Aylward and W. Liang, "Report of the WHO-China Joint Mission on Coronavirus Disease 2019 (COVID-19)," 2020. [Online]. Available: https://www.who.int/docs/default-source/coronaviruse/who-china-joint-mission-on-covid-19-final-report.pdf. [Accessed: 24-May-2020].

[3] S. Chen *et al*., "Fangcang Shelter Hospitals: A Novel Concept for Responding to Public Health Emergencies," The Lancet, vol. 395, no. 10232, pp. 1305–1314, April 2020.

[4] "Construction and Operation Manual of Fangcang Shelter Hospitals for COVID-19," 2020. [Online]. Available: https://gmcc.alibabadoctor.com/prevention-manual/reader?pdf=Construction%20and%20Operation%20Manual%20of%20Fangcang%20Shelter%20Hospitals%20for%20COVID-19%20Summary.pdf&no_handbook=true&opt=read&version=standard&language=en&content_id=2 [Accessed: 24-May-2020]

[5] Taylor N.A.S. *et al*., "Considerations for the Measurement of Core, Skin and Mean Body Temperatures," Journal of Thermal Biology, vol. 46, pp. 72-101, Dec. 2014.

[6] VitalSense Ingestible Pill. : http://www.actigraphy.com/solutions/vitalsense/ [Accessed: 24-May-2020].

[7] M.J. Buller *et al*., "Estimation of Human Core Temperature from Sequential Heart Rate Observations," Physiol Meas., vol. 34, no. 7, pp. 481–798, Jul. 2013

[8] Ava Bracelet: https://www.avawomen.com/ava-bracelet-for-covid-19/ [Accessed: 24-May-2020]

[9] B.M. Oodale *et al*., "Wearable Sensors Reveal Menses-Driven Changes in Physiology and Enable Prediction of the Fertile Window: Observational Study," J Med Internet Res, vol. 21, no. 4, p E13404, 2019

[10] Everion Arm Band: https://www.biovotion.com/everion/ (acquired by Biofourmis) [Accessed: 24-May-2020]

[11] Biosticker: https://biointellisense.com/ [Accessed: 24-May-2020]

[12] Sensium: https://www.sensium.co.uk/us/ [Accessed: 24-May-2020]

[13] L.M. Posthuma *et al*., "Remote Wireless Vital Signs Monitoring on the Ward for Early Detection of Deteriorating Patients: A Case Series," International Journal of Nursing Studies, vol.104, pp. 103515, Apr. 2020

[14] M.J.M. Breteler *et al*. "Vital Signs Monitoring with Wearable Sensors in High-risk Surgical Patients: A Clinical Validation Study," Anesthesiology vol.132, pp 424-439, Mar. 2020

[15] M. Madjid *et al*., "Potential Effects of Coronaviruses on the Cardiovascular System: A Review," JAMA Cardiol. 2020 [ePUB]

[16] K.J. Clerkin *et al*., "Coronavirus Disease 2019 (COVID-19) and Cardiovascular Disease," Circulation, vol. 141, no. 20, pp. 1648-1655, 2020

[17] Equivital: https://www.equivital.com/covid-19 [Accessed: 24-May-2020].

[18] Equivital Heathcare Case Study https://www.equivital.com/assets/case_studies/Healthcare_Case_Study_HIDA3330-CSHC-01-1.1_pdf_.pdf [Accessed: 24-May-2020].

[19] VitalPatch: https://vitalconnect.com/solutions/vitalpatch/ [Accessed: 24-May-2020].

[20] D.M. Levine *et al*., "Hospital-level Care at Home for Acutely Ill Adults: A Pilot Randomized Controlled Trial," J. Gen Intern Med, vol. 33, no. 5, pp 729-736, Feb. 2018

[21] W.J. Tharion *et al*., "Evolution of Physiological Status Monitoring for Ambulatory Military Applications. In: Human Performance Optimization: The Science and Ethics of Enhancing Human Capabilities," Matthews, M.D., and Schnyer, M. (Editors), Oxford, UK: Oxford University Press, pp. 142-164, 2019

[22] Centrak: https://centrak.com/: [Accessed: 24-May-2020]

[23] Infinite Leap https://infiniteleap.net/covid 19-

emergency-response-solutions/ [Accessed: 24-May-2020]

[24] D. Dardari *et al*., "Indoor Tracking: Theory, Methods, and Technologies," IEEE Transaction on Vehicular Technology, vol. 64, no. 4, pp 1263-1278, Feb 2015

[25] Sewio RTLS https://www.sewio.net/real-time-location-system-rtls-on-uwb/ [Accessed: 24-May-2020]

[26] J. Khodjaev *et al*., "Survey of NLOS Identification and Error Mitigation Problems in UWB-based Positioning Algorithms for Dense Environments," Annals of Telecommunications, vol. 65, pp 301-311, Jun. 2010

[27] Y. Huang *et al*. A Preliminary Study on the Ultrasonic Manifestations of Peripulmonary Lesions of Non-critical Novel Coronavirus Pneumonia (COVID-19)," SSRN Electronic Journal, Feb. 2020. Available: http://dx.doi.org/10.2139/ssrn.3544750 [Accessed: 24-May-2020]

[28] O. Gozes *et al*., "Rapid AI Deveopment Cycle for the Coronavirus (COVID-19) Pandemic: Initial Results for Automated Detection & Patient Monitoring Using Deep Learning CT Image Analysis". https://arxiv.org/ftp/arxiv/papers/2003/2003.05037.pdf [Accessed: 24-May-2020]

[29] A. Narin *et al*., "Automatic Detection of Coronavirus Disease (COVID-19) Using X-ray Images and Deep Convolutional Neural Network". Available: https://arxiv.org/ftp/arxiv/papers/2003/2003.10849.pdf [Accessed: 24-May-2020]

[30] L. Huang *et al*., "Serial Quantitative Chest CT Assessment of COVID-19 Deep-Learning Approach. Radiology: Cardiothoracic Imaging", vol 2, no. 2, Mar 2020 (Published Online) https://doi.org/10.1148/ryct.2020200075

[31] N. Poyaidji *et al*., "COVID-19-Associated Acute Hemorrhagic Necrotizing Encephalopathy: CT and MRI Features," Radiology, Mar. 2020 [ePUB]

[32] J. Narula *et al*., "Time to Add a Fifth Pillar to Bedside Physical Examination: Inspection, Palpation, Percussion, Auscultation and Insonation," JAMA Cardiol, vol. 3, no. 4, pp 346-350, Apr. 2018

[33] Butterfly IQ: https://butterflynetwork.com/. [Accessed: 24-May-2020]

[34] Lumify: https://www.usa.philips.com/healthcare/sites/lumify, [Accessed: 24-May-2020]

[35] Amadeo X-ray Systems: "https://www.or-technology.com/media/downloads/Brochure%20Amadeo%20M%20mini%20Systems_human_EN.pdf, [Accessed: 6 May 2020]

[36] BodyTom: https://www.neurologica.com/bodytom: [Accessed: 24-May-2020]

[37] HyperFine: https://www.hyperfine.io/ [Accessed: 24-May-2020]

[38] B.W. Schuller. et al. "COVID 19 and Computer Audition: An Overview on What Speech & Sound Analysis Could Contribute in the SARS-CoV-2 Corona Crisis", Available: https://arxiv.org/pdf/2003.11117.pdf, [Accessed: 24-May-2020]

[39] S. Moein *et al*., "Smell Dysfunction: A Biomarker for COVID-19," International Forum of Allergy and Rhinology, April 2020 [ePUB]

[40] BreathTest-1000, Available: https://www.medicaldevice-network.com/news/astrotech-screening-instrument-covid 19-infections/, [Accessed: 24-May-2020]

[41] M.Y.M Wong *et al*., "Contactless Recording of Photoplethysmogram on a Sleeping Bed," Annual International Conference of the IEEE Engineering in Medicine and Biology Society, Minneapolis MN, pp. 907-910, Jul. 2009

ePRO Solutions to Screen and Monitor COVID-19 Cases

3.1 Introduction

3.1.1 ePRO Technologies

An electronic Patient-Reported Outcome (ePRO) is a self-reported health outcome that is captured using a digital system, like a mobile application, outside of the traditional healthcare setting. In the past several years, ePRO platforms have evolved significantly to serve many different purposes in the healthcare system, including disease management, population health management, and clinical trial administration [1]. Over the past decade, ePRO methods have been successfully implemented in clinical trials, applications which range from understanding the development and history of disease, therapeutic efficacy and side-effects. For instance, ePRO technologies have been utilized to investigate the efficacy of estradiol/levonorgestrel to decrease post-menopausal symptoms; the use of eszopiclone to prevent insomnia; ketorolac to relieve ocular pain; the use of ruxolitinib to treat myelofibrosis; and the efficacy of milnacipran in fibromyalgia [2]. ePRO solutions have been also extensively used in oncology practice for symptom surveillance, postoperative monitoring, evaluation of chemo- and radiotherapy side-effects, and assessment of palliative care [3].

As mobile technologies have advanced, ePRO solutions have evolved from simple computer- and telephone-based systems to platforms available for use on smartphones, tablets, and wearable devices [4]. In a clinical context, ePRO platforms are increasingly used as a primary method of symptom reporting, for improved clinical precision and patient-provider communication [5]. In the following, we largely focus on discussing examples of ePRO platforms utilized for COVID-19 related symptom reporting. The list of examined platforms is neither meant to be complete nor to cover all relevant applications of this type of technology. For instance, herein, we do not review the COVID-19 screener released by Apple in late March, which currently provides the CDC with valuable COVID-19 data https://tinyurl.com/y8hmkut2. Also, we do not review the Babylon Health App by Google, which provides users with information and tools https://tinyurl.com/ybmh9v6x to help address, among others, COVID-19 related questions and problems. Finally, we do not review the potential use of REDCAP https://www.project-redcap.org/ to develop smartphone applications to be used in a COVID-19 context. Rather, we provide criteria for the potential evaluation of ePRO platforms that can be generally used as mobile screeners.

3.1.2 Applications for COVID-19

During the COVID-19 pandemic, ePRO platforms have assumed an important role in the discovery, monitoring, and tracing of suspected or confirmed patients [6]. In addition to symptom tracking, ePRO platforms can be utilized in the current environment to assist in telehealth consults, collection of data from wearable devices like Bluetooth-enabled thermometers, contact tracing, and geolocation of cases (Fig. S3.1). Available solutions differ in their primary orientation, ranging from platforms built to accompany provider or telehealth services; to those built for research; to platforms intended to enable patients and family caregivers to self-manage and engage in shared decision-making with providers. In the following subsections, the functionality of existing ePRO solutions and compare their capabilities in different use cases relevant to COVID-19 will be reviewed.

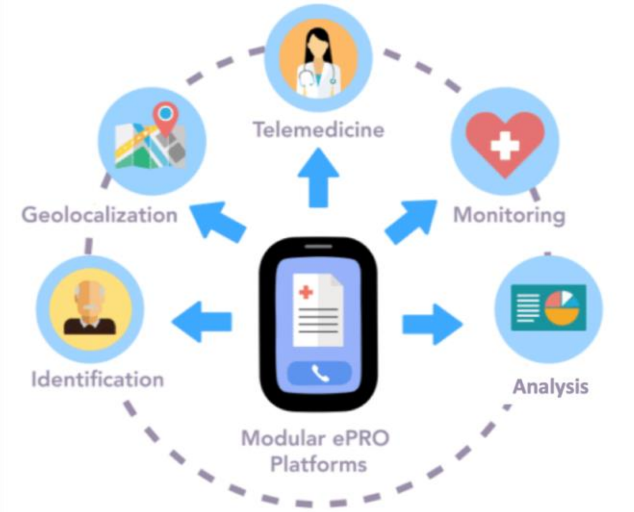

Fig. S3.1. Modular ePRO platforms are integrated with multiple features and services such as patient identification, geolocalization, telehealth, monitoring and data analysis.

3.1.3 Methodology

Commercial ePRO platforms were examined to determine their clinical utility and potential strengths for implementation during the COVID-19 pandemic. The analyzed solutions are: Think Research (Think Research Corporation, Toronto, Canada); HRS Health Recovery Solutions (Health Recovery Solutions, New York, NY); CoronaCare (CarePassport, Newton, Massachusetts; developed in collaboration with Massachusetts General Hospital); Custom M-Health App for Firefly Health (ADK Group, Boston, MA); Vital Care (VitalTech Affiliates, LLC., El Paso, TX); Folia (Folia Health, Boston, MA); Conversa (Conversa Health, Portland, OR); NoteWorth (NoteWorth, Hoboken, NJ); Twistle (Twistle, Inc., Seattle, WA); MyDataHelps COVID-19 (CareEvolution, Inc., Ann Arbor, MI); Orbita (Orbita, Inc., Boston, MA); and MyChart Care Companion for COVID-19 (Epic Systems Corporation, Madison, WI and Cleveland Clinic) (Table S3.1).

Evaluation of the general specifications of these platforms considered versatility of the solution type, availability of the

Solution	Provider	Description
**Think Research**	Think Research Corporation	App-based solution that tracks symptoms and signs, connects to wearables, provides geo-tracking, connects to telehealth services and offers clinician communication. It includes advanced analytics to enable vital reporting and data visualization to monitor the COVID-19 outbreak.
**HRS Health Recovery Solutions**	Health Recovery Solutions	App-based solution to monitor patients and provide condition-specific education, symptom surveys, medication reminders. It enables providers to monitor, triage, and care for their patients in real time directly from their office, clinic, or while on-the-go.
**CoronaCare**	CarePassport	Command center portal and patient native app. Customized multi-lingual questionnaire. Highlighted patients with escalating symptoms and prioritized on the command center worklist. Location information available. Chat, secured voice, video calls. Administrative portal to manage user role membership, bulk-messaging, edit patient-reported outcome questions.
**Custom mHealth App for Firefly Health**	ADK Group	App-based solution to track patient data like symptoms, blood sugar levels, heart rate, or water intake, which is especially useful for individuals with special health needs like diabetes or a heart condition. Through the digital transmission of patient data, healthcare providers can monitor and review their patient's health information in real-time. Patients can monitor their own data in real-time using their mobile phones or tablets.
**Vital Care**	VitalTech Affiliates, LLC.	Vital Care platform helps clinicians screen patients for COVID-19 symptoms to triage emergency care, as well as monitor patients virtually to set up care plans. VitalCare platform allows you to monitor patients' self-reported vitals with a wearable band.
**Folia**	Folia Health	Symptom, treatment, and test result reporting solution in a 90-second algorithmically defined multiple-choice format designed for ease-of-use for patients. Offers self-management, family tracking, communication with providers, disease progression analysis, and treatment efficacy. COVID-19 report (single-page) developed alongside specialist panel. Set alert thresholds for outcomes requiring clinical follow-up. Fully-identifiable patient data & action-oriented reports available for self-management and clinical use. Currently COVID-19 tracking for immunodeficient and cystic fibrosis patients.
**Conversa**	Conversa Health	Text-based chatbot for real-time patient monitoring, analytics, and engagement. Clinicians have access to a dashboard for population management of individuals enrolled in conversational programs. Transactional actions can be performed on individuals (including enrollment, opting-out, assignment), as well as reporting and monitoring on patient population progress and activities/actions taken.
**Noteworth**	Noteworth	App-based platform that enables remote patient screening, identification, treatment, education and longitudinal measuring of the conditions through symptom tracking.
**Twistle**	Twistle, Inc.	App-based solution with the ability to create and rapidly deploy surveys to collect and report patient data. The platform can perform message delivery, help with patient engagement, and monitor triage alerts.
**MyDataHelps COVID-19**	CareEvolution, Inc.	App-based solution to gather health information such as medications, medical procedures, allergies, diagnoses, lab results, and appointments of a patient. The platform includes an alert-based system for abnormal lab results, overdue vaccinations, and wearable readings.
**Orbita**	Orbita, Inc.	OrbitaCONNECT supports the creation of virtual health assistants that engage, inform, and empower patients at home to manage their health through personalized patient education and guidance.
**MyChart Care Companion for COVID-19**	Epic Systems and Cleveland Clinic	MyChart Care Companion for COVID-19 allows the management of patient health, prescription renewal, request referral authorizations for specialist appointments, and access quality health and wellness information. Doctor's office can send test results, prescription renewal requests, appointment reminders / changes, and health questionnaires.

Table S3.1. Characteristics of the examined ePRO solutions.

platform in different operating systems, functionality of the user interface, and regulatory compliance. The clinical utility and flexibility of the medical questionnaires incorporated into the different ePRO solutions was determined by analyzing the extent and relevance of the included signs and symptoms, and the degree of flexibility available to alter these endpoints. Parameters such as severity, localization, duration, and progression of signs and symptoms were considered when evaluating the quality of the clinical assessment. Features such as direct communication between patients and clinicians, geolocalization, and telehealth integration were assessed to determine their availability and interconnectivity within the same system and other services. The integration with external platforms, such as wearable technologies and electronic health records (EHR), was investigated and documented. The ability and performance of ePRO solutions to collect, transfer, visualize and analyze data was also evaluated. All the information was collected directly from company representatives or from material available on their corporate websites.

3.2 RESULTS

3.2.1 SOLUTION TYPES

ePRO solutions are available in mobile app, web app, and SMS platforms (Table S3.2). Most solutions are app-based (Android, IOS, and Web app), except for Conversa (SMS texting). Folia and Twistle offer SMS texting in addition to app-based platforms. All solutions have COVID-19 modules and are compliant with the Health Insurance Portability and Accountability Act (HIPAA).

3.2.2 PATIENT IDENTIFICATION AND MONITORING

In this subsection, thirteen ePRO solutions that have the ability to collect data for application to clinical algorithms, designed to stratify patients by level of risk of current COVID-19 infection, have been analyzed [7]. All included solutions provided robust COVID-19 modules and clinical algorithms with flexibility to rapidly update known signs and symptoms based on the latest World Health Organization (WHO) recommendations and scientific literature. All the analyzed solutions incorporate questionnaire-based triage systems that

Solution	Type	OS Support	COVID-19 Module	HIPAA Compliant
**Think Research**	App, Website	Android, IOS, WebApp	Yes	Yes
**HRS Health Recovery Solutions**	Bluetooth Devices, App	Android, IOS	Yes	Yes
**CoronaCare**	App, Website	Android, IOS	Yes	Yes
**Custom M-Health App for Firefly Health**	App	Android, IOS	Yes	Yes
**Vital Care**	App and wearable band for vital signs	Android, IOS	Yes	Yes
**Folia**	App, Website, Texting Platform	Android, IOS, WebApp	Yes	Yes
**Conversa**	Texting Platform, SMS	N/A	Yes	Yes
**NoteWorth**	App, Email	Android, IOS, WebApp	Yes	Yes
**Twistle**	Texting Platform, IVR, App	Android, IOS, WebApp	Yes	Yes
**MyDataHelps COVID-19**	App, Website	Android, IOS, WebApp	Yes	Yes
**Orbita**	App, IVR, SMS, Amazon Echo, Website	Android, IOS	Yes	Yes
**MyChart Care Companion for COVID-19**	App, Website	IOS, WebApp	Yes	Yes

Table S3.2. Solution type and operating system support. IVR stands for interactive voice support.

can be used to determine the risk of infection and help set the priority of health care responses.

These ePRO solutions have developed specialized COVID-19 modules, designed to assess relevant symptoms and identify patients' risk of current infection. Advanced mathematical analysis of the information collected using these platforms can be utilized to stratify patients based on risk, assisting in triage efforts. Clinical algorithms have proven useful in identifying patients requiring medical attention during the pandemic, particularly in the ~20% of COVID-19 cases that are symptomatic [8]. By applying clinical algorithms to data collected by ePRO systems, it will be possible to remotely monitor the current health status and clinical needs of potential and confirmed cases, as well as individuals at high risk of severe disease [9].

The analyzed solutions can collect individual data components for clinical analysis, including: demographics (age and gender), current symptoms, existing chronic conditions, and exposure to potentially infected patients (Fig. S3.2) [10].

Stratification and classification of patients by clinical condition can be completed based on the severity of symptoms and existing conditions. Symptoms analyzed may include fever, tiredness, and dry cough, as well as other less common symptoms like nasal congestion, anosmia, runny nose, sore throat, diarrhea, aches and pains [11]. Shortness of breath or respiratory distress are classified by most algorithms as severe

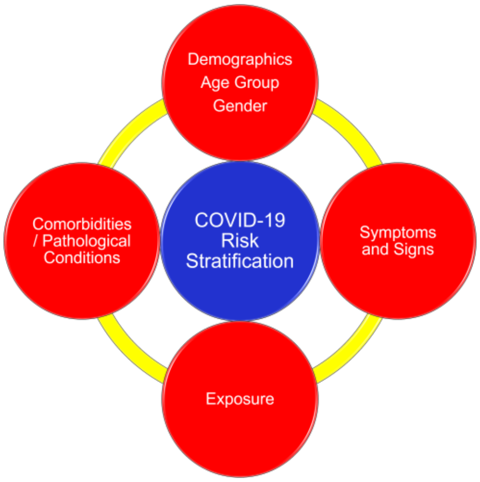

Figure S3.2. Parameters used by clinical algorithms for patient stratification and classification.

symptoms that should be promptly managed at a hospital [12]. Some existing conditions, such as chronic obstructive pulmonary disease, obesity, coronary artery disease, hypertension, diabetes, cancer and immunosuppression are known to increase the mortality of COVID-19 patients and are therefore important to be considered in the implementation of the clinical algorithms [13].

Patient stratification can also be performed by age, as current clinical knowledge on COVID-19 suggests significant variation in disease mortality and morbidity by age-group [15]. Some algorithms take into consideration geographical areas where COVID-19 is prevalent increasing the risk of exposure and subsequent infection [14].

After the algorithm is implemented and the severity of the symptoms in conjunction with risk exposure, age, comorbidities and other relevant conditions is assessed, the patient can be managed with different clinical protocols. A low-risk patient with mild symptoms can be managed with at-home recommendations and follow-up, which may require continued use of the ePRO solution, and/or a telehealth or phone consultation. A moderate-risk patient with relevant mild symptoms that are evolving in intensity may be followed up and assessed by telehealth services to determine if a hospital visit is necessary.

The presence of two out of three cardinal symptoms, defined at the time of this writing as high fever, dry cough, and

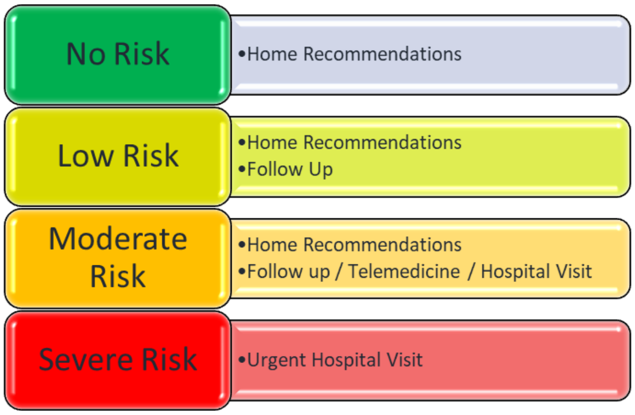

Fig. S3.3. Triage recommendations based on symptoms severity.

respiratory distress, in combination with other of the mentioned risk factors, will trigger urgent attention. This may include connecting and directing the patient to emergency services and to the closest healthcare facility (Fig. S3.3).

All the solutions presented robust questionnaire-based algorithms that included the known COVID-19 symptoms, nevertheless, one limitation that was found was the inability to enter custom symptoms by the patient, as most questionnaires have predetermined options. Some solutions allow for comprehensive symptom collection, as patients or family caregivers can elect to track any of a broad range of symptoms, behaviors, or other outcomes as part of the core platform. This aspect is relevant in new diseases as COVID-19 where new symptoms and signs are discovered on a daily basis and the integration of this functionality on ePRO solutions could play a fundamental role in disease understanding and clinical management.

3.2.3 Geolocalization Capabilities

Evidence of the effectiveness of localization tools to prevent the spread of COVID-19 has been shown in several Asian countries [14]. For this purpose, ePRO solutions were examined to also determine their geolocalization and contact-tracing capabilities. Four of the solutions (Think Research, HRS Health Recovery Solutions, CoronaCare and Custom M-Health App for Firefly Health by ADK group) currently offer precise localization and perform route tracing of patients. One interesting aspect that these solutions have, is the ability to geolocalize the surge of potential suspected COVID-19 cases geographically, alerting the surrounding hospitals to prepare for the surge and allowing the governmental authorities to implement early interventions geographically targeted. However, patients will have to individually agree to share location data with each of these services.

The above four solutions also include contact-tracing modules with the ability to track movement and integration with a notification system, which alerts the clinicians if an infected patient is in movement, and alerts an individual that has the app installed if they have been in close proximity to a potentially infected patient (suspected based on symptoms, or COVID-19 confirmed) with the same app installed. This module could be integrated with an electronic diary or passport system that could allow individuals to manually or automatically register people they have been in close proximity through data exchange between devices that have the same module installed and are in close proximity. Again, individual privacy concerns would have to be dealt with in a route-tracing model.

Other ePRO tools with native applications may be able to add localization, as they have the capability to pull location data from the mobile device. Additionally, tools that are more broadly available, like Google or Facebook, may be able to add valuable geolocalization and contact-tracing information.

Overall, geolocalization and contact-tracing data may be obtained through various sources; it is important that any ePRO solutions used to collect patient symptoms and test results are able to integrate with outside sources of contact-tracing information. A more detailed overview about contact tracing is provided in Sections 6 (“Technology-Assisted Contact Tracing in the Hospital Setting”) and 7 (“Technology-Based Contact Tracing Solutions to Contain the Spread of COVID-19 in the Community”).

3.3 Modular Integration with Additional Services and Technologies

3.3.1 Telehealth and Connected Health

Connected health, including telehealth, allows clinicians to provide patient care remotely. One of the advantages of using ePRO solutions in synergy with telehealth services, is the capacity to relieve the surge of patients in hospitals or other health services preventing saturation through remote interaction and monitoring of patients.

During COVID-19, telehealth is an attractive tool that offers the ability to monitor patients remotely by connecting them with a provider. Several of the ePRO solutions that were evaluated included telehealth integration (Table S3.3). Think Research, HRS Health Recovery Solutions, CoronaCare, Custom M-Health App for Firefly Health from ADK group, Vital Care, NoteWorth and Twistle offer integration within their platform, enabling consultation with HIPAA compliant video-conferencing services and allowing the patient to easily connect with a health provider without transferring to a second service.

Solution	Geo-localization	Contact-tracing	Telehealth Integration	Wearables Integration	EHR Integration
**Think Research**	Yes	Yes	Yes	Yes	Yes
**HRS Health Recovery Solutions**	Yes	Yes	Yes	Yes	Yes
**CoronaCare**	Yes	Yes	Yes	Yes	Yes
**Custom m-Health App for Firefly Health**	Yes	Yes	Yes	Yes	Yes
**Vital Care**	No	No	Yes	Yes	Yes
**Folia**	No	No	Integration with external services	Yes	Yes
**Conversa**	No	No	Integration with external services	Yes	Yes
**NoteWorth**	No	No	Yes	No	Yes
**Twistle**	No	No	Yes	Yes	Yes
**MyDataHelps COVID-19**	No	No	No	Yes	Yes
**Orbita**	No	No	No	Yes	Yes
**MyChart Care Companion for COVID-19**	No	No	N	No	Yes

Table S3.3. Solution features and integrations with external platforms.

3.3.2 Wearable Technologies

Wearable integration was evaluated in all the solutions and was found to be compatible in ten of them (Think Research, HRS Health Recovery Solutions, CoronaCare, Custom M-Health App for Firefly Health, Vital Care, Folia, Conversa,

Twistle, MyDataHelps COVID-19 and Orbita) as shown in Table S3.3. These ePRO platforms were found to be capable of communicating to consumer and hospital oriented devices such as vital signs sensing technologies that can record heart rate, respiratory rate, blood pressure and body temperature, as well as other systems that can record blood oxygen saturation, blood glucose, electrocardiography, cardiac implantable devices and body motion. These recorded parameters are useful to enhance the algorithm-based triage capabilities and monitoring of patients in the presented ePRO solutions. All ePRO platforms indicated flexibility to easily provide compatibility and quick configuration to new medical devices as they emerge in the market. The connectivity and communication protocol used by the solutions can be adapted to Bluetooth, wired or wireless connections depending on medical device requirements. In Section 5 ("Emerging mHealth Technologies for Monitoring and Prevention of COVID-19") of the Supplementary Materials a more detailed analysis regarding relevant wearable technologies can be found.

3.3.3 EHR Systems and Data Collection

All the evaluated ePRO solutions were found to be compatible with EHR platforms. Specific Epic system integration was reported by Think Research, HRS Health Recovery Solutions, CoronaCare, NoteWorth, Twistle, MyDataHelps COVID-19 and MyChart Care Companion for COVID-19 solutions; however, overall FHIR capability in other platforms would presumably allow for extensible integrations. One of the advantages that all platforms offer is the ability to massively implement their solution across large cities or countries. Most platforms can be deployed immediately or in a couple of weeks at the latest depending on the required customization of the service, geographical area and hospital needs. Data collection in all platforms is automated and can be visualized in real-time by customizable dashboards that can measure metrics of interest and send alerts to the clinicians. Patients can receive notifications regarding their health status in all the analyzed solutions.

Other ePRO solutions exist, and more information regarding their data integration capabilities is reviewed in Section 8 ("mHealth Data Integration Platforms") of the Supplementary Materials.

3.3.4 Covid-19 At-Home Test Functionality

Finally, as more patients receive COVID-19 antigen or antibody tests, there will be a need to collect and store test results information for each patient.

In order to conduct at-home, direct-to-consumer tests, it will be necessary to include companion apps or other means of offering instructions to individuals who are utilizing at-home tests.

3.4. Discussion

Different consumer and hospital-based solutions for disease management were presented in this section. Their ability to integrate with EHR systems and transfer real-time data make them valuable tools for monitoring suspected or confirmed infected patients. COVID-19 has led to rapid innovation and a rise in collaborative development of ePRO solutions by industry, academia and health centers. These types of digital tools have evolved and proven to be robust platforms that can be rapidly deployed in disaster scenarios to help clinicians and governments understand the surge and status of patients.

However, long-term engagement with patients can be challenging, particularly for platforms that are not primarily designed to drive value for the patient. To address this and other limitations, mobile-based ePROs could incorporate features that are intended to address primary user problems. Other solutions include implementing additional engagement modules that support behavioral modification functionalities (e.g. gaming, presenting data on behavioral improvement and positive feedback) with the aim of ultimately impacting patient outcomes by encouraging sustained adoption and reporting of their clinical state overtime. An additional strategy could be the creation of new and interactive app functionalities such as widgets for IOS and Android mobile operating systems with the aim to simplify and facilitate patient interactions with these platforms and promote their use. Promotion of patient literacy in health sciences and information technologies could also influence the success and implementation of ePRO solutions.

Even if some challenges exist, the relevance and impact that ePRO solutions can have in scenarios like the one presented during COVID-19 are apparent, as they allow clinicians to effectively identify, classify, monitor and manage non-critical patients remotely and prevent the saturation of the health care system.

References

[1] S. J. Coons, S. Eremenco, J. J. Lundy, P. O'Donohoe, H. O'Gorman, and W. Malizia, "Capturing Patient-Reported Outcome (PRO) Data Electronically: The Past, Present, and Promise of ePRO Measurement in Clinical Trials," *Patient*. vol. 8, no. 4, pp. 301-9, 2015

[2] Y. Hao *et al.*, "Clinical Trial Patient-reported Outcomes Data: Going beyond the Label in Oncology," *Clin. Ther.*, vol. 38, no. 4, pp. 811-20, 2016

[3] A. V. Bennett, R. E. Jensen, and E. Basch, "Electronic Patient-Reported Outcome Systems in Oncology Clinical Practice," *CA. Cancer J. Clin.*, vol. 62, no. 5, pp. 337-47, 2012

[4] L. M. Wintner *et al.*, "Evaluation of Electronic Patient-Reported Outcome Assessment with Cancer Patients in the Hospital and at Home eHealth/ Telehealth/ Mobile Health Systems," *BMC Med. Inform. Decis. Mak.*, vol. 15, 110, 2015

[5] C. Steele Gray, A. Gill, A. I. Khan, P. K. Hans, K. Kuluski, and C. Cott, "The Electronic Patient Reported Outcome Tool: Testing Usability and Feasibility of a Mobile App and Portal to Support Care for Patients With Complex Chronic Disease and Disability in Primary Care Settings," *JMIR mHealth uHealth*, vol. 4, no. 2:e58, 2016

[6] F. Scotté, E. Minvielle, O. Mir, F. André, F. Barlesi, and J.-C. Soria, "A Patient Reported Outcome Platform, a Useful Tool to Improve Monitoring and

Effective Management of Covid-19-positive Patients with Cancer,” *Eur. J. Cancer*, vol. 132, pp. 1–4, Apr. 2020

[7] A. Gasmi, S. Noor, T. Tippairote, M. Dadar, A. Menzel, and G. Bjørklund, “Individual Risk Management Strategy and Potential Therapeutic Options for the COVID-19 Pandemic,” *Clin. Immunol.*, vol. 215: 108409, 2020

[8] G. Xu *et al.*, “Clinical Pathway for Early Diagnosis of COVID-19: Updates from Experience to Evidence-Based Practice,” *Clin. Rev. Allergy Immunol.*, pp. 1–12, Apr. 2020

[9] O. L. Aiyegbusi, “Key Methodological Considerations for Usability Testing of Electronic Patient-Reported Outcome (ePRO) Systems,” *Quality of Life Research*. vol. 29, no. 2, pp. 325-333, 2020

[10] K. M. Gostic, A. C. R. Gomez, R. O. Mummah, A. J. Kucharski, and J. O. Lloyd-Smith, “Estimated Effectiveness of Symptom and Risk Screening to Prevent the Spread of COVID-19,” *Elife*, vol. 9: e55570, 2020

[11] Z. Y. Zu *et al.*, “Coronavirus Disease 2019 (COVID-19): A Perspective from China,” *Radiology*, 2020 [ePUB]

[12] E. S. Kim *et al.*, “Clinical Course and Outcomes of Patients with Severe Acute Respiratory Syndrome Coronavirus 2 Infection: a Preliminary Report of the First 28 Patients from the Korean Cohort Study on COVID-19,” *J. Korean Med. Sci.*, vol. 35, no. 13, pp. e142–e142, Apr. 2020

[13] P. Kakodkar, N. Kaka, and M. N. Baig, “A Comprehensive Literature Review on the Clinical Presentation, and Management of the Pandemic Coronavirus Disease 2019 (COVID-19),” *Cureus*, vol. 12, no. 4, pp. e7560–e7560, Apr. 2020

[14] M. N. Kamel Boulos and E. M. Geraghty, “Geographical Tracking and Mapping of Coronavirus Disease COVID-19/Severe Acute Respiratory Syndrome Coronavirus 2 (SARS-CoV-2) Epidemic and Associated Events Around the World: How 21st Century GIS Technologies Are Supporting the Global Fight Against Outbreaks and Epidemics,” *Int. J. Health Geogr.*, vol. 19, no. 1: 8, 2020

Remote Monitoring of Patients with COVID-19 and Frontline Healthcare Workers Using mHealth Technologies

4.1 INTRODUCTION

Severe acute respiratory syndrome coronavirus 2 (SARS-CoV-2) is a highly infectious virus that is at the root cause of coronavirus disease 2019 (COVID-19). COVID-19 can lead to severe acute respiratory infections with symptoms which include dry cough, fever, shortness of breath, fatigue, and loss of smell and taste [1]–[7]. Since its emergence in December 2019, the spectrum of COVID-19's severity has been demonstrated to be quite broad. A significant number of those patients who tested positive for the disease (approximately 80%) were either asymptomatic or experienced mild pneumonia and recovered without intervention [8]. However, about 15% of patients developed complications, including persistent high fever, respiratory distress, and hypoxemia [2], [3], [9]. These individuals often required acute care which included oxygen therapy, mechanical ventilation, and other therapeutic interventions. A small percentage of these cases became critically ill and experienced respiratory failure, sepsis, and multiple organ failure.

The high infection rates of SARS-CoV-2 has put healthcare systems at risk of being overwhelmed with COVID-19 cases. Remote monitoring of those individuals who are at risk of or have developed COVID-19 using mobile health (mHealth) technologies may help mitigate some of the burden on the healthcare system.

This section introduces home-based remote monitoring strategies applicable to patients and frontline healthcare professionals.

First, remote home monitoring will enable important health scanning and screening interventions for low- and high-risk populations that are either self-isolating or quarantined due to a positive or presumptive positive diagnosis. There would also be value in such monitoring during post-discharge recovery. To this end, by employing these technologies, hospital resources that were diverted to these less severe patients could be redeployed to focus on those in more dire circumstances; as well as reduce the likelihood of clinical staff being unexpectedly exposed to the virus.

Second, early exposure detection in frontline healthcare workers prior to the peak of viral shedding could enable them to self-isolate to avoid further spreading the infection. Early identification will also allow these healthcare workers to seek out and receive timely care, which will hopefully allow for a faster recovery, and a sooner return to continuing their work in supporting the healthcare ecosystem. Moreover, the use of mHealth systems could help assuage some of the anxiety expressed by healthcare workers concerning their exposure to the virus and risks of infection [10].

This report presents the attributes of mHealth technologies that can be used to remotely monitor COVID-19 patients and frontline healthcare workers so as to address the immediate clinical needs and concerns of these groups using solutions currently available in the commercial market. Later in this section, future research directions for mHealth technologies are discussed that could address key gaps present in the available technology.

4.2 METHODS

To investigate the feasibility of monitoring the physiological and physical variables relevant to the COVID-19 symptom complex outside the hospital, a survey of existing home monitoring solutions was conducted. A list of all the technologies considered in this project is shown in Section 4.5. Fig. S4.1 provides a schematic representation of the technology assessment process that was leveraged to review mHealth technologies. The general criteria and attributes that were used to assess these mHealth technologies were 1) symptom monitoring in low-risk, high-risk, and post-discharge patients; and 2) early detection of infection in frontline healthcare workers that are either presymptomatic or asymptomatic. In addition to assessments based on the potential utility to the above at-risk populations, the differences between those

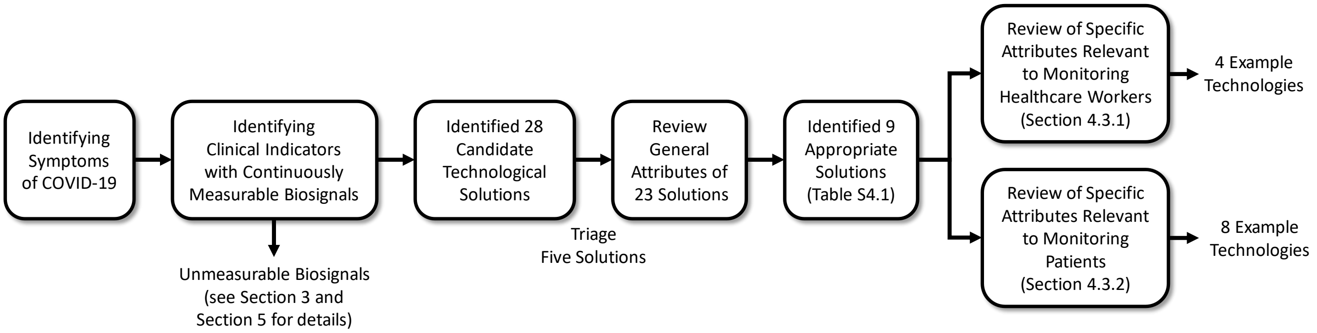

Fig. S4.1. A Schematic representation of the assessment process for mHealth technologies.

subpopulations based on the primary symptom monitoring modalities; third party regulatory approval/clearance or clinical efficacy assessment; ease of use; data storage and communication platforms; and their relative access to the monitoring technologies (see Table S4.1) were also evaluated.

4.2.1 Attributes for Assessing Technologies

4.2.1.1 Measurement Capabilities

Primary clinical characteristics of patients affected by COVID-19 include fever (i.e. axillary temperature > 38˚C [11]), cough, production of sputum, fatigue, dyspnea, muscle pain, headache, hypoxemia, high systolic blood pressure (BP), diarrhea, hemoptysis, and chills [1]–[4].

First, the characteristics that could be continuously monitored using mHealth solutions vs. the characteristics that could be more reliably captured using ePRO solutions were identified. The clinical characteristics that were deemed appropriate to be monitored using mHealth technologies included fever, cough, fatigue, dyspnea, hypoxemia, and systolic blood pressure. In contrast, the production of sputum and hemoptysis, muscle pain, headache, diarrhea, and chills were determined to be better captured using ePRO solutions and thus omitted from further analysis. Readers are referred to Section 3 ("Using ePRO Solutions to Screen and Monitor COVID-19 Cases") for a review of ePRO technologies.

Primary physiological parameters that could be relevant to or indicative of the above-mentioned clinical variables and monitored using existing on- and/or off-body sensing technologies include body temperature (fever), number and character of coughs (cough), motion or activity level (fatigue), respiratory rate (RR; dyspnea), oxygen saturation level (SpO_2; hypoxemia), and BP (high systolic BP). In addition, heart rate (HR), HR variability (HRV), and electrocardiogram (ECG) data were considered, because there is increasing evidence of a primary effect on cardiac function during late incubation, early prodromal, illness, decline, and convalescence stages of the disease. Table S4.1 includes immune and cardiac functions together since immunological responses, such as systemic release of pro-inflammatory chemokines/cytokines and afferent signaling to the central nervous system, has been observed to measurably affect cardiac signals in infected subjects [12].

4.2.1.2 Technical/Clinical Validation & Health Authority Clearance/Certification

A variety of technology implementations of particular sensing modalities have begun to emerge. For example, body temperature may be estimated with an ingestible sensor or on the surface of the body via a patch worn on the chest or in the axilla. Heart rate may be monitored through an analysis of biopotential measurements made on the chest or by photoplethysmographic techniques leveraging one or several wavelengths of light from devices positioned on the wrist, arm, or chest [13], [14]. The suitability of particular solutions for home monitoring of suspected or confirmed COVID-19 cases should be based on evidence of technical validation (sensing and engineering performance) as well as clinical validation (efficacy and safety). In this work, three levels of evidence to support a solution's state of validation were considered: 1) peer-reviewed publications documenting the clinical-research use of a technology; 2) peer-reviewed publications documenting studies specifically of technical or clinical validation against a gold standard technology; and 3) health authority clearance/certification of the solution (e.g. FDA 510(k) clearance, CE certification).

4.2.1.3 Ease of Use

For a mHealth measurement solution to be effective in its intended use, it must seamlessly integrate within the daily routine of the end-user. In this regard, a number of human factors must be considered when evaluating mHealth technologies [15]. And these human factors must be analyzed from the perspective of the users of the technology. Issues such as the form factor, physical appearance, comfort, usability, simplicity, burden, and perceived utility are important to consider. Related to these are technical issues, such as battery life and charging requirements; materials used that can cause skin irritation; bandwidth requirements for data transfer;

Subpopulation	Monitoring Modalities	Regulatory Clearance/Certification	Ease of Use	Data and Communication	Access/Availability of Technology
Clinical Personnel	• Fever detection<br>• Respiratory function<br>• Cardiac, immune function	• FDA or CE required; Clinical efficacy data considered	• Night use<br>• High user compliance and training	• Wireless<br>• Automatic uploading	• High cost<br>• Lower volume<br>• Specialized and universal platforms
High-risk Population	• Respiratory function<br>• Cardiac, immune function	• FDA or CE required; Clinical efficacy data considered	• Data/night use<br>• Mid-level user compliance and training<br>• High durability function	• Wireless<br>• Automatic uploading<br>• Bi-directional<br>• Self-report	• High cost<br>• Middle volume<br>• Specialized and universal platforms
Low-risk Population	• Fever detection<br>• Respiratory function	• FDA or CE required; Clinical efficacy data considered	• Day/night use<br>• Low user compliance<br>• High durability	• Wireless<br>• Automatic uploading<br>• Daily uploads<br>• Self-report	• Lowest cost<br>• Highest volume<br>• Universal platforms and support

Table S4.1. Population groups and assessment attributes

availability of educational material; end-user support processes; and the overall resilience/robustness of the technology.

4.2.1.4 Integration Flexibility with Existing Hospital Systems

The degree to which outputs from mHealth technologies (raw data or derived clinical parameters) can be integrated with existing hospital Electronic Medical Records (EMR) systems is an important consideration. Information regarding the availability of Application Programming Interfaces (APIs) or Software Development Kits (SDKs) to enable data transfer and integration was sought and collected. Readers are referred to Section 8 ("mHealth Data Integration Platforms") for detailed information concerning data integration into existing platforms (e.g. compliance with the Health Level Seven (HL7) standards).

4.2.2 Specific Attributes According to Use Case and Population

With the criteria established, they were stratified according to two use scenarios: 1) early detection of exposure in frontline healthcare workers and 2) remote clinical management of individuals diagnosed with COVID-19. For individuals diagnosed with COVID-19, further consideration was paid to the requirements needed from solutions according to the level of clinical risk for developing severe illness (i.e. low vs. high).

4.2.2.1 Monitoring Clinical Progression in Patients

Due to the level of infectiousness seen in COVID-19, many healthcare systems risk becoming overwhelmed were they expected to manage all COVID-19 cases at healthcare facilities. However, the severity of the disease is broad, and many patients may recover without the need for acute care. Populations at risk for developing severe illness include those who are over the age of 65; living in a nursing home or chronic care facility; and/or those who have particular comorbidities including severe heart conditions, chronic lung diseases, obesity, hypertension, diabetes, or immunocompromised states [16], [17]. The goal of remote monitoring is to enable patients with mild symptoms to keep out of hospitals while still providing healthcare professionals the information needed to detect those patients whose condition may rapidly deteriorate in order to prioritize those for intensified care regimens. The highest priority should be given to patients that show symptoms that indicate the onset and/or worsening of dyspnea, ARDS, and have sustained high fever. Accordingly, technologies deployed for this purpose should prioritize the ability to monitor RR, HR, SpO2, and core body temperature.

Clinical risk is a critical factor affecting a wide range of further considerations; including the level of technical and clinical validation required of a system; unit costs; temporal patterns of use; compliance; and data quality and transfer requirements. In high-risk populations, measurement capabilities, data quality, reliability, technical and clinical validation may very well be more important considerations than cost, ease of use, and access. Alternatively, in lower-risk populations, it may be acceptable to rely on more widely available, lower-cost solutions that may offer fewer measurement capabilities; in this population, ease of use may be of greater concern in order to maximize the likelihood of adherence to the technology.

Additionally, there will be a need for independent wireless communication (i.e. for patients without a WiFi internet connection), and integration with standard mobile platforms and third-party support will be important for implementation and consistent compliance. This will also allow patients to include daily self-report information in established ePRO systems.

There are many challenges related to monitoring patients in different contexts. Monitoring may take place in patients' homes or in dedicated isolation facilities (e.g. hotels and repurposed dormitory facilities). The setting will greatly impact patients' sense of agency and control. There are varying levels of user compliance and preferences for day vs. night use, and durability (e.g. IP 57/54 for waterproof/showerproof certification). Ambulatory artifacts due to long-term wear, sweat, electrical/acoustical interference are also notable factors.

4.2.2.2 Early Detection of Exposure Among Healthcare Workers

During an outbreak, frontline healthcare workers are vulnerable to infection due to inevitable, close interactions with ill patients. Hence, the primary goal of monitoring frontline healthcare workers is to detect signs (presymptomatic) that are indicative of infection during the incubation period (i.e. the interval between the date of contact of transmission to the first onset of symptoms). This would allow healthcare workers to self-isolate early and/or receive immediate care.

The hypothesis that supports the feasibility of early detection of COVID-19 infections during the incubation period builds on the described progression of the disease which starts with subtle changes in skin temperature, RR, SpO2, HR, ECG, etc. (as described in Section 4.2.1.). There is supporting evidence that physiological characteristics (e.g. ECG, hemodynamics, and temperature) in non-human primates exposed to different pathogens (e.g. Ebola) allow for early detection of the infection (i.e. an average of 51 hours earlier than the onset of prominent symptoms) with an Area Under the Receiver Operating Characteristic Curve (AUROC) of 0.95 [12].

Three initial options for early detection of exposure among healthcare workers that were considered consisted of a) station-based periodic monitoring in the hospital setting, b) continuous monitoring in the hospital setting (during 12-hour shifts), and c) off-shift home monitoring. The approach to periodically monitor healthcare workers by requiring them to visit a designated station equipped with assessment tools was not further investigated as many hospitals have already incorporated protocols to assess major symptoms of personnel upon entering the facility. Wearable sensing solutions that could interfere with healthcare workers' movement and/or be subject to confounding due to personal protective equipment (e.g. thermally-encapsulating effects that artificially raise body temperature readings) were deemed impractical and subsequently discarded as a means of continuous monitoring. Home monitoring was the preferred solution as it is

Product	Manufacturer	Type	Skin Temp			Resp. Rate			SpO2			Heart Rate			ECG
			MC	FDA	PR	MC	FDA	PR	MC	FDA	PR	MC	FDA	PR	
BioSticker [33]	BioIntelli-Sense	Patch on chest	Yes	Yes	NA	Yes	Yes	NA	NA	NA	NA	Yes	Yes	NA	NA
VitalPatch [34],[35],[36]	VitalConnect	Patch on chest	Yes	Yes	CV	Yes	Yes	CV	NA	NA	NA	Yes	Yes	CV	Single
BioSensor 2A [37]	LifeSignals	Patch on chest	Yes	PD	NA	Yes	PD	NA	Yes	PD	NA	Yes	PD	NA	Single
Sensium [18], [19], [24],[38]	Sensium	Patch on chest	Yes	Yes	CV	Yes	Yes	CV	NA	NA	NA	Yes	Yes	CV	Single
SensoRING [39]	Sensogram	Finger-worn	NA	NA	NA	Yes	NA	NA	Yes	NA	NA	Yes	NA	NA	Single**
Current Health [40]	Current Health	Upper Arm-worn	Yes	Yes	CV	Yes	Yes	NA	Yes	Yes	NA	Yes	Yes	NA	NA
Everion [41]–[47]	Biofourmis	Upper Arm-worn	Yes	*	Yes	Yes	*	NA	Yes	*	NA	Yes	*	CV	NA
EQ02 Life Monitor [7],[48],[49]	Equivital	Chest Strap	Yes	Yes	Yes	Yes	Yes	Yes	NA	NA	NA	Yes	Yes	Yes	Multi
Zephyr [50]-[53]	Zephyr	Chest Strap	Yes	NA	NA	Yes	Yes	Yes	Yes	NA	NA	Yes	Yes	Yes	NA

MC: Manufacturer's Claim - **FDA**: FDA 510(k) Cleared - **PR**: Peer Reviewed
* Europe (CE 0123) ** It requires that subjects place one finger of the contralateral hand on the ring.

Yes	NA: Not Available	PD: Pending	CV: Clinically Validated	Single: Single-Lead ECG	Multi: Multi-Lead ECG

Table S4.2. Example mHealth technologies with third party evaluations for measurable biomarkers associated with COVID-19 symptoms

considerably more controlled and less obtrusive (e.g. especially if monitored during sleep).

The usability of mHealth technologies and human factors must also be carefully considered when identifying technological solutions to monitor early signs of infection during sleep. Measurements during sleep might provide additional insights and may be the only time to monitor health workers effectively. However, while the usability should be optimized, the urgency and importance of effective monitoring of health condition in frontline healthcare workers, especially during the critical time period of a pandemic outbreak, may outweigh some of the factors (e.g. physical appearance, comfort, or simplicity).

4.3 Results

Twenty-eight candidate solutions were identified as part of the survey of existing technologies (see Section 4.5 for the full list). An initial evaluation was performed based on publicly available information from the manufacturers with the goal of excluding devices that provide a minimal number of biosignals (e.g. supporting the measurement of only one or two parameters). To note, some sensors were able to measure additional biosignals if add-on components could be attached to the main module (e.g. a fingertip pulse oximeter connected to the main module in a wireless or wired manner to support SpO2 measurements). In this section, only biosignals that could be measured by the main sensing module were considered when evaluating the technology.

The primary goal was to identify a minimal set of technological solutions that could measure the physiological parameters discussed in Section 4.2.1. This process resulted in five solutions. The remaining solutions were further reviewed for the above-mentioned general attributes, such as the FDA clearance and CE certification; evidence of clinical/technical validation; evident issues related to the usability (e.g. requiring wired sensors positioned at multiple body parts); and practicality (e.g. battery life less than 9 hours). Consequently, a total of nine solutions that met the functional criteria for the purpose of remotely monitoring patients and healthcare workers in their home settings were identified, which are summarized in Table S4.2. Further details of these technologies are provided in Sections 4.3.1 and 4.3.2.

It is noteworthy that technologies listed in Table S4.2 that obtained FDA registration did so via the 510(k) process. The technical/clinical validation and FDA clearance/CE certification for the measurement of activity level was considered for devices during the review process. However, information was only readily available for a small number of these devices. While it is well known that devices can quantify the activity level using a variety of metrics, their use for this

purpose among individuals with COVID-19 needs to be established, and none of the devices considered herein make regulatory claims of an ability to diagnose or monitor fatigue. Hence, the criteria related to activity level was not included in Table S4.2.

The authors would like to emphasize that it is not the purpose or intention of this Section of the Supplementary Materials to make any recommendation of particular technologies. Rather, the aim is to provide examples of mHealth solutions that have the potential to monitor patients with COVID-19 and frontline healthcare workers in remote settings.

4.3.1 Technologies for Pre-Diagnostic Scanning and Screening of Frontline Healthcare Workers

The BioSensor 2A by LifeSignals (Freemont, CA); VitalPatch by VitalConnect (San Jose, CA); Sensium by Sensium (Oxford, UK) [18], [19]; and EQ02 LifeMonitor by Equivital (Cambridge, UK) have been identified as examples of technological solutions that could be used to monitor healthcare workers to detect possible early signs of disease during the incubation period. These solutions not only support measuring body temperature and RR but also provide a comprehensive set of other measurements such as heart rate and ECG that may capture subtle physiological changes during the presymptomatic phase. It is noteworthy that among these four sensors, BioSensor 2A was the only solution that supported SpO2 measurements.

BioSensor 2A, VitalPatch, and Sensium are thin, wireless sensors encapsulated in a water-resistive, flexible enclosure that can be patched to the user's chest. All devices are Class II medical devices according to FDA guidelines and are capable of monitoring HR, HRV, RR, skin temperature, motion, and single-lead ECG. BioSensor 2A is capable of measuring SpO2 without any additional add-on devices. VitalPatch supports SpO2 via a third-party add-on. All sensors support a battery duration of five to seven days. BioSensor 2A and VitalPatch are single-use devices that need to be disposed after each use. Sensium, on the other hand, can be reused if the electrodes are replaced. All systems allow simultaneous monitoring of a large (theoretically infinite) number of individuals and support interoperability to existing EMR platforms. BioSensor 2A supports the interoperability via open APIs, while VitalPatch and Sensium support the HL7 standard. EQ02 LifeMonitor leverages a chest strap to place the rigid sensor on the human body. Similar to the solutions discussed above, EQ02 LifeMonitor measures HR, HRV, RR, skin temperature, and motion. It was the only solution that supports two-lead ECG, as well as SpO2 monitoring and core body temperature measurements through add-on sensing components. The system also supports interoperability by providing proprietary SDKs and supporting the HL7 standard. However, the usability of the system's wearable form-factor (e.g. chest strap) may not be optimized for monitoring during sleep.

4.3.2 Specific Attributes According to Use Case and Population

When identifying candidate monitoring technologies, different sets of target attributes for 'low-risk' and 'high-risk' contexts were defined (see Section 4.2.1). In doing so, it was determined that such classification of the risk level was not always a binary issue and clinical judgment was important on a case-by-case basis. The distinction between low- and high-risk case scenarios was based on factors, such as the range of measurement targets considered necessary; the degree of measurement fidelity needed (and associated regulatory approval): the quality of the data transfer pipeline: the level of interoperability and capability for integration with hospital EMR systems; unit costs and supply capability; and human factors.

Among the manufacturers of the nine technology solutions identified in Table S4.2, nearly all claim an ability to monitor skin temperature, HR, and RR. Five solutions offer an ability to monitor oxygen saturation, while only two solutions, Current Health by Current Health (Edinburgh, Scotland) and BioSensor 2A by LifeSignals (Freemont, CA) have received or are awaiting FDA clearance/CE certification for this purpose. These solutions may be particularly suited to monitoring high-risk populations as both enable continuous passive monitoring. Current Health offers EMR integration through the Fast Healthcare Interoperability Resources (FHIR) and HL7, a patient hub that enables wireless data transmission via cellular networks, and compatibility with Android and iOS platforms for additional patient engagement through text and video-based telehealth visits.

In low-risk application scenarios, we started with the same assumption that skin temperature, HR, RR, and SpO2 are the most important measurement targets. SensoRing by Sensogram Technologies (Plano, TX), Zephyr System by Medtronic (Fridley, MN), and EQ02 LifeMonitor by Equivital (Cambridge, UK) were identified as viable example solutions. Of these, only SensoRing offers the capability to measure SpO2 without an add-on device. The LifeMonitor and Zephyr systems use chest straps and appear to be targeted towards use in performance measurement in sport or military settings, though Equivital (LifeMonitor) does make reference to supporting the fight against COVID-19 on their website.

SensoRing appears to offer a lot of advantages in terms of form factor and ease of use. However, it only has a four-hour battery life in continuous mode that can be extended to 24 hours in the 'scheduled' mode. It is also a stand-alone consumer-grade device with no capability of integration with EMR systems. Despite these shortcomings, the SensoRing does offer a promising means of monitoring low-risk cases, but would require that the user has a strong grasp of digital and health literacy to make the most of the self-monitoring and to understand when to seek appropriate care.

Another sensor that holds promise in the low-risk category is the Everion sensor from Biovotion (now Biofourmis, Boston MA). The Everion sensor is worn on the upper arm and has a

low-profile form factor. It is designed to be worn for an entire week and has achieved the CE certification in Europe. Everion measures skin temperature, HR, activity, RR, and SpO2. Its measurement capability for RR and SpO2 however appears to be limited in ambulatory users due to motion artifacts. Nonetheless, its performance at rest appears to be reliable. Though accurate measurements of RR and SpO2 are only provided when at rest, SpO2 may not be required in low-risk cases, and a larger range of sensing solutions can be recommended. Such candidates would include the Biosticker from BioIntellisense and the VitalPatch from VitalConnect. Both offer the ability to perform multi-day monitoring of skin temperature, HR, respiratory rate, activity, and ECG in the form of a relatively comfortable wearable patch; as well as being capable of EMR integration.

4.4 Discussion

4.4.1 Technology Gaps and Integration Needs for Successful System Implementation and High User Compliance

Advances in mHealth technologies summarized in this section provide powerful sensing capabilities with a broad range of applications for remote monitoring. While the application of various wearable biosensors on patients and healthcare professionals are encouraging, there are several challenges that still remain. Specifically, there is no clinical validation to link the physiological biosignals considered in this Section to the underlying pathophysiology that are specific to COVID-19. Although there are current COVID-19 physiological collection studies in progress [20], [21], there are no published results with time course serological testing to correlate any changes in physiological biosignals with disease progression. As a result, more clinical studies are necessary beyond the regulatory certifications to identify biophysical, electrophysiological, hemodynamic, thermal, and neuromusculoskeletal features that are critical to facilitate early detection of infection.

The development of a comprehensive profile of both physiological and biochemical signals may require multimodal integration of sensors, with high sensitivity, proper biocompatibility, and sufficient biomechanical coupling with the human body. A key area of future work, particularly for patch-based sensors, includes the biological interface with the skin and novel ways to support long-term wear without causing significant skin reactions or signal disruption in highly dynamic modes of use [22]. Achieving intimate skin coupling with wearable devices that are untethered and simultaneously soft and mechanically robust constitute compelling directions, which could have direct implications on sensor performance and user compliance.

Some measurable indicators may also require further technological advancements and validation to establish acceptable accuracy for COVID-19 study cohorts. For example, there are established algorithms from the heat illness research community for the estimation of human core body temperature from sequential heart rate observations [23]. More recent research has shown greater accuracy for these heart rate-based core temperature estimates to predict heat illness when combined with skin temperature and neuromotor incoordination indicators [24]. However, the core temperature estimation for heat injuries is modeled on the body's ability to thermoregulate in hot, humid environments through circulatory convection losses via the skin vasculature while at the same time generating heat in muscle tissue through movement and force generation. These bounding assumptions are less applicable to the envisioned scenarios of home-based COVID-19 monitoring presented in this paper.

4.4.2 Limitations

The material presented in this section was motivated by the stated clinical standard of care for diagnostic indicators associated with accepted symptoms of COVID-19 (see Section 1 "COVID-19 Related Clinical Issues, mHealth Technology Applications, and the Acceleration of the Digital Health Transformation" and Section 4.2.1). Due to the urgency of the crisis, and the need to provide an overview in a timely manner, it is recognized that not all available mHealth solutions may have been weighed for consideration when this document was prepared. While this approach has yielded a list of mHealth technologies that can measure biosignals associated with COVID-19 clinical indicators, it does not permit an objective assessment of the technical/clinical efficacy and safety of those technologies. Due to that lack of objective, third-party validation, this survey has heavily relied on the manufacturers' claims and specifications; peer-reviewed assessments in the laboratory and clinical environments; and regulatory certifications and clearances. It should be noted, however, that certain regulatory clearances (e.g. FDA 510(k)) can be based on a series of predicate devices. For those cases, the initial clearance may be based on measuring the biosignals in a specific part of the body and could lead to less accurate use in real-world conditions at different body locations (e.g. pulse oximetry differences between fingertip-based and wrist-based devices). There are similar limitations associated with end-user implementation such as wearing a wrist-based sensor too loosely which can lead to poor photoplethysmographic (PPG) measures or incompliance due to discomfort, usability, and/or durability (e.g. taking devices off at night or portions of the day during strenuous conditions).

There are several other limitations that should be highlighted. All parameters are generally useful for the categories of home monitoring indicated in this Section. Requirements on specificity in distinguishing other flu-like symptoms need to be more stringent for the non-diagnosed, possibly presymptomatic healthcare workers. Further limitations that were not taken into account for each solution include the variability of manufacturers to scale the production in order to meet the sudden rise in demand during a pandemic and the availability of the technology for low-income populations. Moreover, some patients and healthcare workers may have pre-existing health conditions that may help them to gain access to similar technologies from their healthcare provider. Yet even in those cases, external validation must occur to ensure that similar technologies can provide the specificity for diagnosing COVID-19-associated symptoms. Time-of-day usage was also widely discussed (e.g. sleep vs.

non-sleep), and device accuracy can be dependent on the usage environment. Lastly, potential synergies from the simultaneous use of multiple devices was only briefly mentioned, although Table S4.2 shows several devices with different measurement modalities.

4.4.3 SUGGESTED FUTURE WORK

While this report identifies currently available solutions, it warrants further investigation into the required technical/clinical specifications and system designs for each of the potential user groups (i.e. healthcare workers and low-/high-risk patients after discharge), such as the specificity and sensitivity and various human factors for real-world use. Such investigation will enable trade-space decisions about near-term technology capabilities, establish system architecture and design requirements, and support clinical validation of the efficacy of mHealth systems that meet those requirements. System-level computing infrastructure needs that are related to storage, analysis, communication, and information security (e.g. individual privacy) will be important for the success of the implemented solutions. Many of the communications, data storage, and hospital system integration requirements are addressed in Section 3 "Using ePRO Solutions to Screen and Monitor COVID-19 Cases" and Section 8 "mHealth Data Integration Platforms".

Thoracic impedance measurements are also being evaluated to manage COVID-19 [25], [26]. It is a promising approach that can add a further non-invasive detection of the lung symptom evolution during treatment or home monitoring. Currently, it is still not mature enough for immediate use and calls for future improvements. Readers are referred to Section 5 for more details.

One topic not addressed in this summary is the use of audio-based recordings of speech, coughing, and breathing as an early sign of presymptomatic and asymptomatic cases and for tracking progression through symptomatic stages in the home and clinical environments. Recently, there has arisen a growing number of both academic and industry groups developing web-based apps for mobile devices (smartphones and tablets) with online voice, cough, and breathing-donation websites that have already collected large quantities of audio data together with health surveys. Though a promising approach – as shown, for example, in distinguishing COVID-19 coughs from coughs from other flu or flu-like symptoms [27] – feature extraction and machine learning algorithms are at an early stage of development and require validation for prediction accuracy and robustness. Other areas where audio data, specifically voice, can potentially contribute is in monitoring the recognized symptoms of cognitive and physical fatigue due to the neurophysiological effects of COVID-19. Although not yet applied to COVID-19, voice has been found useful in tracking cognitive load and fatigue under other conditions [28]; where subtle variations in pitch ("jitter") and amplitude ("shimmer") may be early warning signs. Further discussion of audio-based detection and tracking is provided in Section 5 ("Emerging mHealth Technologies for Monitoring and Prevention of COVID-19"). While this evaluation has focused on established COVID-19 symptoms and diagnostic indicators, there is growing evidence of other organ-level dysfunction and failure (e.g. central nervous, musculoskeletal, renal, and gastrointestinal system) such as neuromotor dysfunction, increased blood clotting, and loss of smell and taste [5], [29]–[32]. Detection of the early onset of subtle changes in sensory feedback and feedforward systems may benefit from technologies that monitor human motion and somatosensory systems, including neuromotor coordination (involved in speaking, breathing, and coughing) that may be obtained through audio measurements.

Specificity thresholds may need to be greater to catch early warning signs (e.g. at-home monitoring of healthcare workers) since discharged patients have already tested positive. Although a device may provide a clinical parameter close to the established gold standard measurement, the question arises whether a device signal can distinguish COVID-19-specific symptoms from other causes. For example, can the estimated heart rate variability or breathing patterns specific to COVID-19 be measured in the signals by the various devices, or would this fall under the purview of post-signal-processing that would not be practical to occur within a wearable device?

In addition to the benefits detailed above, home monitoring will provide benefits for all user groups through high-throughput medical screening capacity allowing longitudinal data aggregation in urban and rural populations. Multimodal signal processing and machine learning techniques within a device or across multiple devices should enable sensitive health assessments for subsequent decision making that extend beyond the current pandemic.

4.5 LIST OF EXAMINED TECHNOLOGIES

Manufacturer	Product Name
Apple	Apple Watch Series 5
Beddr	SleepTuner
Bell Lab	eSense
BioIntelliSense	BioSticker
BioVotion (now Biofourmis)	Everion
Current Health	Current Health
Emfit	Emfit
Empatica	EmbracePlus
Equivital	EQ02 LifeMonitor
Fitbit	Charge 4
Fitbit	Versa
Garmin	Femox
LifeSignals	BioSensor 2A
MC10	BioStamp nPoint
MindMics	Earbuds
Oura	Oura Ring
Polar	H10
ResApp Diagnostics	ResApp
ScaAI	SmartCardia
Sensium	Sensium
Sensogram Technologies	SensoRING
Sotera Wireless	ViSi
Spire Health	Health Tag
toSense	CoVA Monitoring System
VitalConnect	VitalPatch
WHOOP	WHOOP
Zephyr Technology	Zephyr

Table S4.3. Full list of technologies that were considered in this report.

REFERENCES

[1] W. J. Guan *et al.*, “Clinical Characteristics of Coronavirus Disease 2019 in China,” *N. Engl. J. Med.*, vol. 382, no. 18, pp 1708-1720, 2020
[2] F. Zhou *et al.*, “Clinical Course and Risk Factors for Mortality of Adult Inpatients with COVID-19 in Wuhan, China: A Retrospective Cohort Study,” *Lancet*, vol. 395, no. 10229, pp. 1054–1062, 2020
[3] C. Huang *et al.*, “Clinical Features of Patients Infected with 2019 Novel Coronavirus in Wuhan, China,” *Lancet*, vol. 395, no. 10223, pp. 497–506, Feb. 2020
[4] G. Maclaren, D. Fisher, and D. Brodie, “Preparing for the Most Critically Ill Patients with COVID-19: The Potential Role of Extracorporeal Membrane Oxygenation,” *JAMA*, 2020 [ePUB]
[5] L. Mao *et al.*, “Neurologic Manifestations of Hospitalized Patients with Coronavirus Disease 2019 in Wuhan, China,” *JAMA Neurol.*, 2020 [ePUB]
[6] J. Helms *et al.*, “Neurologic Features in Severe SARS-CoV-2 Infection,” *N. Engl. J. Med.*, 2020 [ePUB]
[7] B. Aylward and W. Liang, “Report of the WHO-China Joint Mission on Coronavirus Disease 2019 (COVID-19),” 2020. [Online]. Available: https://www.who.int/docs/default-source/coronaviruse/who-china-joint-mission-on-covid-19-final-report.pdf. [Accessed: 28-June-2020]
[8] Z. Wu and J. M. McGoogan, “Characteristics of and Important Lessons from the Coronavirus Disease 2019 (COVID-19) Outbreak in China: Summary of a Report of 72314 Cases from the Chinese Center for Disease Control and Prevention,” *JAMA*, 2020 [ePUB]
[9] World Healthy Organization, “Clinical Management of Severe Acute Respiratory Infection when COVID-19 Is Suspected,” *WHO*, 2020 [Online]. Available: https://www.who.int/publications-detail/clinical-management-of-severe-acute-respiratory-infection-when-novel-coronavirus-(ncov)-infection-is-suspected [Accessed: 28-June-2020]
[10] J. Schwartz, C. C. King, and M. Y. Yen, “Protecting Health Care Workers during the COVID-19 Coronavirus Outbreak -Lessons from Taiwan’s SARS response,” *Clin. Infect. Dis.*, vol. 2019, pp. 2019–2021, 2020.
[11] CDC, “Definitions of Signs, Symptoms, and Conditions of Ill Travelers,” [Online]. Available: https://www.cdc.gov/quarantine/maritime/definitions-signs-symptoms-conditions-ill-travelers.html. [Accessed: 28-June-2020].
[12] L. Milechin *et al.*, “Detecting Pathogen Exposure During the Non-Symptomatic Incubation Period Using Physiological Data,” *bioRxiv* 218818, 2017
[13] V. Jeyhani, S. Mahdiani, M. Peltokangas, and A. Vehkaoja, “Comparison of HRV Parameters Derived from Photoplethysmography and Electrocardiography Signals,” in *Proceedings of the Annual International Conference of the IEEE Engineering in Medicine and Biology Society*, 5952–5955, 2015
[14] P. Shi, S. Hu, and Y. Zhu, “A Preliminary Attempt to Understand Compatibility of Photoplethysmographic Pulse Rate Variability with Electrocardiogramic Heart Rate Variability,” *J. Med. Biol. Eng.*, vol. 28, no. 4, pp. 173–180, 2008
[15] B. Caulfield, B. Reginatto, and P. Slevin, “Not All Sensors Are Created Equal: A Framework for Evaluating Human Performance Measurement Technologies,” *NPJ Digit. Med.*, 2:7, 2019
[16] CDC, “People Who Are at Higher Risk for Severe Illness,” [Online]. Available: https://www.cdc.gov/coronavirus/2019-ncov/need-extra-precautions/people-at-higher-risk.html. [Accessed: 28-June-2020].
[17] S. Richardson *et al.*, “Presenting Characteristics, Comorbidities, and Outcomes Among 5700 Patients Hospitalized With COVID-19 in the New York City Area,” *JAMA*, 2020 [ePUB]
[18] “Sensium Disposable Digital Plaster Receives FDA Approval.” [Online]. Available: https://www.investegate.co.uk/ArticlePrint.aspx?id=201107110700181198K. [Accessed: 28-June-2020]
[19] “Sensium: Early Detection of Patient Deterioration.” [Online]. Available: https://www.sensium.co.uk/_assets/media/documents/brochures/brochure.pdf. [Accessed: 28-June-2020]
[20] “UCSF TemPredict Study.” [Online]. Available: https://ouraring.com/ucsf-tempredict-study. [Accessed: 28-June-2020]
[21] “WVU Rockefeller Neuroscience Institute and Oura Health Unveil Study to Predict the Outbreak of COVID-19 in Healthcare Professionals.” [Online]. Available: https://ouraring.com/wvu-rockefeller-neuroscience-institute-and-oura-health-unveil-study-to-predict-the-outbreak-of-covid-19-in-healthcare-professionals [Accessed: 28-June-2020]
[22] T. R. Ray *et al.*, “Bio-Integrated Wearable Systems: A Comprehensive Review,” *Chem. Rev.*, vol. 119, no. 8, pp. 5461–5533, Apr. 2019
[23] M. J. Buller *et al.*, “Estimation of Human Core Temperature from Sequential Heart Rate Observations,” *Physiol. Meas.*, vol. 34, no. 7, pp. 781–798, 2013
[24] J. Palmer, J. Williamson, B. Telfer, and W. Tharion, “Estimating Thermal-Work Strain and Physical Fatigue from a Wearable Physiological Status Monitor,” *J. Sci. Med. Sport*, vol. 20, p. S46, Nov. 2017
[25] “Call to Researchers to Help Use Bioimpedance to Manage COVID-19.” [Online]. Available: http://shimmersensing.com/about/news/call-for-researchers-to-help-use-bioimpedance-to-manage-covid-19 [Accessed: 28-June-2020]
[26] G. Gill, “COVID-19 : Wearable Sensors and Continuous Data To Improve Outcome,” [Online]. Available: https://www.shimmersensing.com/products/shimmer3-ebio-unit [Accessed: 28-June-2020]
[27] A. Imran *et al.*, “AI4COVID-19: AI Enabled Preliminary Diagnosis for COVID-19 from Cough Samples via an App,” pp. 1–12, 2020.
[28] T. Quatieri, T. Talkar, and J. Palmer, “Toward

Biomarkers of COVID-19 Based on Coordination of Speech-Production Subsystems," *IEEE Open Journal of Engineering in Medicine and Biolology*, 2020 [ePUB]]

[29] Y. C. Li, W. Z. Bai, and T. Hashikawa, "The Neuroinvasive Potential of SARS-CoV2 May Be at Least Partially Responsible for the Respiratory Failure of COVID-19 Patients," *J. Med. Virol.*, 2020 [ePUB]

[30] A. M. Baig, A. Khaleeq, U. Ali, and H. Syeda, "Evidence of the COVID-19 Virus Targeting the CNS: Tissue Distribution, Host-Virus Interaction, and Proposed Neurotropic Mechanisms," *ACS Chem. Neurosci.*, 2020

[31] S. Naicker, C. W. Yang, S. J. Hwang, B. C. Liu, J. H. Chen, and V. Jha, "The Novel Coronavirus 2019 epidemic and kidneys," *Kidney International*, vol. 97, no. 5. pp. 824–828, 2020

[32] H. Su *et al.*, "Renal histopathological analysis of 26 postmortem findings of patients with COVID-19 in China," *Kidney Int.*, 2020 [ePUB]

[33] "BioSticker System 510(k) FDA Clearance," *U.S. Food & Drug Administration*. 2019

[34] "VitalPatch 5D, 7D Biosensors 510(k) FDA Clearance," *U.S. Food & Drug Administration*. 2020

[35] N. Selvaraj, G. Nallathambi, R. Moghadam, and A. Aga, "Fully Disposable Wireless Patch Sensor for Continuous Remote Patient Monitoring," in *Proceedings of the Annual International Conference of the IEEE Engineering in Medicine and Biology Society*, pp. 1632–1635, 2018

[36] N. Selvaraj, G. Nallathambi, and P. Kettle, "A Novel Synthetic Simulation Platform for Validation of Breathing Rate Measurement," in *Proceedings of the Annual International Conference of the IEEE Engineering in Medicine and Biology Society*, pp. 1177–1180, 2018

[37] "COVID-19 Remote Health Monitoring in Hospitals and at Home." [Online]. Available: https://lifesignals.com/covid19/. [Accessed: 28-June-2020]

[38] A. C. W. Wong, D. McDonagh, O. Omeni, C. Nunn, M. Hernandez-Silveira, and A. J. Burdett, "Sensium: An ultra-low-power wireless body sensor network platform: Design & application challenges," in *Proceedings of the Annual International Conference of the IEEE Engineering in Medicine and Biology Society*, pp. 6576–6579, 2009

[39] "SensoRING SR200." [Online]. Available: https://www.sensogram.com/product-page/sensoring-sr200. [Accessed: 28-June-2020]

[40] "Current Wearable Health Monitoring System 510(k) FDA Clearance," *U.S. Food & Drug Administration*. 2019.

[41] T. Wyss *et al.*, "The Comfort, Acceptability and Accuracy of Energy Expenditure Estimation from Wearable Ambulatory Physical Activity Monitoring Systems in Soldiers," *J. Sci. Med. Sport*, vol. 20, pp. S133–S134, Nov. 2017

[42] M. Pavic, V. Klaas, G. Theile, J. Kraft, G. Tröster, and M. Guckenberger, "Feasibility and Usability Aspects of Continuous Remote Monitoring of Health Status in Palliative Cancer Patients Using Wearables," *Oncology*, vol. 98, no. 6, pp. 1–10, Jul. 2019

[43] S. K. Simblett *et al.*, "Patients' Experience of Wearing Multimodal Sensor Devices Intended to Detect Epileptic Seizures: A Qualitative Analysis," *Epilepsy Behav.*, vol. 102, p. 106717, Jan. 2020

[44] B. Bent, B. A. Goldstein, W. A. Kibbe, and J. P. Dunn, "Investigating Sources of Inaccuracy in Wearable Optical Heart Rate Sensors," *NPJ Digit. Med.*, 3:18, 2020

[45] "Everion data quality – Biovotion AG," *Biovotion*. [Online]. Available: https://biovotion.zendesk.com/hc/en-us/articles/212369329-Everion-data-quality-. [Accessed: 28-June-2020]

[46] L. Barrios, P. Oldrati, S. Santini, and A. Lutterotti, "Evaluating the Accuracy of Heart Rate Sensors Based on Photoplethysmography for in-the-wild Analysis," *ACM Int. Conf. Proceeding Ser.*, pp. 251-261, 2019

[47] L. Barrios, P. Oldrati, S. Santini, and A. Lutterotti, "Recognizing Digital Biomarkers for Fatigue Assessment in Patients with Multiple Sclerosis," in *Proceedings of the 12th EAI International Conference on Pervasive Computing Technologies for Healthcare*, 2018

[48] "Equivital Vital Signs Physiological Monitor EQO2 510(k) FDA Clearance," *U.S. Food & Drug Administration*. 2011

[49] A. A. Akintola, V. van de Pol, D. Bimmel, A. C. Maan, and D. van Heemst, "Comparative Analysis of the Equivital EQ02 Lifemonitor with Holter Ambulatory ECG Device for Continuous Measurement of ECG, Heart Rate, and Heart Rate Variability: A Validation Study for Precision and Accuracy," *Front. Physiol.*, vol. 7, pp. 1–14, 2016

[50] "BioHarness 510(k) FDA Clearance," *U.S. Food & Drug Administration*. 2012.

[51] G. Nazari and J. C. MacDermid, "Reliability of Zephyr BioHarness Respiratory Rate at Rest, During the Modified Canadian Aerobic Fitness Test and Recovery," *J. Strength Cond. Res.*, vol. 34, no. 1, pp. 264–269, Jan. 2020

[52] G. Nazari and J. C. MacDermid, "Minimal Detectable Change Thresholds and Responsiveness of Zephyr Bioharness and Fitbit Charge Devices," *J. strength Cond. Res.*, vol. 34, no. 1, pp. 257–263, 2020

[53] G. Nazari, J. C. MacDermid, K. E. Sinden, J. Richardson, and A. Tang, "Reliability of Zephyr Bioharness and Fitbit Charge Measures of Heart Rate and Activity at Rest, During the Modified Canadian Aerobic Fitness Test, and Recovery," *J. Strength Cond. Res.*, vol. 33, no. 2, pp. 559–571, 2019

Emerging mHealth Technologies for Monitoring and Prevention of COVID-19

5.1 Introduction

Mobile health (mHealth) technologies hold enormous promise to help combat the COVID-19 pandemic, which has already had catastrophic ramifications for healthcare systems around the world [1]. Through promoting telehealth consultations and by enabling unobtrusive long-term monitoring of COVID-19-related symptoms through remote sensing, mHealth technologies may help reduce unnecessary exposure of individuals to the virus during visits to healthcare facilities. Such technology could lead to means of early detection of symptoms, streamline the triage process, allow preventative monitoring of individuals at risk of exacerbation, prevent re-hospitalization, and test the efficacy of treatments. Ultimately these systems may be central to addressing the important societal challenges manifested by the COVID-19 pandemic.

Remote monitoring of COVID-19 requires inclusion of biophysical, biochemical, and vital sign metrics to enable constant surveillance of respiratory, neurological, cardiovascular, and mental health [2]–[5]. Although there do exist health tracking strategies and sensing platforms which offer important insights, they unfortunately cannot provide fine-grained, multi-modal and objective analysis of COVID-19 patients while also limiting patient contact and virus transmission [6], [7].

Prior to the pandemic, smartphones, wrist-based health trackers, and wearable patches cleared by the Food and Drug Administration (FDA) were promising exemplars of connected mHealth technologies that had begun to enable objective, sensitive, long-term monitoring of vital signs [6]. However, these approaches cannot capture the heterogeneity of affected body functions, as they often lack multi-modal biosensing capabilities and are limited to a few body locations.

Due to these shortcomings, there is an urgent need for novel mHealth solutions that provide complementary capabilities in screening, monitoring, and prevention of COVID-19. Research efforts are underway across academia, industry, and clinical settings to employ heterogeneous classes of technologies (ranging from wearables to advanced image processing and robotics), which have been largely unexplored but are expected to be quite relevant to understanding the onset of COVID-19 and its symptoms [8]–[13]. Although emerging mHealth research spans a wide range of technologies with varying readiness levels, many existing solutions are poised to directly address the challenges posed by COVID-19.

The main objective of this section is to highlight these emerging mHealth technologies. In the following, the range of these technologies are presented, and the key advantages and limitations of each solution are discussed in light of the challenges facing COVID-19 patients.

5.2 Emerging mHealth and Digital Technologies

The emerging mHealth devices and systems herein discussed (Table S5.1) were identified through scientific literature, public media reports, and company press releases highlighting pivots to combat COVID-19. The approaches were grouped into five distinct categories: 1) contactless sensing of physiological information; 2) wearable biosensors; 3) audio-based sensing of lung function and spirometry; 4) text-based sensing of mental health; and 5) robotic approaches. While many of these approaches leverage recent advances in machine learning and artificial intelligence (AI) for a meaningful processing of the recorded sensor data, this report will instead focus on the implementation of the sensing technologies.

5.2.1 Contactless Sensing of Physiological Data

Contactless sensing of human physiology provides a compelling opportunity to monitor critical COVID-19-related vital signs and symptoms with minimal user engagement in and outside the hospital setting. For example, using specific WiFi routers, sensors utilizing radio waves promise to accurately and

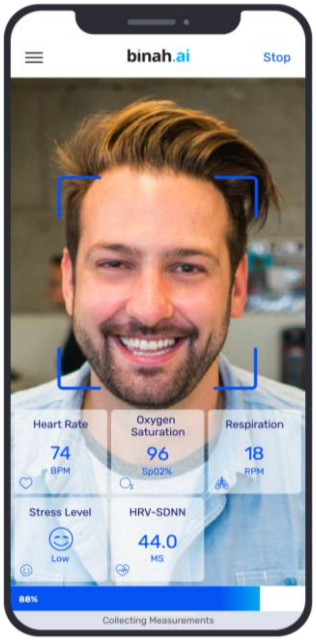

Fig. S5.1. Contactless-sensing of vital signs using digital cameras. Advanced signal processing and AI algorithms allow extracting vital signs from face-based video data collected with digital cameras. Such approaches promise a fine-grained and large-scale remote monitoring of potential COVID-19 symptoms. Courtesy of binah.ai, Tel Aviv, Isreal.

Technology type	Sensing modality	Provided information	Target application	Technology readiness
**A. Contactless sensing of physiological information**				
**WiFi routers**	Radio waves	Respiration rate, heart rate	Monitoring at home	Commercialization ongoing, pilot tests for COVID-19
**Instrumented mattresses**	Piezoelectric sensor	Respiration rate, heart rate	Monitoring in hospitals	Commercially available and in use for COVID-19, FDA approved
**Face videos**	Digital cameras	Respiratory rate, heart rate	Monitoring in hospitals and daily life	Commercialization ongoing, pilot tests for COVID-19
**B. Wearable biosensing systems**				
**Garment-based sensing**	Inertial and temperature sensors, inductance plethysmography	Heart rate, respiratory rate, skin temperature, estimated body core temperature, body motion	Monitoring in daily life	Commercialization ongoing, pilot tests for COVID-19
**Adhesive sensing patches**	Inertial and temperature sensors, mechanoacoustic sensing, bioimpedance	Respiratory rate, heart rate, skin temperature, blood pressure, swallowing, vocal fold vibration, and seismocardiograms	Monitoring in hospitals and daily life	Commercialization ongoing, pilot tests for COVID-19, pending FDA approval
**Instrumented protective personal equipment**	Face mask with sweat sensor	Body temperature	Monitoring of healthcare workers in contact with COVID-19	Commercialization ongoing, pilot tests for COVID-19, pending FDA approval
**C. Audio-based sensing and spirometry**				
**Smartphone apps**	Audio signals	Breath, cough, and speech characteristics	Monitoring in daily life	Collection of large-scale crowd-sourced public COVID-19 databases ongoing.
**Handheld spirometers**	Air flow and volume	Characterization of lung function (e.g., capacity, tidal volume)	Monitoring at home	Commercially available, pilot tests for COVID-19, FDA approved
**D. Text-based sensing of mental health**				
**Social media posts**	Text-based analysis	Mental health status, symptom self-reported	Monitoring at home	Research projects
**E. Robotic approaches**				
**Logistic and cleaning robots**	Indoor localization, object recognition, germicidal irradiation	Supply availability and locations covered	Supply delivery and surface disinfection	Commercially available
**Surgical robots**	Ultrasound imaging and miniaturized robotics	Venous blood samples	Testing for antibodies	Research projects
**Telepresence and social robots**	Indoor localization, object and face recognition	Video broadcast	Advanced communication and monitoring	Commercially available

Table S5.1. Overview of emerging mHealth and alternative technologies for monitoring and prevention in the COVID-19 pandemic.

passively monitor respiration and heart rate while the subject is in static conditions, such as while sleeping [8], [9]. Such systems could be deployed into homes of healthcare practitioners or individuals with a COVID-19 diagnosis, allowing fine-grained remote monitoring of vital signs in these at-risk populations. Initial commercial testing of such systems with COVID-19 patients are ongoing (Emerald Innovations Inc., MA, USA).

Another class of sensor technology exploit piezoelectric elements embedded into mattresses to passively track respiration and heart rate signals in clinical and home bedding. These approaches are commercially available (EarlySense, Ramat Gan, Israel) and have been extensively validated in high-risk surgical individuals [10], [11].

Non-contact video-based recordings of individuals' faces with digital cameras (Fig. S5.1) allow, through the use of

advanced signal processing algorithms and AI, a contactless and remote sensing of vital signs including heart rate, heart rate variability, and respiratory rate [12], [13]. Given that camera data can be easily obtained through closed-circuit television cameras and smartphones, these approaches promise a large-scale monitoring of vital signs in both preventive and acute scenarios in daily life and hospital environments. While the systems have been successfully validated from a technical perspective [12], [13], commercialization efforts are underway and initial tests with healthcare providers and COVID-19 patients are in progress (e.g., binah.ai, ISR; Carebook Technologies Inc., CAN) [14].

Given that COVID-19 can lead to pathological manifestations in lung tissue [5], [15], the automated processing of x-ray and ultrasound lung images using machine learning and AI techniques, such as deep neural networks, is receiving increased attention in the research community [16]–[18]. First results indicate that such abnormalities can be accurately detected, and commercialization efforts are ongoing to help improve the COVID-19 triaging process (Infervision Inc., Beijing, China).

5.2.2 Wearable Biosensing Systems

Skin mounted and garment-based wearables that are embedded in clothing or adhered to an individual's skin enable an intimate coupling of a sensing system with the human body. This allows for highly localized monitoring of vital signs and physiological signals, including respiratory rate, skin and core temperature, heart rate, and blood pressure. These vital signs can be used to inform on COVID-19 symptoms such as fever, cough, and dyspnea/tachypnea [19]. These sensing solutions often rely on inertial, temperature, and moisture sensors and transmit signals wirelessly to smartphones and cloud-based applications. As with contactless sensing, advanced classes of wearable sensors offer a non-obtrusive way to monitor critical vital signs and stress levels of individuals and healthcare workers during the COVID-19 pandemic.

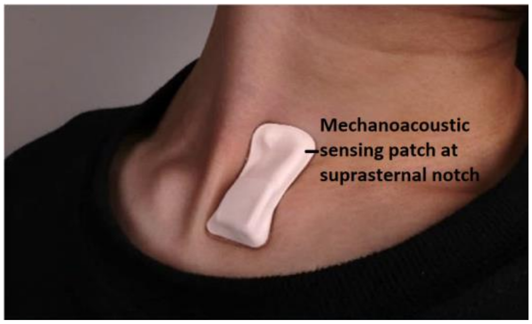

Fig. S5.2. Skin-interfaced wearable sensors. Skin-interfaced sensors allow unobtrusively monitoring physiological information. The Figure S5.hows a mechano-acoustic sensing device, allowing to measure coughing, swallowing, and respiration. Such wearable sensors are being applied to patients who have or are at risk of COVID-19. Courtesy of John A. Rogers, Northwestern University, IL, USA [20].

Skin-mounted solutions often come in the form of patches that can be attached, for example, to the chest (e.g., ScaAI patch, SmartCardia, Lausanne, Switzerland; LifeLens Technologies LLC., Warminster, PA.). These sensors can track symptoms of COVID-19 such as fever, cough, and dyspnea/tachypnea, and wirelessly transmit information to a cloud server for daily analysis and longitudinal assessment. Further, novel wearable mechano-acoustic sensors mounted at the suprasternal notch have been demonstrated to monitor swallowing, vocal fold vibration, and seismocardiograms (Fig. S5.2) [20], [21]. These wearable sensors have been tested in dysphasia patients, neo-natal pediatric intensive care units, and recently also to monitor cough features, sleep quality, and respiration and heart rate patterns of COVID-19 patient populations.

Skin-interfaced sensors also allow the characterization of thoracic bioimpedance, which is inversely proportional to the amount of fluid in the thoracic cavity, and has been used to track the progression of congestive heart failure [22], [23]. The Shimmer3 sensor (Shimmer Research, Dublin, Ireland) enables bioimpedance monitoring of the chest cavity, which could be used to assess lung function and when a COVID-19 patient requires aggressive treatment. These technologies are commercially available and are being tested in COVID-19 patient populations.

In addition, flexible biosensors promise to enable cuff-less blood pressure monitoring [24]. This is particularly useful due to reports of COVID-19 causing acute respiratory distress syndrome and significant drops in blood pressure. These sensors could support remote sensing of acute respiratory distress syndrome and reduce physical contact between healthcare workers and patients otherwise required for conventional cuff-based blood pressure measurements.

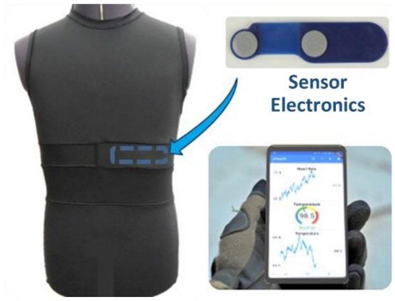

Fig. S5.3. Garment-based sensing of vital signs. Sensors embedded in garments (left and top right) can unobtrusively measure heart rate, respiratory rate, skin temperature, and body motion, and wirelessly transmit information to a smartphone (bottom right) therefore enabling a fine-grained monitoring of individuals affected with or at risk of COVID-19. Courtesy of Cornerstone Research Group, In, OH, USA.

Garment-based sensing systems provide intimate, multimodal, and highly distributed sensing. Different implementations of garment—based sensing systems exist. For example, some focus on monitoring changes in respiration and activity to infer stress levels (Fig. S5.3, Cornerstone Research Group Inc, OH), or tracking tidal volume, respiration rate, and core body temperature (Human Systems Integration Inc., East Wapole, MA). Further such approaches can also help provide multiple biomechanical insights that are not possible using discrete sensors, such as using a sensor mesh for detection of movement and joint biomechanics (Formsense, San Diego, CA). All of these technologies can support additional sensors for further physiological status monitoring.

In the present scenario, personal protective equipment, particularly respirators, have become a vital resource for protecting healthcare workers and individuals from the further

spread of coronavirus. The N95 respirators provide another wearable substrate for embedding biosensors that could monitor respiratory signals and, in turn, send alerts when the respirators need to be changed. An instrumented N95 respirator with onboard sensors and wireless connectivity is being developed for healthcare workers treating COVID-19 patients (Fig. S5.4, Epicore Biosystems, Inc., Cambridge, MA).

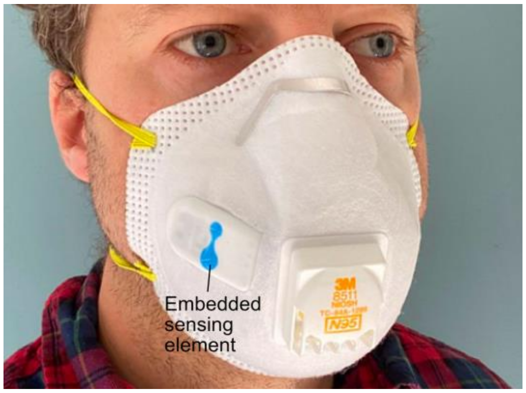

Fig. S5.4. Personal protective equipment with embedded sensing capabilities. These approaches can, for example, include sweat sensors integrated into facemasks that might support an early detection of fever for healthcare workers in contact with potential COVID-19 patients. Courtesy of Epicore Biosystems, Inc., MA, USA.

5.2.3 Audio-based Sensing of Lung Function and Spirometry

Audio-based recordings and signal processing of speech, coughing, and breathing patterns could provide an early warning detection system for onset and progression of COVID-19 symptoms in home or clinical environments. One of the core symptoms of COVID-19 is abnormal breathing characteristics caused by a specific dysfunction of the lower respiratory tract, which is expected to also affect speech production. Another important motivation for the use of audio signals is the growing evidence of neurological deficits and neuromuscular impairments that are likely present in COVID-19 patients [4], [25]. The virus may affect the finely coordinated respiratory, articulatory, and prosodic systems required for natural breathing and fluent speech. Finally, the observed characteristics of dry coughing associated with COVID-19 could be analyzed and may reflect unique changes in both physiological and neurological function. The disruptions in the natural patterns of breathing, coughing and speaking, thus hold promise for the discovery of new biomarkers, derived from acoustic measures, relevant to COVID-19 detection and progression [26].

There are a growing number of both academic and industrial groups developing web-based apps for mobile devices (smartphones and tablets) with online voice-donation sites that have collected large quantities of audio data together with health surveys (e.g., Voca.ai, Vocalis Health, University of Cambridge, Breath for Science, CoughResearch.ai, AI4COVID-19 [27]). These approaches have focused on one or more of the vocal expressions of breathing, coughing or speaking, and have led to web-based apps and protocols to collect participant audio (e.g., “cough twice and repeat”, “recite the alphabet”, “inhale and exhale three times”). Through the use of supervised machine learning, initial promising results indicate high accuracy in discriminating regular coughs from those of COVID-19-patients [27].

In addition to audio-based approaches, spirometers might also be applicable to the COVID-19 pandemic. Traditionally, these approaches serve as screening tools for chronic respiratory diseases, such as chronic obstructive pulmonary disease and asthma [28]. For patients with these illnesses, there has been a shift towards continuous monitoring of lung health using Bluetooth-connected spirometers (e.g. MIR Medical International Research, Rome, Italy). The dramatic impact of COVID-19 on the respiratory system, has spurred efforts currently underway to deploy spirometers at home and in hospital settings for the continuous tracking of lung health (NuvoAir, AB., Stockholm, Sweden).

5.2.4 Text-based Sensing of Mental Health

The COVID-19 pandemic and quarantine has led to a precipitous rise in mood disorders including major depressive disorder and generalized anxiety disorder [2]. Healthcare workers, in particular, have seen an increase in depression, anxiety, and general distress symptoms [29]. This situation presents unique challenges for mental health professionals to quantify and qualify the scope of mental health deterioration in individuals. Text-based analysis of posts from popular social media sites such as Reddit and Twitter may provide clinicians with important metrics that can reveal how individuals are responding to stress, and monitor how these individuals change their daily behaviors as the situation worsens. Researchers have been able to classify posts in social media websites based on self-reported diagnoses [30], [31]. Analysis of common themes and classifications of posts during the pandemic could help clinicians anticipate the prevalence of mental health disorders among the public, and therefore inform the types of resources that may be needed to address and monitor mental health challenges during this time. In addition to monitoring on a larger scale, these algorithms could be embedded in phone software applications. The software application could be trained to scan text messages for signs of depression and stress, which in turn could alert clinicians about specific patient needs.

5.2.5 Robotic Approaches

Robotic approaches (i.e. intelligent mechatronic systems including both sensing and actuation units) have potential to address challenges associated with the COVID-19 pandemic by reducing physical interactions in hospitals (navigation robots), advancing diagnosis of COVID-19 (surgical robots), and improving social care and communication (telepresence and social robots) [32].

Semi-autonomous robotic systems that are able to disinfect surfaces through ultraviolet germicidal irradiation and navigate within hospitals in an intelligent manner could reduce the danger of virus transmission and provide a cost-efficient, fast, and safe means of disinfection (Fig. S5.5) [32]–[35]. Such strategies have been commercialized (e.g. UVD Robots, Odense, Denmark; Xenex Disinfection Services, San Antonio, USA) and well-integrated into clinical care. Furthermore, recent reports indicate that these technologies could also kill the

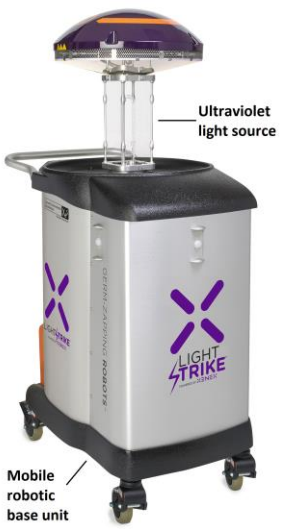

Fig. S5.5. Ultraviolet germicidal irradiation system. Mobile systems with ultraviolet light sources are used to semi-autonomously disinfect areas in hospitals, thereby reducing the risk of viral transmission. Courtesy of Xenex Disinfection Services, San Antonio, USA.

COVID-19 virus [36]. Similarly, robots that semi-autonomously distribute supplies, such as medications, laboratory specimens, meals, and trash, within hospitals could limit physical contact and are already integrated in a few healthcare environments (e.g. by ST Engineering Aethon, Inc., Pittsburgh, USA).

Robotic systems have also been shown to draw venous blood for laboratory tests, which is a necessity when testing for COVID-19 antibodies [32], [37]. In particular, the combination of ultrasound imaging and miniaturized robotics could enable safe draw of venous blood samples. Further advances are required to transfer these technologies from research environments into clinical care [37].

Lastly, telepresence and social robots are expected to improve communication with individuals in isolation, while simultaneously reducing the need for physical contact with health care workers [24], [25]. Such approaches are already commercially available in some nursing homes and hospitals (e.g. by Double Robotics, Burlingame, USA; Ava Robotics, Cambridge, USA).

5.3 Discussion

Emerging mHealth technologies offer compelling routes for combating the health-related challenges of the COVID-19 pandemic. Within this report, a heterogeneous landscape of promising technologies was identified and grouped into approaches focusing on contactless sensing, wearable biosensing systems, audio-based sensing and spirometry, text-based sensing, and robotics. From the available data, it is evident that no single type of emerging technology is sufficient to address all of the challenges the medical community faces during this pandemic. Instead, it is clear that the type of technology should be carefully chosen based on its specific intended application. For the monitoring of important physiological functions in the COVID-19 context, a multi-modal approach fusing physiological data obtained from a variety of sensing approaches appears to be the most promising as redundancy might help to address the often still limited robustness of technical solutions. In addition, further research is required to not only advance technical validation, but to also guarantee the usability of the solutions and to ensure that they can be integrated in existing IT ecosystems of healthcare solutions.

5.3.1 Contactless Sensing of Physiological Information

Contactless sensing technologies hold great promise for supporting the healthcare system during the COVID-19 pandemic. Examples of a few contactless technologies are provided below. It is beyond the scope of this section of the Supplementary Materials to provide a comprehensive review of the abundant literature with focus on this research area. The material herein presented is meant solely to bring to the readers' attention the relevance of advances in this research area to the clinical management of patients with COVID-19.

While using WiFi routers and radio waves for monitoring individuals at home is an innovative and promising concept [8], [9], such approaches require dedicated hardware, thereby limiting their scalability, and further research and commercialization efforts are required to ensure the robustness and usability of the technology.

Similarly, the machine learning-based analysis of X-ray and ultrasound lung images promises a rapid and automated diagnosis and assessment, but research efforts are still in an early phase, with most publications only being available on pre-print servers without being peer-reviewed yet [16]–[18].

The extraction of heart and respiratory rate from facial video data allows one to monitor vital signs. Given that such approaches only require video data recorded using smartphones or surveillance cameras, they have high scalability and might enable tracking of many individuals [12], [13]. The first integration efforts of such technologies into hospitals have been made, but further technology and product maturation seems necessary to unfold their full potential.

Lastly, there has been a great deal of research work in the area of contactless sensing technology motivated by commercial applications such as those pursued by the automotive industry [38]. Measurement approaches developed in the context of several commercial applications as well as the medical field have led to a variety of contactless systems for physiological monitoring. For instance, instrumented mattresses are already well integrated in clinical care and can reduce the length of hospitalization and days in the intensive care unit [11], potentially also in COVID-19 patients.

5.3.2 Wearable Biosensing Systems

Adhesive and garment-based wearables may prove to be practical, passive monitoring systems, providing alerts for patient deterioration or healthcare worker stress. They have also been shown to be reliable and robust, and less susceptible to motion-based artifacts as compared to traditional, wrist-worn sensors. There are, in addition, FDA 510(k) cleared adhesive sensors that are currently being pilot tested with COVID-19

patients. Though prior attempts to integrate adhesive and garment-based wearables have fallen short, technological breakthroughs in material science and microelectromechanical sensors, as well as efficient data handling techniques, have accelerated the development and reduced the cost of generating these sensors. However, in employing these wearables in the COVID-19 scenario, it is important to keep in mind that many of these sensors are still in the research and development stage. It will be important to assess the alerts that are being transmitted to healthcare professionals to prevent alarm fatigue, and to further understand how the vital signs being collected can provide an accurate and interpretable picture of patient and healthcare worker health.

One key technological gap lies in the development of biochemical wearable sensors as a way to characterize the underpinning metabolic activity of the body. The onset of dehydration, elevated stress biomarkers, and increasing sweat loss are critical biomarkers, which to date, have been difficult to characterize using conventional wearable technologies. However, novel technologies are emerging that might enable the assessment of these biochemical markers for dehydration, night sweating, and cytokine storms associated with COVID-19 [39], [40].

5.3.3 Audio-based Sensing of Lung Function and Spirometry

Audio sensing lends itself to unobtrusive widespread use through mobile devices. Thus, the approach provides a key capability for scalable, longitudinal studies that seek to capture human behavior dynamics in naturalistic environments for early warning and tracking of COVID-19 [26]. Though a promising direction, feature extraction and machine learning algorithms are in an early stage of development and require further maturation to ensure accuracy and robustness [27]. Further, it is essential to address potential confounders, such as different recording environments and channels, unbalanced data quantities when training machine learning models, and changes in underlying vocal status from pre-COVID-19 exposure to post-COVID-19 diagnosis. Finally, it is important to understand the specificity of audio-based biomarkers. For example, such markers must be able to differentiate COVID-19 from the typical flu and flu-like conditions resulting in various forms of inflammation.

While spirometry performed under guidance of healthcare practitioners in the hospital provides accurate and holistic information about lung function, its unsupervised application in the home environment with portable devices relies on the correct use of the technology, leading to varying levels of data quality [41], [42].

5.3.4 Text-based Sensing of Mental Health

Monitoring mental health status from text has been promising in research, as it allows for a large-scale collection of anonymized data, which has allowed researchers to garner insights into popular topics covered in subgroups of mental health diagnoses [43]. However, its application to mental health practice has not been validated. Diagnosis of individuals who post on social media are not confirmed, and therefore may skew the results. It will be important to also address issues with overlap between disorders or misdiagnoses. Finally, though the models derived from this approach may provide insight into mental health state during the pandemic, it will be important to work with clinicians to derive interpretable features, which can be useful for clinical decision making.

5.3.5 Robotic Approaches for Prevention

Robots which allow automated blood sampling are promising in the long-term [37], as substantial challenges regarding the robustness of the technology need to be addressed before a clinical integration is feasible.

Robotics supporting the supply chain management and logistics within a hospital and semi-autonomously disinfecting surfaces seem to be already well-established in multiple hospitals. Where they seem successful in reducing the number of unneeded physical contact between patients and staff. However, scientific studies providing evidence for these claims seem to be missing so far.

Similarly, telepresence robots have been well integrated into hospitals and nursing homes where they can help improve communication and potentially prevent social isolation [44]. Truly immersive environments that might also be able to render haptic interactions are, however, still under development [45].

5.3.6 Data Privacy, Ethical, and Legal Challenges

In employing any of these technologies, it is important to keep in mind potential issues with regards to protected health information [46][47]. As many of these technologies rely on Bluetooth Low Energy to transfer information, they need to be protected from hacking. With the emerging audio-based and camera-based vital sign estimation, which contain identifiable physiology, voice, and visual information, security and data privacy violations could lead to unauthorized access of personal information that could be misused. As a whole, remote collection of data, and storage of this data in the cloud, does lead to concerns about security, and therefore it will be important to ensure that measures are in place to handle sensitive user data and to comply with available legal regulations.

In addition to data security issues, there are also ethical concerns that need to be considered, as there is a chance that the envisioned rich and holistic behavioral picture of an individual intended for monitoring COVID-19 might be reused for other purposes, such as surveillance [48].

5.3.7 Limitations

The reported technologies were identified through a non-systematic search, thereby potentially not covering all COVID-19-related technological developments. Hence, future research is warranted to ensure the completeness of the reported activities.

5.4 Conclusions

In this section, a set of promising emerging technologies that address the challenges of the COVID-19 pandemic, especially

the remote and fine-grained sensing of biomarkers relevant to COVID-19, were highlighted. The available data suggests that a multi-modal technological approach is required to combat COVID-19 and that dedicated technologies are necessary for different application scenarios. The concise report of available technologies presented will act a steppingstone towards the development and clinical integration of innovative mHealth technologies for the management and mitigation of the COVID-19 pandemic and for health management as a whole.

REFERENCES

[1] W. J. McKibbin and R. Fernando, "The Global Macroeconomic Impacts of COVID-19: Seven Scenarios," 2020. [Online]. Available: https://www.brookings.edu/research/the-global-macroeconomic-impacts-of-covid-19-seven-scenarios/ [Accessed: 28-June-2020]

[2] S. K. Brooks *et al.*, "The Psychological Impact of Quarantine and How to Reduce It: Rapid Review of the Evidence," *The Lancet*, vol. 395, no. 10227, pp. 912-920, 2020

[3] Z. Wu and J. M. McGoogan, "Characteristics of and Important Lessons from the Coronavirus Disease 2019 (COVID-19) Outbreak in China: Summary of a Report of 72314 Cases from the Chinese Center for Disease Control and Prevention," *JAMA*, vol. 2019, 2020 [ePUB]

[4] L. Mao *et al.*, "Neurological Manifestations of Hospitalized Patients with COVID-19 in Wuhan, China: A Retrospective Case Series Study," *JAMA Neurol*, 2020 [ePUB]

[5] Z. Xu *et al.*, "Pathological Findings of COVID-19 Associated with Acute Respiratory Distress Syndrome," *Lancet Respir. Med.*, vol. 8, no. 4, pp. 420–422, 2020

[6] D. S. W. Ting, L. Carin, V. Dzau, and T. Y. Wong, "Digital Technology and COVID-19," *Nat. Med.*, vol. 26, no. 4, pp. 459–461, Apr. 2020

[7] S. Keesara, A. Jonas, and K. Schulman, "Covid-19 and Health Care's Digital Revolution," *N. Engl. J. Med.*, Apr. 2020 [ePUB]

[8] X. Liu and J. Cao, "Wi-Sleep : Contactless Sleep Monitoring via WiFi Signals," in *Proceedings - Real-Time Systems Symposium*, pp. 346-355, 2015

[9] F. Adib, H. Mao, Z. Kabelac, D. Katabi, and R. C. Miller, "Smart Homes that Monitor Breathing and Heart Rate," in *Proceedings of the 33rd Annual ACM Conference on Human Factors in Computing Systems - CHI '15*, pp. 837–846, 2015

[10] M. J. M. Breteler *et al.*, "Vital Signs Monitoring with Wearable Sensors in High-risk Surgical Patients: A Clinical Validation Study," *Anesthesiology*, vol. 132, no. 3, pp. 424–439, 2020

[11] H. Brown, J. Terrence, P. Vasquez, and D. W. Bates, "Continuous Monitoring in an Inpatient Medical-Surgical Unit: A Controlled Clinical Trial," *Am. J. Med.*, vol. 127, no. 3, pp. 226–232, 2014

[12] M. Z. Poh, D. J. McDuff, and R. W. Picard, "Advancements in Noncontact, Multiparameter Physiological Measurements Using a Webcam," *IEEE Trans. Biomed. Eng.*, vol. 58, no. 1, pp. 7–11, 2011

[13] M.-Z. Poh, D. J. McDuff, and R. W. Picard, "Non-Contact, Automated Cardiac Pulse Measurements Using Video Imaging and Blind Source Separation," *Opt. Express*, vol. 18, no. 10, p. 10762, 2010

[14] [Online]. Available: https://montrealgazette.com/news/local-news/jewish-general-set-to-roll-out-game-changing-app-in-coronavirus-battle

[15] A. Bernheim *et al.*, "Chest CT Findings in Coronavirus Disease-19 (COVID-19): Relationship to Duration of Infection," *Radiology*, vol. 295, no. 3, 200463, 2020

[16] L. Wang and A. Wong, "COVID-Net: A Tailored Deep Convolutional Neural Network Design for Detection of COVID-19 Cases from Chest Radiography Images," *arXiv*:2003.09871, 2020

[17] I. D. Apostolopoulos and T. A. Mpesiana, "Covid-19: Automatic Detection from X-ray Images Utilizing Transfer Learning with Convolutional Neural Networks," *Phys. Eng. Sci. Med.*, 2020 [ePUB]

[18] A. Narin, C. Kaya, and Z. Pamuk, "Automatic Detection of Coronavirus Disease (COVID-19) Using X-ray Images and Deep Convolutional Neural Networks," *Comput Biol Med*, 2020 [ePUB]

[19] G. Loke *et al.*, "Computing Fabrics," *Matter*, vol. 2, no. 4, pp. 786–788, 2020

[20] K. Lee *et al.*, "Mechano-Acoustic Sensing of Physiological Processes and Body Motions via a Soft Wireless Device Placed at the Suprasternal Notch," *Nat. Biomed. Eng.*, vol. 4, no. 2, pp. 148–158, Feb. 2020

[21] H. U. Chung *et al.*, "Skin-Interfaced Biosensors for Advanced Wireless Physiological Monitoring in Neonatal and Pediatric Intensive-Care Units," *Nat. Med.*, vol. 26, no. 3, pp. 418–429, 2020

[22] S. Weyer *et al.*, "Bioelectrical Impedance Spectroscopy as a Fluid Management System in Heart Failure," *Physiol. Meas.*, vol. 35, no. 6, pp. 917–930, 2014

[23] W. F. Peacock, N. M. Albert, R. D. White, and C. L. Emerman, "Bioimpedance Monitoring: Better than Chest X-ray for Predicting Abnormal Pulmonary Fluid?," *Congest. Hear. Fail.*, vol. 6, no. 2, pp. 86–89, 2000

[24] N. Luo *et al.*, "Flexible Piezoresistive Sensor Patch Enabling Ultralow Power Cuffless Blood Pressure Measurement," *Adv. Funct. Mater.*, vol. 26, no. 8, pp. 1178–1187, 2016

[25] J. Helms *et al.*, "Neurologic Features in Severe SARS-CoV-2 Infection," *N. Engl. J. Med.*, Apr. 2020 [ePUB]

[26] B. W. Schuller, D. M. Schuller, K. Qian, J. Liu, H. Zheng, and X. Li, "COVID-19 and Computer Audition: An Overview on What Speech & Sound Analysis Could Contribute in the SARS-CoV-2 Corona Crisis," *arXiv*:2003.11117, Mar. 2020

[27] A. Imran *et al.*, "AI4COVID-19: AI Enabled Preliminary Diagnosis for COVID-19 from Cough

Samples via an App," *arXiv*:2004.01275, 2020

[28] C. D. Wells and M. J. Joo, "COPD and Asthma: Diagnostic Accuracy Requires Spirometry," *J Fam Pract*, vol. 68, no. 2, pp. 76-81, 2019

[29] L. Duan and G. Zhu, "Psychological Interventions for People Affected by the COVID-19 Epidemic," *The Lancet Psychiatry*, vol. 7, no. 4. pp. 300–302, 2020

[30] J. H. Shen and F. Rudzicz, "Detecting Anxiety through Reddit," in *Proceedings of the Fourth Workshop on Computational Linguistics and Clinical Psychology - From Linguistic Signal to Clinical Reality*, pp. 58-65, 2017

[31] M. De Choudhury, M. Gamon, S. Counts, and E. Horvitz, "Predicting Depression via Social Media," in *Proceedings of the Seventh International AAAI Conference on Weblogs and Social Media*, pp. 128-137, 2013

[32] G.-Z. Yang *et al.*, "Combating COVID-19—The Role of Robotics in Managing Public Health and Infectious Diseases," *Sci. Robot.*, vol. 5, no. 40, pp. eabb5589–eabb5589, 2020

[33] C. R. Kovach, Y. Taneli, T. Neiman, E. M. Dyer, A. J. A. Arzaga, and S. T. Kelber, "Evaluation of an Ultraviolet Room Disinfection Protocol to Decrease Nursing Home Microbial Burden, Infection and Hospitalization Rates," *BMC Infect. Dis.*, vol. 17, no. 1, pp. 1–8, 2017

[34] N. G. Reed, "The History of Ultraviolet Germicidal Irradiation for Air Disinfection," *Public Health Rep.*, vol. 125, no. 1, pp. 15–27, 2010

[35] P. Sampathkumar *et al.*, "A Trial of Pulsed Xenon Ultraviolet Disinfection to Reduce Clostridioides Difficile Infection," *Am. J. Infect. Control*, vol. 47, no. 4, pp. 406–408, 2019

[36] Online]. Available: https://www.xenex.com/resources/news/xenex-lightstrike-robot-destroys-sars-cov-2-coronavirus-in-2-minutes/

[37] J. M. Leipheimer *et al.*, "First-in-Human Evaluation of a Hand-Held Automated Venipuncture Device for Rapid Venous Blood Draws," *Technology*, vol. 07 (3-4), pp. 98–107, 2019

[38] S. Leonhardt, L. Leicht, and D. Teichmann, "Unobtrusive Vital Sign Monitoring in Automotive Environments—A Review," *Sensors*, vol. 18, no. 9, p. 3080, Sep. 2018

[39] T. R. Ray *et al.*, "Bio-Integrated Wearable Systems: A Comprehensive Review," *Chem. Rev.*, vol. 119, no. 8, pp. 5461–5533, Apr. 2019

[40] J. Choi, R. Ghaffari, L. B. Baker, and J. A. Rogers, "Skin-Interfaced Systems for Sweat Collection and Analytics," *Sci. Adv.*, vol. 4, no. 2, pp. 1–10, 2018

[41] P. J. Chowienczyk, D. H. Parkin, C. P. Lawson, and G. M. Cochrane, "Do Asthmatic Patients Correctly Record Home Spirometry Measurements?," *BMJ*, vol. 309, no. 6969, p. 1618, 1994

[42] A. F. J. Brouwer, R. J. Roorda, and P. L. P. Brand, "Home Spirometry and Asthma Severity in Children," *Eur. Respir. J.*, vol. 28, no. 6, pp. 1131–1137, 2006

[43] A. Park, M. Conway, and A. T. Chen, "Examining Thematic Similarity, Difference, and Membership in Three Online Mental Health Communities from Reddit: A Text Mining and Visualization Approach," *Comput. Human Behav.*, vol. 78, pp. 98–112, 2018

[44] "Robotic Dog Helping Brigham and Women's Medical Workers Safely Talk to Patients with COVID-19." [Online]. Available: https://whdh.com/news/robotic-dog-helping-brigham-and-womens-medical-workers-safely-talk-to-patients-with-covid-19/ [Accessed: 28-June-2020]

[45] M. Reiner, "The Role of Haptics in Immersive Telecommunication Environments," *IEEE Trans. Circuits Syst. Video Technol.*, vol. 14, no. 3, pp. 392–401, 2004

[46] J. Abeler, M. Bäcker, U. Buermeyer, and H. Zillessen, "Covid-19 Contact Tracing and Data Protection Can Go Together.," *JMIR mHealth uHealth*, vol. 8, pp. 1–5, 2020

[47] T. H. McCoy and M. C. Hughes, "Preserving Patient Confidentiality as Data Grow," *JAMA Netw. Open*, vol. 1, no. 8, p. e186029, Dec. 2018

[48] U. Gasser, M. Ienca, J. Scheibner, J. Sleigh, and E. Vayena, "Digital Tools Against COVID-19 : Framing the Ethical Challenges and How to Address Them," *arXiv*:2004.10236, 2020

Technology-assisted Contact Tracing in the Hospital Setting

6.1 Introduction

The recent emergence of severe acute respiratory syndrome coronavirus 2 (SARS-CoV2) has led to widespread efforts to contain its spread. These include the implementation of isolation, quarantine, physical distancing, and personal protective equipment protocols, as outlined in Section 1 ("COVID-19 Related Clinical Issues, mHealth Technology Applications, and the Acceleration of the Digital Health Transformation"). This is especially important in hospitals where staff and healthcare providers come in close contact with dozens of people each day whose current infective status is unknown. Additionally, the hospital setting presents a unique challenge related to the shared use of common equipment and facilities. As such, the ability to monitor the location of people (i.e. personnel, patients, visitors) and equipment within the confines of the hospital would provide the opportunity to detect and act on potential exposures by implementing testing and containment measures in a timely manner.

Person-to-person contact is not the only means by which the virus can spread within a hospital. It has been shown that the virus can survive on surfaces for upwards of 72 hours [1][2]. Thus infected individuals may unknowingly contaminate surfaces and equipment which is shared by patients or healthcare staff [3][4]. Common areas in hospitals, such as sanitary facilities, are shared by patients, visitors and staff and can be a vector by which the virus can spread. Judicious disinfection regimens and hand hygiene may reduce this risk [5], but its effectiveness is highly dependent on the level of compliance. Even when compliance is high, contaminated areas or equipment could still be shared between scheduled disinfection procedures. As such, vigilant cleaning and hygiene and the other measures that have been put in place to curb internal infections (e.g. personal protective equipment, visitor restrictions) may not be sufficient.

To further compound these issues, viral load of symptomatic COVID-19 patients is at its peak when symptoms first appear [6]. It is estimated that infected individuals are likely shedding viral particles several days prior to developing symptoms. During this time, these individuals are likely to be out in the community, or in the hospital general ward, long before strict infection containment methods have been enacted. Reports from China indicate that healthcare personnel working in general wards contract COVID-19 four times more frequently than those in emergency rooms or ICUs, suggesting that asymptomatic transmission is a critical factor [7]. This highlights the need to rapidly identify any occurrences of critical events, including contact with infected people or potentially contaminated surfaces.

Contact tracing is an established and effective means of observing and controlling the spread of an infection within a population, especially in the face of a pandemic [8][9]. It consists of determining who has been in contact with whom and evaluating the risk of transmission. The process is iterative and requires compliance from all parties. Traditionally, contact tracing is performed manually, and involves successive calls to every identified contact who may have been exposed. Depending on the nature of each exposure, the number of calls needed to trace all parties can increase exponentially, making the process time consuming and potentially unsustainable.

Over the years, several technologies have been developed that can quickly and accurately track the location of people and assets within a hospital. These technologies have already been successfully implemented for equipment use and patient flow optimization. Combined with specially designed software, these same technologies can help automate the process of contact tracing, providing a list of potentially infected individuals and/or contaminated assets within a matter of minutes. These technologies have the potential to enable targeted early testing and implementation of infection control measures which could hold the key to success to getting ahead of the curve [9].

This section provides a review of available technologies that enable indoor location tracking. Existing solutions that deploy these technologies alongside contact tracing software are identified and a framework to assist evaluating potential solutions is presented. Preliminary recommendations are offered based on intended use cases. This work may enable individuals and institutions to make informed decisions when choosing to implement location-based contact tracing solutions in a hospital setting.

6.2 Materials and Methods

Although indoor real-time location systems have been in use for more than a decade, the recent pandemic has created new challenges and a pressing need to redefine system design and implementation requirements. This section was focused on three areas: 1) determination of current requirements; 2) survey of available technologies; and 3) survey of deployed products which satisfy the current requirements.

6.2.1 Evaluation of Requirements

Key stakeholders were consulted to determine a minimum set of desired specifications for a contact tracing product. Engineers and medical personnel were asked to define system requirements, such as minimum spatial and temporal resolution of location data; preferred spatial and temporal resolution; client-side device form factor, minimum device battery life;

preferred device battery life; and infrastructure power source. The importance of additional factors such as length of location history and level of contextual details surrounding contact events were also inquired towards. Additionally, administrative personnel were interviewed across various institutions that are implementing location systems and contact tracing products to determine factors beyond the technical specifications that were considered during implementation. An online questionnaire was created and presented to expert teams working on different aspects of the pandemic response. From this inquiry, 26 responses were received: 15 from healthcare professionals, 8 from engineers, and 3 from individuals with a background in both fields. These insights comprise the proposed framework for evaluating a particular product in the context of COVID-19.

6.2.2 Technology Survey

As an initial step, a web search was performed to identify technologies (e.g. Ultra-wide band, Bluetooth) that have been successfully implemented in either an indoor positioning system (IPS) or a real-time location system (RTLS). Search results were filtered to identify technologies that are commercially available. Those technologies that were still in the research stage of development at the time of the search were not considered. Once the available technologies were identified, additional searches were performed using the common name and acronym of the technology as well as the terms IPS and RTLS. Search results were combed to identify functional characteristics of each type of technology.

6.2.3 Product Survey

Once the technologies had been identified, products that integrated said technologies and were actively used in major healthcare facilities across the globe were highlighted. Products that had a strong presence in other industries (e.g. industrial or warehouse asset tracking) with available form factors appropriate for personnel tracking, had a high technology readiness, or offered a unique advantage, such as customizability or a unique combination of technologies were also considered. Identified products were selected and placed on a cloud based shared document to solicit feedback from healthcare practitioners and experts in the field.

Technical information about candidate systems was collected from publicly available sources, including product brochures, white papers, and web content. Data collected included the primary focus of the product; type of technology implemented; the need for additional infrastructure (e.g. network of receivers); client device form factor; spatial resolution; temporal resolution; availability of contact tracing modules; the types of data collected from the contact tracing modules; and electronic medical record integration. The decision to focus on these aspects of each product was determined based on consultation with other members of the innovation group which included engineers familiar with such technologies, frontline medical personnel, and administrative personnel. The primary focus of this Section was on systems that were already implemented in hospital settings due to the urgency to implement tested solutions; healthcare institutions that had already implemented these products were contacted to gather additional information and perspectives.

6.3 Results

Results from the survey of system requirements, technology, and product surveys are presented below.

6.3.1 Determination of Requirements

System requirements presented below consist of technical requirements, end-user requirements, and implementation requirements.

6.3.1.1 Technical requirements

Table S6.1 shows the core technical requirements a RTLS needs to be effective as part of a contact tracing solution.

Requirements for spatial resolution were strongly skewed to values below 2 m (6.6 ft). Values presented in Table S6.1 reflect the central tendency of the responses. The survey indicated that those with a technical background preferred finer granularity in location information than those with a medical background (1.0 m vs 1.6 m, respectively), but both agreed on the minimum requirement. Survey respondents indicated that associating a specific hospital room with location coordinates was important. In most cases, knowing solely the room without a precise location was considered sufficient.

Criteria	Minimum	Preferred
**Spatial Resolution**	2 m (6.6 ft)	1 m (3.3 ft)
**Temporal Resolution**	10 s	5 s
**Tag Battery Life**	1 week	1 month
**Receiver Battery Life**	6 months	> 1 year

Table S6.1. System technical and end-user requirements

The temporal resolution requirement, specifically the time interval between location measurements, was variable though shorter intervals were preferred. Most agreed that the precise time and date of each location measurement was important. Some survey participants noted that the interval between location updates would not need to be constant to be effective, as the smart application of additional sensing modalities could selectively update location based on movement characteristics.

6.3.1.2 End-user requirements

Most survey participants preferred a localization tag similar to standard ID badges. Small clip-on tags and the use of a smartphone as a tag were also popular form factors; whereas few identified a wristband or a watch as a desired option. When considering the battery life of tags, one month was widely indicated as the optimal time required between re-charging or replacement; though many were willing to accept a shorter battery life if necessary.

On the integration of receivers into hospital infrastructure, most responders preferred a solution that had a hardwired

option including Power-over-Ethernet and in-wall line power. Of those, most would choose a hybrid option that could leverage both constant power (hardwired) and battery power; but some preferred solutions that could be plugged-in to an existing wall outlet. None of those surveyed preferred battery-powered receivers as the primary option. In the event that battery-powered receivers were used, the battery life was expected to be at least six months, with longer durations preferred.

For contact tracing to be effective, most concurred that location information from the past two or more weeks should be considered. In addition to location information, more detailed information regarding identified contacts was considered beneficial. This included the amount of time a person spent in a particular location and the duration of a particular contact. Some deemed the ability to track compliance with routine infection control measures, such as room disinfection and hand hygiene, important. The ability of a contact tracing solution to integrate with the electronic medical record (EMR) was considered beneficial but not essential. The option to integrate information such as infection status, vital signs (e.g. respiratory rate), and individual risk factors, was of notable interest.

6.3.1.3 Implementation Requirements

Several important factors beyond the necessary technical and end-user specifications when deciding if and when to implement a tracing solution were identified. Specifically, a revision of institutional assets was considered necessary. Human assets were identified as an important component of the implementation process. The current organization of teams, the number of available staff, and their knowledge and experience with such technologies were important considerations. Additionally, the reception of staff and patients to these technologies was considered a critical factor as the effectiveness of these systems is largely dependent on compliance with their use.

Certain products offer additional integrations or functionality specific to certain network vendors (e.g. Cisco Systems, San Jose, CA), making the current network infrastructure an important factor to take into consideration. The physical infrastructure (e.g. the building layout and design) is also critical as it can determine the types of technology best suited to the environment. The institutions experienced with these systems encouraged others to evaluate existing workflows within their hospitals to determine if tracing systems can be readily integrated, or if new workflows must be implemented and enforced. Potential and intended use cases for these solutions should also be identified and clearly defined. Although this document focuses on the use of these products in the context of contact tracing, their utility has been proven in other areas such as equipment use and patient flow optimization. Hence, priorities with regard to hospital needs at a particular point in time (e.g. in response to a pandemic) were identified as important and need to be clearly defined as they may dictate the availability of funding.

In addition to institutional factors, a number of external factors can influence the decision to implement a location-based contact tracing solution. Among those surveyed, cost was identified as an important and variable factor that is highly dependent on the necessary system configuration per the above institutional factors. Depending on the complexity of the system, deployment may require extensive retrofitting or calibration procedures that can render sections of a hospital inoperable during installation. Scalability was also noted as an important factor as this can influence the length of time for deployment as well as the mode of purchase (i.e. partial vs complete). Technology readiness was a minor concern as most systems are already well-developed; and institutions with well-equipped teams may consider systems requiring additional in-house customization if they offer a distinct advantage.

6.3.2 Technology Survey

RTLS were first deployed and implemented in military and government installations as an extension of personal identification technologies for localization. Unlike GPS that provides global localization, but works poorly indoors, RTLS was developed for monitoring inside buildings or confined areas. Initial technological developments and commercialization of wireless technology facilitated the first commercial applications in the 1990s. Healthcare facilities were early adopters of RTLS technology [10]. Initially, healthcare implementations used infrared technology for room level localization inside hospitals. However, since infrared communication requires line-of-sight between transmitters and receivers, more recent implementations use wireless communication technology, commonly called Radio Frequency Identification (RFID), that can traverse the walls of a room. The International standard ISO/IEC 18000 (*Information technology - Radio frequency identification for item management*) defines wireless communication using different frequency ranges as parts of the main standard:

- Part 2: Below 135 KHz, Low data speed, 10cm range
- Part 3: 13.56 MHz, Low to moderate data speed, up to 1m range
- Part 4: 2.45 GHz, High data speed, several meters range
- Part 6: 860 – 960 MHz, Moderate/High data speed, 1-10m range
- Part 7: 433 MHz, Moderate data speed, up to 100m range

RFID-based RTLS use physical tags connected to people or objects [11]. Tags act as transmitters communicating with receivers (readers) in specific interrogation areas. Tags can be active (battery operated) or passive (battery-less). Typical configurations include an active reader/passive tag where the reader actively interrogates tags, a passive reader/active tag where the reader collects messages from tags, and active reader/active tag where the reader interrogates and wakes tags that continue active communication after that.

Widespread use and availability of WiFi Networks inspired a new generation of RTLS that use WiFi communication (ISO 18000-6c) [12]. However, since WiFi signal can penetrate walls, precise location within a room became a non-trivial problem. Another recently used approach is to estimate location

based on the strength of the received signal. This is particularly interesting for the standard wireless technologies, such as Bluetooth, Bluetooth Low Energy (BLE), ZigBee, and WiFi. Signal strength is highly non-linear in respect to distance and prevents precise determination of transmitter location. Recent machine learning methods allow much more precise determination of the transmitter location; but the system typically requires a custom reader infrastructure.

Ultra-wide Band (UWB) enables precise measurement of the signal's time-of-flight and fine resolution of the location. UWB radios communicate using narrow pulses with a wide frequency range (3.1-10.6 GHz). The system can compute the packet's time-of-flight to estimate the distance between the anchors, positioned in known coordinates, and tags positioned on the object/person. Combining multiple ranging and trilateration techniques, the system can estimate the location of the target with an accuracy between one and thirty centimeters. Emitted pulses can be received through walls.

UWB transmitters can be easily implemented and powered; however, readers require much higher complexity and power consumption. Although UWB systems provide the highest tracking accuracy, they are not widely used in clinical settings due to the required Line-Of-Sight (LOS) for maximum accuracy. The measures are less accurate when obstacles cover

Principle of operation	Advantages	Disadvantages
**Connectivity**		
**Infrared**	• Simple tags<br>• Low power consumption	• Room level location<br>• Line of sight necessary
**Wireless/RFID**	• Simple tags	• Room level location possible only with triangulation (multiple receivers or multiple antennas)
**Ultrasound**	• Precise distance measurement	• Scalability
**Time of Flight measurement**		
**UWB**	• Precise distance measurement<br>• Low complexity of transmitter only tags	• Complexity and power consumption
**Ultrasound**	• Precise distance measurement	• Distance to the closest object is measured only<br>• Processing complexity (multilateration)
**Phase measurement/ Angle estimation**		
**Bluetooth/BLE**	• Simple and inexpensive tags<br>• Low power consumption	• Complexity of receivers<br>• Infrastructure: the need for the dense network of receivers
**WiFi**	• Simple tags	• Power consumption of the transmitter<br>• Complexity of receivers<br>• Infrastructure: the need for the dense network of receivers
**Strength of the signal**		
**Bluetooth/BLE/ZigBee**	• Simple tags<br>• Low power consumption<br>• Low price	• Location resolution
**WiFi**	• Simple and inexpensive tags<br>• High data bandwidth<br>• Availability<br>• Existing infrastructure for some applications	• Location resolution<br>• New infrastructure with custom receivers for better resolution<br>• Bandwidth sharing with existing application might influence accuracy and interfere with existing systems/devices
**RFID**	• Simple tags<br>• Tags could be passive	• Range depends on frequency
**Custom wireless**	• Simple tags	• Dedicated receivers<br>• Infrastructure<br>• Interoperability
**Visual Methods**		
**Face/Object recognition**	• No personal tags<br>• Standard technology	• Face of users cannot be covered (e.g. use of masks)<br>• Need to register users /faces
**Barcode/QR code scan**	• Standard technology<br>• Inexpensive<br>• Battery-less personal tags	• Tags must be visible at all times
**Multimodal**		
	• Robust location assessment	• Complexity/price

Table S6.2. Technologies for real-time location systems

the view of the transmitter, which makes them more applicable in open space industrial environments where it is possible to position multiple transceivers and mitigate non-LOS effects. New more accurate RTLS are under development, such as millimeter-accurate systems-on-a-chip, that can be used for precise tracking of low-cost wearable sensors with minimal overheard. And with integration of UWB controllers in mobile phones and new infrastructure, secure and precise location tracking in hospitals will become increasingly easier to implement.

Recent development and massive deployment of small cameras with embedded processors have made it possible to read barcodes and QR codes, as well as perform real-time face recognition of registered users. However, barcodes and QR codes can be easily obstructed, preventing the automatic reading of the tags, and face masks complicate if not outright prevent facial recognition. Table S6.2 describes possible RTLS approaches, as well as advantages and disadvantages of the main RTLS technologies.

6.4 PRODUCT SURVEY

The list of systems presented in this section is not intended to be a complete survey of all available products and solutions. As several companies that manufactured systems for indoor location tracking were identified but failed to meet the criteria set in the survey. Of these, most were less developed solutions, which had designs that were not appropriate for tracking individuals due to a number of disqualifying variables (e.g. size and weight of asset tags, etc.), or had hardware limitations (e.g. limited number of tags supported) that would make the requisite scaling for larger institutions problematic.

The solutions presented in this section are examples selected from the larger list due to their completeness and ability to deploy quickly, which was of paramount importance at the time of writing. Table S6.3 shows a summary of identified contact tracing solutions.

It should be noted that specific product depictions,

Solution	Provider	Description
**A. Complete Solutions for Healthcare**		
AeroScout RTLS	Stanley Healthcare	AeroScout is a RTLS solution that combines Wi-Fi, low frequency RF, and ultrasound. This combination is capable of meeting most spatial resolution requirements. The system supports EMR integration, however, it is not equipped with a dedicated contact tracing module. Various installation options are available (hardwired, battery powered, hybrid).
CenTrak	CenTrak	CenTrak is a popular RTLS that leverages a variety of technologies including infrared, Wi-Fi, low frequency RF, UHF active RFID, Bluetooth Low Energy (BLE), and ultrasound. These combinations allow the system to satisfy a diverse range of use cases with distinct spatial resolution requirements. The system also supports EMR integration and is equipped with a contact tracing module. Various installation options are available (hardwired, battery powered, hybrid).
HID Beeks	HID Global	HID Beeks is a RTLS solution that exclusively uses BLE to provide location services. Spatial resolution is highly dependent on the number and location of BLE beacons. The system does not offer EMR integration, however, it provides access to software that enables contact tracing. Installation options are limited to beacons that must be plugged into an outlet.
Infinite Leap	Infinite Leap	Infinite Leap is a RTLS consultant and systems integrator. They offer a software product through sister company (prompt.health) that provides software that enables contact tracing.
Midmark RTLS	Midmark	Midmark RTLS is a RTLS solution that leverages a variety of technologies including infrared, WiFi, and UHF RF. This combination is adequate for a number of use cases. Precise location is enabled primarily by infrared and RF sensors located above target areas. Offers EMR integration and is equipped with a contact tracing module. Installation options include hardwired and battery powered modules.
Radianse	Radianse	Radianse is a RTLS solution that uses solely RFID for location services. Spatial resolution is dependent on the density of receivers. It does not offer EMR integration or a contact tracing module, though location of people or assets can be filtered through a dashboard to get similar information. Installation is primarily hardwired.
**B. Complete Solutions for Other Industries***		
Humatics	Humatics	Humatics is a RTLS system that leverages ultra-wide band for location services. It primarily serves warehouses for vehicle tracking, however, as devices become smaller, such solutions may become feasible for people tracking. Incorporates artificial intelligence software that can extract insights and could potentially be used for contact tracing in the future.
**C. Software Only Solutions***		
In Sites for Clinics	Infor	In Sites for Clinics is a software only solution that can integrate information from a variety of sources including RTLS, RFID systems, and BLE systems. It is capable of integrating with EMRs and has a contact tracing module available.
**D. Hardware Only Solutions***		
Estimote	Estimote	Estimote is a location and proximity solution that leverages ultra-wide band, BLE, and cellular technologies (LTE-M/NB-IoT) to provide location services. It is unique in that it is a fully customizable and programmable system with an API that enables integration with other BLE hardware. Because of this, it requires programming knowledge to deploy and manage. A contact tracing module is proposed, but details are not readily available.

*Selected examples for illustration purposes only.

Table S6.3. Examples of identified contact tracing solutions

illustrations or descriptions should not be considered endorsements, recommendations or specific criticisms on the part of the MGB network or its affiliated institutions or any of the Institutions that the authors of this report are affiliated with.

6.5 DISCUSSION

Many indoor location systems exist on the market, each with their own particulars of location methods and technologies. Those that offer the ability to perform contact tracing are all viable solutions, though further improvements are possible. The systems identified for use in the healthcare setting can provide real-time location of people and assets in the hospital and allow users to identify coincident events. Some allow specific tags (i.e. individuals, assets) to be flagged if they meet specified time and proximity criteria. In the survey of available technologies, no one system could integrate other factors deemed important, such as compliance with infection control measures and individual risk factors, to generate actionable insights. The integration of this information is currently a manual process that requires a user to obtain additional information from medical records and infer compliance information based on location information or other sources. With the evolution of machine learning and artificial intelligence, the prospect of integrating information from a variety of sources to analyze complex interactions automatically is growing into a viable and realistic option. In addition to expediting the contact tracing process and allowing for testing and isolation measures to be swiftly implemented, such methods could provide insights on the effectiveness of infection control measures, infection rates, and the factors that most influence the probability of infection based on available information.

Several factors that could pose potential barriers to implementation, including compliance and potential privacy concerns, were identified. Contact tracing solutions can only be effective if all individuals are using a tag, and access to additional contextual information can enhance the process. Although no consensus was found regarding how to address compliance issues, any policies instituted must be all-inclusive and mechanisms must be instituted to promote and enforce these measures.

Privacy was a major concern reported among those who partook in the questionnaire. Given the availability of precise staff locations, many raised concerns that such comprehensive monitoring and surveillance may cause undue stress on staff members which in turn may lead to compliance issues. Consequently, this feature is generally not implemented.

The current pandemic has created a new sense of urgency, and there is now a need to reevaluate policies with respect to privacy and system operations. For example, data retention policies that were previously limited to a matter of days (e.g. 72 hours) should be re-evaluated in the current context to allow for effective contact tracing. Privacy while performing contact tracing in the hospital can be preserved by adhering to the Health Insurance Portability and Accountability Act (HIPAA) regulations. Issues may arise when third parties are involved, especially if medical record integration is enabled. The decision to enable such integrations should be weighed carefully.

The identified solutions for the healthcare sector were considered complete and are already implemented in hospitals around the world. These solutions incorporate a mix of technologies and characteristics that allow these systems to adapt to the varied needs of healthcare facilities.

In the context of hospitals during the current pandemic, four example cases were considered, each with distinct needs: 1) facilities with no location tracking system; 2) facilities under construction; 3) facilities with an existing location tracking system; and 4) field hospitals.

Existing hospitals with no location tracking system in place face the unique challenge of quick deployment and the need to retrofit their facilities to accommodate a new system. In this context, a system that does not require modifications to the facility itself and can piggyback or interface with the existing communication infrastructure would offer a distinct benefit. For some institutions it may be preferable to opt for a system that can interface with existing communication infrastructure, but that requires minor modifications to physical infrastructure. Such systems should offer the possibility of converting to more robust installations in the future.

Hospitals in the process of being built have the flexibility to choose from a wide range of options. Such facilities can focus on designing a system that meets their projected needs and workflows and can immediately benefit from the advantages that a hardwired system offers. An important factor to consider, however, is future expandability.

On the other extreme are hospitals that already have a location tracking system in place. These institutions have already made a significant investment in their current system. It is important for these hospitals to evaluate their system against their immediate and future needs. It would be prudent for hospitals in this circumstance to expand the capabilities of their current system by acquiring additional infrastructure or software products capable of contact tracing.

Field hospitals have a unique set of needs driven by their inherent characteristics and use cases (see Section 2). These facilities are typically deployed in disaster scenarios and are single-level, high-density, volatile, and temporary. Additionally, they may not have the same robust communication or physical infrastructure as other facilities, limiting potential options. More traditional hardwired systems tend to have a high initial cost and are meant for permanent installations, making portable systems (minimal infrastructure) an attractive option. Several systems, such as UWB systems, have the capability to perform well in high-density and volatile field hospital setups. No complete solutions that meet the needs of such facilities, however, were identified in the survey of available options. A combination of an appropriate asset tracking system and a software only solution could be a potential short-term solution. An alternative does exist for institutions with teams capable of developing such systems, where they can opt for solutions that provide greater hardware flexibility but require significant programming and system design to implement successfully.

Emerging technologies that leverage hardware found in

ubiquitous devices (e.g. cell phones) could be used to expand and strengthen the capabilities of RTLS systems. Apple (Cupertino, CA) and Google (Mountain View, CA) have recently partnered to develop libraries to enable privacy-preserving contact tracing and facilitate exposure notification [13]. Although designed to be used at a larger community scale (for similar technologies, see Section 7 "Technology-Based Contact Tracing Solutions to Contain the Spread of COVID-19 in the Community"), the increased availability of WiFi and BLE enabled wireless access points (AP) within institutional communication infrastructure provides an opportunity to integrate information about the location of a user in the vicinity of an access point. Additionally, the availability of UWB controllers in smartphones, although not currently used for location tracking, offers the prospect of widespread adoption of more precise location technologies [14]. These functionalities are not currently widely available for use.

This section was intended to provide an overview of existing technologies and systems that could be implemented to perform contact tracing in a hospital setting. The information presented is not exhaustive and reflects the authors' best efforts to extract accurate information from publicly available sources and expert opinion. The effectiveness of such systems is highly dependent on system configuration and integration into hospital workflows. Currently, no existing implementations fully satisfy the needs for contact tracing of current or future pandemics. Future implementations should carefully consider specific use cases and institutional factors (e.g. infrastructure) during the design phase to ensure that the needs of contact tracing are met. Furthermore, the use of these systems to enable targeted early testing and implementation of strict infection control measures has not been tested. The authors intend to test this in a future pilot study to evaluate the feasibility and effectiveness of implementing location-based contact tracing.

6.6 CONCLUSIONS

Several healthcare-specific location-based tracking systems are available for deployment. Each system is unique and can be adapted to serve a range of potential use cases. All systems should meet the minimum criteria specified in this report to be considered as a viable solution. When choosing to implement a system, institution-specific factors and needs should be strongly considered. Successful implementation requires a detailed plan that includes design and workflow needs. Solutions that implement both location tracking and contact tracing are preferable for most. Emerging technologies that leverage hardware in ubiquitous devices (e.g. UWB controller in cell phones), implemented in wireless APs have the potential to provide added value to solutions by expanding capabilities; however, they are not yet available for integration. The use of location-based contact tracing solutions provides a ripe opportunity to strengthen efforts to stop the spread of SARS-CoV-2.

REFERENCES

[1] A. W. H. Chin *et al.*, "Stability of SARS-CoV-2 in Different Environmental Conditions," *The Lancet Microbe*, 2020 [ePUB]

[2] N. van Doremalen *et al.*, "Aerosol and Surface Stability of SARS-CoV-2 as Compared with SARS-CoV-1," *N. Engl. J. Med.*, vol. 382, no. 16, pp. 1564–1567, Apr. 2020

[3] G. Ye *et al.*, "Environmental Contamination of the SARS-CoV-2 in Healthcare Premises: An Urgent Call for Protection for Healthcare Workers," *medRxiv*, 2020.03.11.20034546, Jan. 2020

[4] S. W. X. Ong *et al.*, "Air, Surface Environmental, and Personal Protective Equipment Contamination by Severe Acute Respiratory Syndrome Coronavirus 2 (SARS-CoV-2) From a Symptomatic Patient," *JAMA*, vol. 323, no. 16, pp. 1610-1612, Mar. 2020

[5] J. Y. C. Lo, T. H. F. Tsang, Y.-H. Leung, E. Y. H. Yeung, T. Wu, and W. W. L. Lim, "Respiratory Infections during SARS Outbreak, Hong Kong, 2003," *Emerg. Infect. Dis.*, vol. 11, no. 11, pp. 1738–1741, Nov. 2005

[6] X. He *et al.*, "Temporal Dynamics in Viral Shedding and Transmissibility of COVID-19," *Nat. Med.*, vol. 26, no. 5, pp. 672-675, Apr. 2020

[7] D. Wang *et al.*, "Clinical Characteristics of 138 Hospitalized Patients With 2019 Novel Coronavirus–Infected Pneumonia in Wuhan, China," *JAMA*, vol. 323, no. 11, p. 1061, Mar. 2020

[8] K. T. D. Eames and M. J. Keeling, "Contact Tracing and Disease Control," *Proc. R. Soc. London. Ser. B Biol. Sci.*, vol. 270, no. 1533, pp. 2565–2571, Dec. 2003

[9] A. Wilder-Smith and D. O. Freedman, "Isolation, Quarantine, Social Distancing and Community Containment: Pivotal Role for Old-style Public Health Measures in the Novel Coronavirus (2019-nCoV) outbreak," *J. Travel Med.*, vol. 27, no. 2:taaa020 Mar. 2020

[10] C. E. Oude Weernink, E. Felix, P. J. E. M. Verkuijlen, A. T. M. Dierick-van Daele, J. K. Kazak, and J. van Hoof, "Real-time Location Systems in Nursing Homes: State of the Art and Future Applications," *J. Enabling Technol.*, vol. 12, no. 2, pp. 45–56, Jun. 2018

[11] B. Wang, M. Toobaei, R. Danskin, T. Ngarmnil, L. Pham, and H. Pham, "Evaluation of RFID and Wi-Fi Technologies for RTLS Applications in Healthcare Centers," in *2013 Proceedings of PICMET '13: Technology Management in the IT-Driven Services (PICMET)*, pp. 2690–2703, 2013

[12] D. A. Kirov, R. Passerone, and A. A. Ozhiganov, "A Methodology for Design Space Exploration of Real-time Location Systems," *Sci. Tech. J. Inf. Technol. Mech. Opt.*, vol. 15, no. 4, pp. 551–567, Jun. 2015

[13] "Privacy-Preserving Contact Tracing," 2020. [Online]. Available: https://www.apple.com/covid19/contacttracing [Accessed: 28-June-2020]

[14] "Apple U1 TMKA75 Ultra Wideband (UWB) Chip Analysis," 2020. [Online]. Available: https://www.techinsights.com/blog/apple-u1-tmka75-ultra-wideband-uwb-chip-analysis [Accessed: 28-June-2020]

Technology-based Contact Tracing Solutions for Containing the Spread of COVID-19 in the Community

7.1 INTRODUCTION

Contact tracing can be an effective approach to contain the spread of infectious diseases [1]. Plans are underway to develop a temporary workforce of manual contact tracers in several US states [2][3]. Manual contact tracing typically consists of asking a person with a confirmed diagnosis to recall all close contacts they might have had over the past two weeks. This approach is cumbersome, suffers from recall bias and is likely to have limited effectiveness for a highly contagious disease like COVID-19 [4].

Given the worldwide adoption of smartphones and other smart devices, devices which are often bundled with technologies such as Bluetooth, Global Positioning Systems (GPS) and a broad range of sensing modalities (e.g. accelerometer, gyroscope, magnetometer, barometric pressure sensors), opens the possibility for continuous, fine-grain monitoring of population location, proximity and behavior [5]. Countries like Singapore, Iceland and South Korea have already demonstrated that smartphone-based contact tracing can be an effective tool in managing the spread of COVID-19 [6][7]. The successes of these countries has inspired organizations around the world to further develop promising solutions. However, a careful assessment of this technology is required to understand their effectiveness, as well as the risks associated with collection and use of sensitive personal information [8]. To address this need, this section details a survey conducted of contact tracing solutions and the methodology developed to characterize and compare them and identify gaps that could be addressed to improve their effectiveness.

The gross steps involved in smartphone-based contact tracing are illustrated in Figure S7.1. Such contact tracing is based on using the location data of the user (derived using GPS or cellular network signals) or proximity (derived using Bluetooth) to detect contact between smartphone application (“app”) users [9]. When contact occurs between users of the app, phones will exchange randomly generated numbers with each other and store them locally (typically for 14 days).

Were a user diagnosed with a contracted disease (such as COVID-19), the anonymized data from their phone can be uploaded to a database (be it healthcare, governmental, or another institution) with the user’s permission. The data stored on the smartphone can then be checked to identify if other users came within a pre-defined proximity threshold of the infected individual over the stored period. The locally stored data of “contact” can then be uploaded to a designated database which can either de-anonymize the contact details (if approved to do so via a government initiative) or stay anonymous, and alert users who have been in contact with the COVID-19 positive patient about any risks they may have and the steps they may need to take (e.g. users may be directed to self-quarantine as a precaution depending on local public health policy).

Most smartphone apps have been designed to use Bluetooth to measure proximity which provides limited information. Additionally, in dense urban environments, Bluetooth connections may beget many false positives. As such, in order to improve sensitivity and specificity of smartphone-based contact tracing, utilizing additional data sources would be useful to derive a more granular understanding of mobility and behavior. For example, by using sensors like GPS, accelerometer and gyroscope, it would be possible to detect spatial and temporal context, including mode of transportation (e.g. on-foot, bike, bus, train or car), travel purpose and patterns of activity and behavior [10]. Sonar technology could also be used in this context, as recently proposed by NOVID [11]. Such data could be further augmented with information such as self-reported symptoms, any pre-existing conditions, notable medical history, and the person’s occupation; which would allow for better estimates of exposure risk and help decision-makers prioritize the allocation of limited resources, such as medical assistance or tests.

When capturing this type of information, it is vitally important to abide by national guidelines and other high standards of privacy to acquire and store the data in an anonymous and decentralized manner to reduce privacy concerns and minimize the risk of exposure or potential misuse of sensitive data or use blockchain-based methodologies instead of relying on a central entity that has the plan of record. Therefore, both users and decision-makers must carefully consider the best practice to minimize any potential risks to user privacy before recommending or using any smartphone-based contact tracing solution.

7.1.1 METHODOLOGY

To evaluate contact tracing in the community solutions, several characteristics were identified that elucidated the value they could deliver to users, the risks associated with that type of data, and how it might be used (Table S7.1). Based on a preliminary search of these solutions, we decided to include online surveys and data aggregators that could directly or

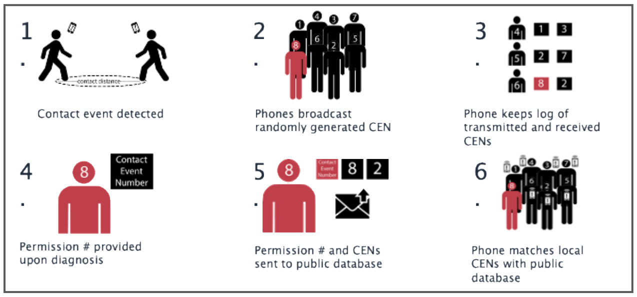

Fig. S7.1. An illustration of steps involved in smartphone-based contact tracing. CEN = Contact Event Number. system (Image reproduced with permission from COVID Watch [17])

Characteristic	Description
Solution Type	How is the solution delivered to the end user?<br><br>*Options:*<br>Smartphone App, Online Survey, Data Aggregator
OS Support	Which operating systems are supported by the solution? (limited to smartphone app solutions)<br><br>*Options:*<br>iOS, Android
Deployment Approach	How is the solution deployed?<br><br>*Options:*<br>Standalone = Solution can be deployed on its own<br>Enabling Technology = Solution can be deployed as a part of another solution
Data Collected	What type of data does the solution collect?<br><br>*Options:*<br>Age, Gender, Location, Proximity, Phone Number, IP Address, Contact Duration, Symptoms, Zip Code, Travel Mode, Trip Purpose, Covid Diagnosis, Travel History, Medical Conditions, Temperature, Covid Contact, Quarantine Status, Race, Ethnicity, Household Info, Health Insurance Coverage, Email Address, Case Statistics by Location, Age Group, Occupation, Smoking Status, Medications, Flu Vaccine Status, Population Mobility, Behavioral Insights, Contact Distance
Open Source	Is the solution open source or proprietary? (yes or no)
Privacy Preserving	Does the solution preserve privacy of the end user? (yes or no)
Location Tracking	Does the solution track the location of the end user? (yes or no)
Privacy Policy	What is the privacy policy of the solution? (URL, if available)
Status	What is the availability status of a solution?<br><br>*Options*:<br>Active = Solution is currently available<br>Development = Solution is under development<br>Concept = Solution is at a concept stage and development has not started yet
Geographical Availability	Where can the solution be deployed? (Country, Region or Worldwide)

Table S7.1. Characteristics that were used for assessing solutions for contact tracing in the community

indirectly support smartphone-based contact tracing. Data aggregators (services like Google Maps) keep a record of users' location histories. The evaluated solutions were reviewed based on publicly available information and, when possible, through hands-on testing. Where possible, some of the developers were interviewed to get a deeper understanding of the solution.

7.2 Results

In the survey, more than 50 solutions were reviewed. Of these, 43 were identified as having the potential to provide the information relevant for community contact tracing (Table S7.2). These solutions include 26 smartphone apps, six online surveys and 11 data aggregators, with an additional three being combinations of these solution types. The complete table with all characteristics can be found below [12].

7.2.1 Smartphone Apps

Of the 26 smartphone-based solutions that capture data for the purpose of contact tracing: ten were open source, 17 had implemented approaches for preserving privacy (i.e. no captured identifiable information) and ten required location tracking. Eight apps are actively in use, 13 are under development and five are at a concept stage. All of the active apps are available on both iOS and Android. Privacy policies were available for 17 smartphone apps, but the scope and quality of information varied. Academic institutions or non-profit organizations were associated with 13 of the apps. TraceTogether [13] (Government of Singapore), Aarogya Setu [14] (Government of India) and Rakning C-19 [15] (Iceland's Civil Protection and Emergency Management team) are all government-developed and require users to provide their phone number in order to allow contact tracers to follow up manually if needed (e.g. for risk assessment or testing). Aarogya Setu is available in 11 languages and has been installed more than 50 million times. While solutions like SafePaths [16] (Fig. S7.2), COVID Watch [17] and PACT [18] are based on strict privacy-preserving protocols and only capture data related to proximity and/or location, where other solutions capture a much broader range of information. Some apps like Motus Science [19] capture granular data by using GPS, accelerometer, gyroscope

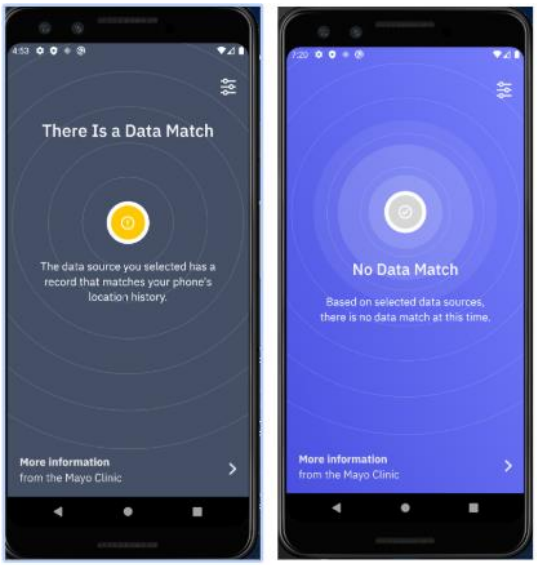

Fig. S7.2. Screen shots of the COVID SafePaths app [16] (system (Image reproduced with permission from MIT/Path Check) used for contact tracing based on GPS-based location history that is collected in a privacy preserving manner. The app alerts a user if a data match indicates that they have been in contact with someone who was diagnosed with COVID-19.

	Active	Development	Concept
**Smartphone App**	TraceTogether [13], COVID Symptom Tracker [33], How We Feel [34], SafePaths [16], Rakning C-19 [15], Aarogya Setu [14], Contact Tracing [35], PACT [18]	CoEpi [32], Covid Watch [17], Ito [36], TraceToZero [37], Guardian [38], Pandoa [39], Safe2 [40], Apple & Google [41], SafeTrace [42], DP-3T [43], CovidSafe [44], PocketCare+ [20], Motus Science [19]	NextTrace [45], Covid App [46], FluPhone [47], PEPP-PT [48], Waze for COVID-19 [49]
**Online Survey**	Coronavirus Survey [21], Covid Near You [22], COVID-19 Symptom Tracker [24], CovApp [23], Epi-Collect [25]		NextTrace [45]
**Data Aggregator**	Corona Data Scraper [28], COVID-19 Mobility Insights [50], Google Mobility Reports [51], Citymapper Mobility Index [52], Social Distancing Scoreboard [30], Corona Map [27], COVID-19 Mobility Data Network [29], Motus Science [19]		EQ Works [53], NSO Surveillance Software [54]

Table S7.2. A list of potential solutions grouped by type and availability status

and magnetometer sensors to determine movement and behavior patterns in real-time at both individual and aggregated levels. PocketCare+ [20] captures proximity, location, symptoms, zip code, and age group, as well as personally identifiable information like IP address. While capturing additional sources of data (e.g. activity and behavior, travel mode and medications) may improve the sensitivity and specificity of contact tracing, the concept of which data inputs and outputs (and their use) provide the maximum benefit merits further investigation.

7.2.2 ONLINE SURVEYS

A total of six online surveys were identified that capture information which could assist in contact tracing efforts. Five of the online surveys are currently active and four of them are associated with academic institutions. Five online surveys are based on interactive dashboards that can be used by users or decision makers to understand symptom patterns and identify areas which pose higher levels of risk. The dashboard provided by COVID-19 Risk Survey (Fig. S7.3) is particularly insightful as it enables the user to visualize several different aspects (e.g. symptoms, demographics, case statistics) over selected time periods. Most of the online surveys (Coronavirus survey [21],

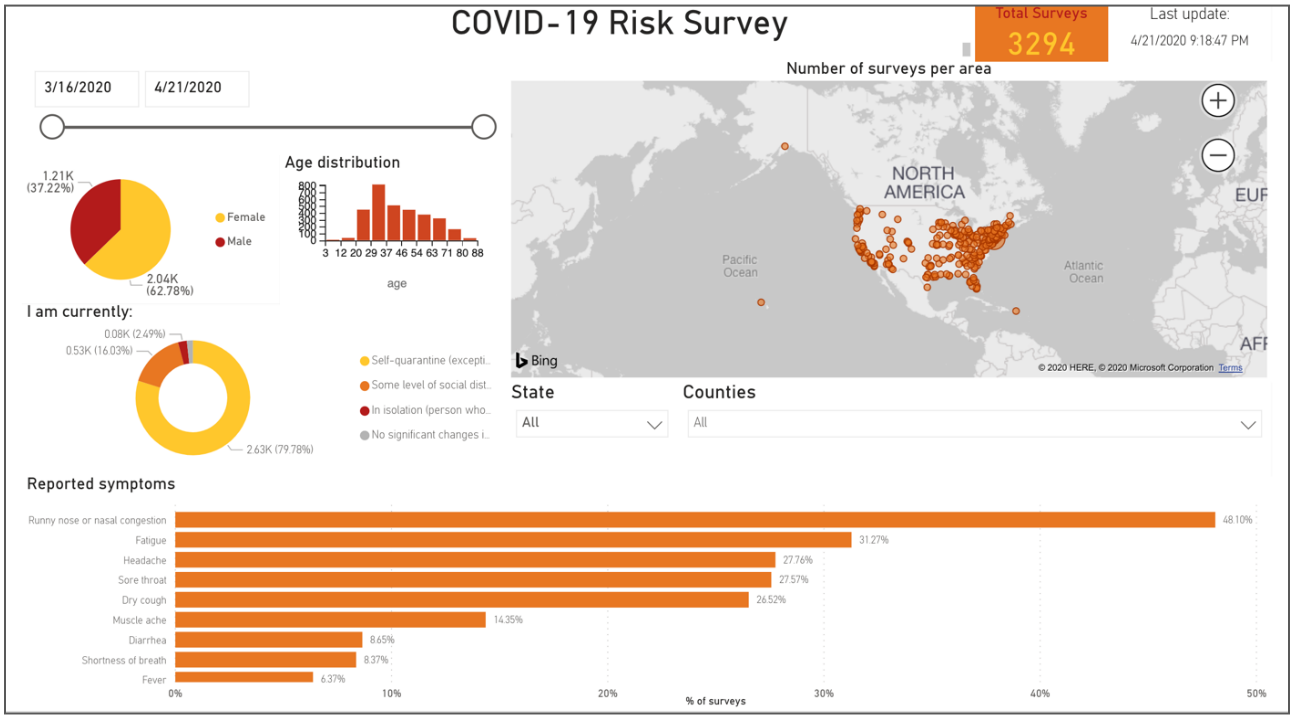

Fig. S7.3. Dashboard provided by the COVID-19 Risk Survey [21] (Image reproduced with permission from Englander Institute for Precision Medicine) showing the demographics and locations of survey responders as well as distribution of symptoms.

COVID Near You [22], CovApp [23] and COVID-19 Symptom Tracker [24]) require users to manually fill out information about their symptoms, location (typically zip code), age or age range, gender, medical conditions, medications, smoking status, and contact with COVID-19 positive individuals. Among the online surveys, Epi-Collect [25] (open source project) is unique in that it requires users to upload their Google location data file (acquired using Google Takeout [26]). Users can review the data and remove any identifiable information in addition to providing a self-report of their symptoms. This enables Epi-Collect to potentially provide standalone contact tracing capabilities without requiring users to download an app that actively tracks their movements. All other online surveys can only be used to augment the data collected by smartphone apps by enabling risk assessment calculations of app users.

7.2.3 Data Aggregators

The eleven selected data aggregators use data collected by existing location services (e.g. Android/iOS) to provide population-level mobility insights and case statistics by location. Eight of these solutions are provided by companies that routinely capture location data of users for their business applications. The other three rely on such datasets but are maintained by either academic institutions, governments, or open source projects. Corona Map [27] (maintained by the Government of South Korea) and Corona Data Scraper [28] (open source project) provide case statistics by location whereas the rest provide population mobility statistics. These solutions act purely as enabling technologies that would enhance risk assessment based on Smartphone apps. Other apps like Motus Science are hybrid solutions combining population-level mobility insights with granular app-based contact tracing (including behavioral patterns such as physical distancing at individual and aggregate levels). Most of these solutions have some form of data visualization to explore the data and understand trends. The dashboards provided by the COVID-19 Mobility Data Network [29] (Fig. S7.4) and Social Distancing Scoreboard (Unacast) [30] standout due to their rich functionality and ease of use. Similarly, Corona Data Scrapper provides up-to-date county-level statistics of COVID-19 cases by pulling data from verified sources in addition to population data.

Data from these data aggregators can be used by decision-makers to rapidly assess trends to prioritize resource allocations and understand how well interventions are working.

The accuracy of these dashboards and aggregators has not been verified as part of this report. Hence, rate of errors, delays or duplication in the data presented by any of the solutions was not determined nor accounted for.

7.3 Discussion and Conclusions

In this review, a broad range of technology-based solutions that could help improve the efficiency, accuracy, and scalability of contact tracing efforts around the world were identified. Many of these solutions are either actively being used or under development, and some are still in concept stage. The diversity

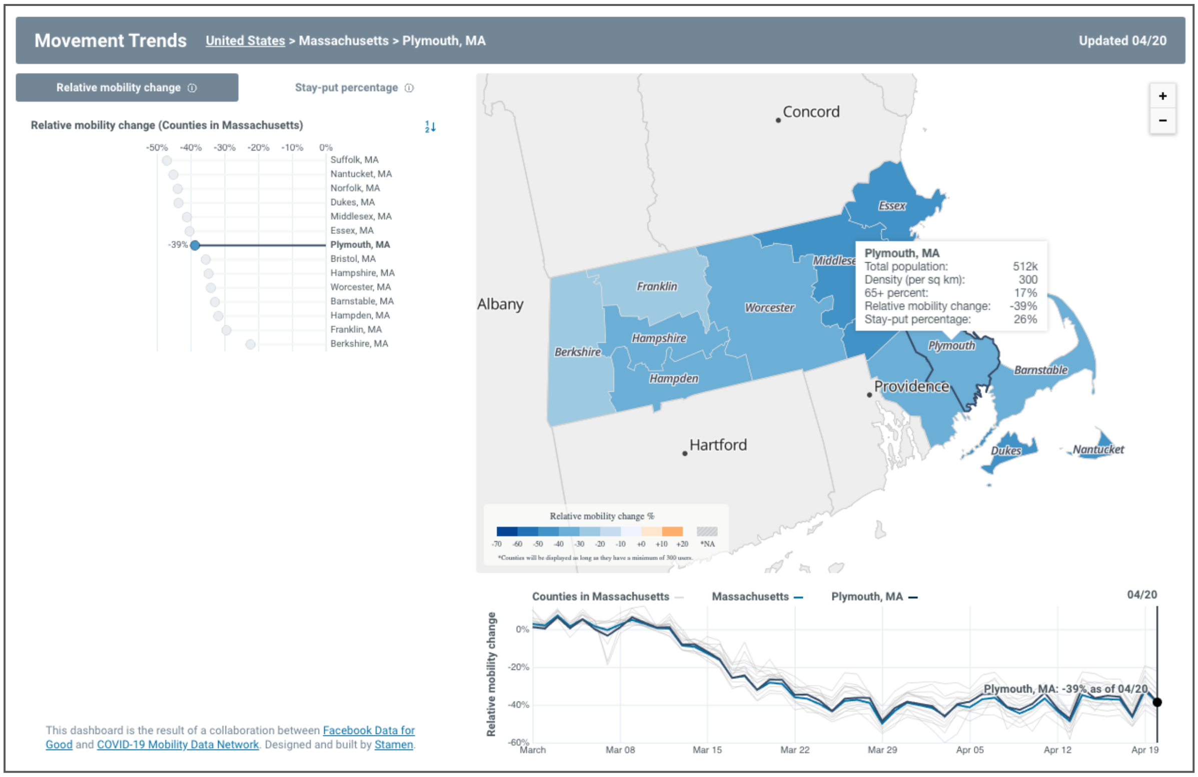

Fig. S7.4. Dashboard of population level mobility insights based on Facebook data generated by COVID-19 Mobility Data Network [29]. Relative changes in mobility are provided at the county level and can also be viewed over time. (Image reproduced with permission from COVID-19 Mobility Data Network)

of these solutions is an indicator of an unprecedented collaborative effort across academia, non-profits, industry, and governments.

Among the three categories of solutions identified, smartphone apps that monitor interaction between people via proximity or location tracking are essential to aid contact tracing efforts. However, online surveys and data aggregators can provide useful information about trends in self-reported symptoms and mobility patterns, which could further assist decision-makers in resource allocation and understanding the impact of policy decisions.

Contact tracing apps that rely on Bluetooth alone have four main shortcomings: 1) insufficient evidence of the ability to achieve the level of voluntary adoption necessary; 2) lack of specificity and sensitivity resulting in a potentially high rate of false positives, primarily due to a lack of spatial and temporal context beyond noting when two users are close to each other; 3) inaccurate estimation of the distance between users and the duration of contact, due to some smartphones' inability to distinguish detection distances between devices; and 4) interference preventing two smartphones from detecting each other regardless of proximity.

For example, proximity, as identified by Bluetooth devices, does not imply sharing a space. People separated by walls, floors, or ceilings, or even travelling on adjoining vehicles, may be identified as being in proximity to each other even though they do not present a disease transmission risk. As a result, Bluetooth systems can record many incorrect matches while missing many actual matches. Consequently, manual contact tracing must remain a central element of this process; buoyed by technologies that provide spatial and temporal context, as well as behavioral and movement insights.

The solutions identified differed significantly in the type of data captured. While some solutions were, by design, privacy preserving, others captured sensitive information such as phone numbers, medical history, and IP addresses. Because of the sensitive nature of the information that contact tracing solutions may collect, users should consider the following factors:

- Privacy: To what extent does the solution preserve privacy of an individual?
- Data Security: How does the solution securely collect and store data?
- Data Use: How will the data be used, who will use the data and for how long? When will the data be deleted?
- Organization: What kind of organization is responsible for and manages the solution?

In order for contact tracing solutions to be successful, some of the most relevant requirements include:

- Broad adoption by the population: According to some estimates [31], approximately 60% of the population will need to sign up and actively use the solution in order for it to be successful. Singapore currently has less than 20% adoption despite surveys showing Singaporeans are more relaxed about personal privacy concerns than people in many other countries. In Iceland, 40% adoption has been achieved after launching on 17 April 2020. Australia is planning to release an app modelled after the Singapore system and the government is aiming for adoption rates of 40-60%. Even factors like increased power consumption, resulting in a reduction in battery life, may have an impact on user experience and acceptability.
- Minimize fragmentation and encourage data sharing: While proliferation of contact tracing solution is a positive trend, it could lead to fragmentation of users across multiple ecosystems and result in reducing their effectiveness. One way to mitigate this situation is by encouraging solutions to share data by using a model similar to SafePaths [16], Covid Watch [17] and CoEpi [32].
- Strong privacy and ethical guidelines: Solutions may capture sensitive personal information and will therefore require adherence to strong privacy and ethical standards.
- Confirmation of diagnosis: Timely reporting of confirmed diagnosis will need to become a central part of clinical practice in order for contact tracing efforts to be successful at containing the spread of infections. This will depend on whether users consent to sharing this information, as well as training and adoption of a case reporting protocol by clinical staff.

The design and deployment of a successful technology-enabled contact tracing system must account for these aspects in order to provide an appropriate balance between amount of data capture, sensitivity and specificity, and privacy considerations. Systems that concurrently consider symptoms, risk profile; manual and Bluetooth-based contact tracing; spatial and temporal context; and measurement of movement of people in the community setting are likely to provide deeper understanding of the level of community transmission and enable appropriate containment actions. Particularly given the significant levels of asymptomatic transmission reported in COVID-19. Importantly, such solutions will enable rapid detection of any new waves of infection and inform the implementation of physical distancing and other measures to limit the infection's further spread.

References

[1] K. T. D. Eames and M. J. Keeling, "Contact Tracing and Disease Control," *Proc. R. Soc. London. Ser. B Biol. Sci.*, vol. 270, no. 1533, pp. 2565–2571, Dec. 2003

[2] "Baker-Polito Administration Announces COVID-19 Community Tracing Collaborative to Further Mitigate the Spread of Virus | Mass.gov." [Online]. Available: https://www.mass.gov/news/baker-polito-administration-announces-covid-19-community-tracing-collaborative-to-further. [Accessed: 28-June-2020]

[3] "Amid Ongoing COVID-19 Pandemic, Governor Cuomo and Mayor Mike Bloomberg Launch Nation-Leading COVID-19 Contact Tracing Program to Control Infection Rate | Governor Andrew M. Cuomo." [Online]. Available: https://www.governor.ny.gov/news/amid-ongoing-

covid-19-pandemic-governor-cuomo-and-mayor-mike-bloomberg-launch-nation-leading. [Accessed: 28-June-2020]
[4] L. Ferretti *et al.*, "Quantifying SARS-CoV-2 Transmission Suggests Epidemic Control with Digital Contact Tracing," *Science*, vol. 368, no. 6491, eabb6936, Mar. 2020
[5] M. Salathé *et al.*, "Digital Epidemiology," *PLoS Comput. Biol.*, vol. 8, no. 7:e1002616, Jul. 2012
[6] "Singapore Government Launches New App for Contact Tracing to Combat Spread of COVID-19 | MobiHealthNews." [Online]. Available: https://www.mobihealthnews.com/news/asia-pacific/singapore-government-launches-new-app-contact-tracing-combat-spread-covid-19 [Accessed: 28-June-2020]
[7] O. Park *et al.*, "Contact Transmission of Covid-19 in South Korea: Novel Investigation Techniques for Tracing Contacts," *Osong Public Heal. Res. Perspect.*, no. 1, pp. 60–63, 2020
[8] S. Park, G. J. Choi, and H. Ko, "Information Technology Based Tracing Strategy in Response to COVID-19 in South Korea Privacy Controversies," *JAMA*, Apr. 2020 [ePUB]
[9] R. Raskar *et al.*, "Apps Gone Rogue: Maintaining Personal Privacy in an Epidemic," *arXiv*:2003.08567, Mar. 2020
[10] M. Shoaib, H. Scholten, and P. J. M. Havinga, "Towards Physical Activity Recognition Using Smartphone Sensors," in *Proceedings - IEEE 10th International Conference on Ubiquitous Intelligence and Computing, UIC 2013 and IEEE 10th International Conference on Autonomic and Trusted Computing, ATC 2013*, pp. 80–87, 2013
[11] "NOVID. Stop COVID-19 with Ultrasonic Accuracy" [Online]. Available: https://www.novid.org/. [Accessed: 28-June-2020]
[12] S. Patel and J. Mantilla, "A database of contact tracing solutions." [Online]. Available: https://tinyurl.com/contact-tracing. [Accessed: 28-June-2020]
[13] "TraceTogether." [Online]. Available: https://www.tracetogether.gov.sg/. [Accessed: 28-June-2020]
[14] "Aarogya Setu Mobile App." [Online]. Available: https://www.mygov.in/aarogya-setu-app/. [Accessed: 28-June-2020]
[15] "Rakning C-19 App." [Online]. Available: https://www.covid.is/app/en. [Accessed: 28-June-2020]
[16] "COVID SafePaths." [Online]. Available: https://covidsafepaths.org/. [Accessed: 28-June-2020]
[17] "Covid Watch." [Online]. Available: https://www.covid-watch.org/article. [Accessed: 28-June-2020]
[18] "PACT: Private Automated Contact Tracing." [Online]. Available: https://pact.mit.edu/. [Accessed: 28-June-2020]
[19] "Motus Science Mobility Data Insights." [Online]. Available: https://motusscience.com/. [Accessed: 28-June-2020]
[20] W. Dong, T. Guan, B. Lepri, and C. Qiao, "PocketCare," *Proc. ACM Interactive, Mobile, Wearable Ubiquitous Technol.*, vol. 3, no. 2, pp. 1–23, Jun. 2019
[21] "Coronavirus Survey - Weill Cornell Medicine." [Online]. Available: https://covid19.eipm-research.org/home. [Accessed: 28-June-2020]
[22] "COVID Near You." [Online]. Available: https://covidnearyou.org/#!/. [Accessed: 28-June-2020]
[23] "CovApp." [Online]. Available: https://covapp.charite.de/. [Accessed: 28-June-2020]
[24] "COVID-19 Symptom Tracker - University of Alabama." [Online]. Available: https://www.helpbeatcovid19.org/. [Accessed: 28-June-2020]
[25] "Epi-Collect - Donate Google location data to fight COVID-19." [Online]. Available: https://epi-collect.org/. [Accessed: 28-June-2020]
[26] "Google Takeout." [Online]. Available: https://takeout.google.com/. [Accessed: 28-June-2020]
[27] "Corona Map - South Korea." [Online]. Available: https://coronamap.site/. [Accessed: 28-June-2020]
[28] "Corona Data Scraper." [Online]. Available: https://coronadatascraper.com/#home. [Accessed: 28-June-2020]
[29] "COVID-19 Mobility Data Network." [Online]. Available: https://www.covid19mobility.org/. [Accessed: 28-June-2020]
[30] "Covid-19 Social Distancing Scoreboard — Unacast." [Online]. Available: https://www.unacast.com/covid19/social-distancing-scoreboard. [Accessed: 28-June-2020]
[31] R. Hinch *et al.*, "Effective Configurations of a Digital Contact Tracing App: A report to NHSX," Apr. 2020 [Online]. Available: https://github. com/BDI-pathogens/covid-19_instant_tracing/blob/master/Report [Accessed: 28-June-2020]
[32] "CoEpi: Community Epidemiology in Action." [Online]. Available: https://www.coepi.org/. [Accessed: 28-June-2020]
[33] "COVID Symptom Tracker - Help slow the spread of COVID-19." [Online]. Available: https://covid.joinzoe.com/us. [Accessed: 28-June-2020]
[34] "How We Feel." [Online]. Available: https://howwefeel.org/. [Accessed: 28-June-2020]
[35] "Contact Tracing: Know more of your surroundings." [Online]. Available: https://contacttracing.mobi/. [Accessed: 28-June-2020]
[36] "ito App." [Online]. Available: https://www.ito-app.org/. [Accessed: 28-June-2020]
[37] "TraceToZero." [Online]. Available: https://www.tracetozero.org/. [Accessed: 28-June-2020]
[38] "Guardian - Anonymous Pandemic Tracer." [Online].

Available: https://www.myguardian.life/. [Accessed: 28-June-2020]
[39] "PANDOA App - Corona Virus Tracer." [Online]. Available: https://pandoa.org/en/. [Accessed: 28-June-2020]
[40] "Safe2 Mobile App." [Online]. Available: https://www.indiegogo.com/projects/safe2-mobile-app-beating-covid-19-coronavirus#/. [Accessed: 28-June-2020]
[41] "Privacy-Preserving Contact Tracing - Apple and Google." [Online]. Available: https://www.apple.com/covid19/contacttracing. [Accessed: 28-June-2020]
[42] "SafeTrace: April 2020 Development Update - Enigma." [Online]. Available: https://blog.enigma.co/safetrace-april-2020-development-update-4c5f77bfddfa. [Accessed: 28-June-2020]
[43] "DP-3T/documents: Decentralized Privacy-Preserving Proximity Tracing -- Documents." [Online]. Available: https://github.com/DP-3T/documents. [Accessed: 28-June-2020]
[44] "CovidSafe." [Online]. Available: https://covidsafe.cs.washington.edu/. [Accessed: 28-June-2020]
[45] "NextTrace." [Online]. Available: https://nexttrace.org/. [Accessed: 28-June-2020]
[46] "Covid App." [Online]. Available: https://www.covid-app.io/. [Accessed: 28-June-2020]
[47] "Fluphone Project." [Online]. Available: https://www.cl.cam.ac.uk/research/srg/netos/projects/archive/fluphone/. [Accessed: 28-June-2020]
[48] "PEPP-PT - Pan-European Privacy-Preserving Proximity Tracing." [Online]. Available: https://www.pepp-pt.org/. [Accessed: 28-June-2020]
[49] "Waze for COVID-19 App." [Online]. Available: https://su.org/blog/unleashing-innovation-in-pandemics-dr-daniel-kraft-and-the-waze-for-covid-19-app/. [Accessed: 28-June-2020]
[50] "COVID-19 Mobility Insights - Cuebiq." [Online]. Available: https://www.cuebiq.com/visitation-insights-covid19/. [Accessed: 28-June-2020]
[51] "Google COVID-19 Community Mobility Reports." [Online]. Available: https://www.google.com/covid19/mobility/. [Accessed: 28-June-2020]
[52] "Citymapper Mobility Index." [Online]. Available: https://citymapper.com/CMI. [Accessed: 28-June-2020]
[53] "EQ Works." [Online]. Available: https://www.eqworks.com/. [Accessed: 28-June-2020]
[54] "NSO GROUP." [Online]. Available: https://www.nsogroup.com/. [Accessed: 28-June-2020]

mHealth Data Integration Platforms

8.1 INTRODUCTION

Mobile health (mHealth) technologies have the potential to fundamentally transform healthcare around the world by enabling more objective and personalized clinical outcomes; improved efficiency when deploying clinical personnel; broadening access to medical care through remote monitoring; and an overall reduction of healthcare costs [1]. Healthcare systems can leverage the strengths of mHealth to address the COVID-19 pandemic [2], as, with their widespread adoption by medical facilities, these technologies can demonstrate the tremendous impact they can have in alleviating the burdens on an already strained healthcare ecosystem.

Such technologies can enable 1) the safe and effective collection of electronic Patient-Reported Outcomes (ePROs) [3] such as daily symptoms diaries (for more information, see Section 3 “Using ePRO Solutions to Screen and Monitor COVID-19 Cases”); 2) the collection of physiological data using wearable sensors [4] and other medical devices [5] to, for example, monitor symptoms in COVID-19 patients and among healthcare workers (see Sections 2 “Monitoring Service Providers and Patients in a Disaster Scenario” and 4 “Remote Monitoring of Patients with COVID-19 and Frontline Healthcare Workers Using mHealth Technologies”); 3) the implementation of digital contact tracing in clinical environments [6] and in the community [7] (see Sections 6 “Technology-assisted Contact Tracing in the Hospital Setting” and 7 “Technology-Based Contact Tracing Solutions to Contain the Spread of COVID-19 in the Community”).

In order to obtain the benefits of utilizing mHealth solutions such as those described above, it is necessary to integrate the data from mHealth technologies into a facility’s established flow of clinical decision-making.

Generally, clinicians do not have the time to review the enormous amount of data generated by these technologies, nor should it be expected of them to be willing to examine multiple unrelated applications and sensing devices to gather the applicable clinical information. Moreover, having a variety of concurrent systems monitoring similar parameters without synchronization may lead an overwhelming amount of information that could stymy clinical decision-making and responses. These challenges represent an obstacle to adopting mHealth technologies particularly in scenarios where timeliness is critical, such as the present COVID-19 pandemic.

Ideally, data from different mHealth sources, such as ePROs and wearable sensing devices, should be 1) continuously collected; 2) automatically synchronized and harmonized; 3) aggregated into a single system with an easy-to-use interface; 4) fully integrated into Electronic Health Record (EHR) systems; and 5) be able to perform advanced analytics on the collected data, triggering robust alarms only when clinical reviews or intervention are needed. A schematic representation of the desired characteristics of a generic mHealth data integration platform is shown in Figure S8.1.

In the last few years many private companies have taken up this challenge, and data integration platforms have been successfully used in clinical trials and research studies by academic institutions, healthcare providers, and pharmaceutical companies around the world [8]–[12]. When used, they have proven to be invaluable tools by significantly reducing the resources and time needed to obtain significant results. But their use also shows that evaluating these systems can take significant amounts of time, something that must be minimized in an emergency scenario.

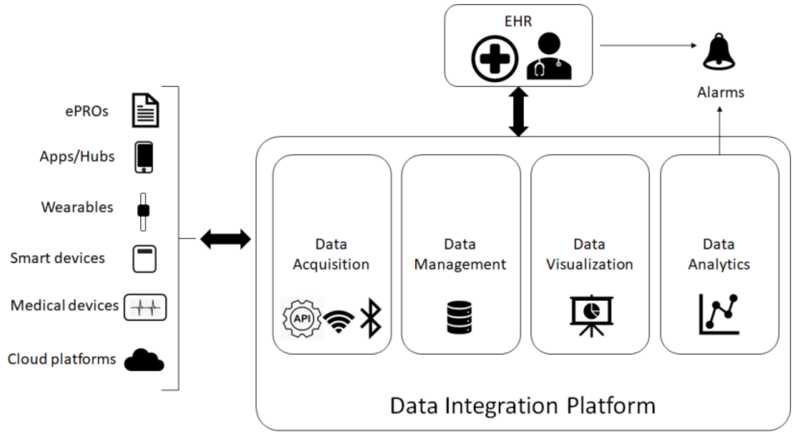

Fig. S8.1 Schematic representation of the main characteristics of a generic mHealth data integration platform.

This report aims to provide relevant information to accelerate that process. As this report’s analysis has been carried out without precise specifications on the type of devices, data or systems to be integrated, we focused on generic characteristics of mHealth data integration platforms. With the intent to help development teams accelerate their decision-making processes once the underlying technologies have been selected.

8.2 METHODOLOGY

The process of identifying mHealth data integration solutions relied mostly on three factors: 1) prior knowledge of the team; 2) web search of data integration solutions focusing on healthcare; 3) discussion with other members of the scientific community working on mHealth or within healthcare networks.

This process resulted in the selection of eleven platforms: Adaptive Clinical (Adaptive Clinical Systems, NJ, USA), Artemis (University of Ontario, ON, Canada), Care Evolution

(Care Evolution Inc., MI, USA), ERT (ERT Clinical, PA, USA), InterSystems (InterSystems Corporation, MA, USA), Medable (Medable Inc., CA, USA), PhysIQ (PhysIQ, IL, USA), Redox (Redoc Inc., WA, USA), TrialStat (TrialStat Solution Inc., QC, Canada), Validic (Validic Inc. NC, USA), and Xealth (Xealth, WA, USA).

It is important to point out that the chosen solutions do not represent an exhaustive list of all possible vendors, but only a sampling of the options currently available on the market. Many more potential platforms exist. For example, some of the ePROs solutions presented in Section 3 can integrate sensor data and interact with EHR systems as well. Similarly, many of the platforms considered in this section have the capability to incorporate ePRO data. The division of platforms between the two sections is based on the teams' a priori knowledge and the primary focus of the platform (either specifically ePROs or, more generally, health data integration) claimed by the vendors.

It should be noted that specific product depictions, illustrations or descriptions should not be considered endorsements, recommendations or specific criticisms on the part of the MGB network or its affiliated institutions or any of the Institutions that the authors of this report are affiliated with.

#	Question
1	Please provide an overview of your platform.
2	Please describe how your platform captures data from third party sensors, applications, etc.
3	Describe how your platform would manage the collected data?
4	Describe how your platform would integrate with EPIC and/or other EHR systems.
5	Can your platform upload patient contextual data from EPIC and/or other EHR systems to incorporate it into its diagnostic evaluation and algorithms?
6	Can your platform perform analysis on mHealth data?
7	Describe the user interface that clinical personnel would use.
8	Describe your regulatory/compliance status.
9	Describe your deployment timeline.
10	Describe your support modalities provided during the process.
11	Does your platform include any COVID-19 specific solution?
12	Costs.

Table S8.1. Twelve questions survey answered by the vendors

The details of each platform's capabilities were retrieved through direct contact with the vendors and asking them to complete a twelve-question survey. When contacted, vendors were also encouraged to provide existing materials, and links to relevant backup material, along with their written answers. EPIC was chosen as the primary EHR system example due to its prevalence among healthcare systems. The 12 questions, shown in Table S8.1, were formulated with the intent of gathering enough information on the platform so that the following three overarching questions could be answered:

1. Can the system acquire the necessary data?
2. Can the data be transformed into the information clinicians need to make decisions in a timely fashion?
3. Can the solution be implemented quickly enough to be relevant?

The following subsections will provide further insight on each of these points.

8.3 RESULTS

A summary of the main attributes of each mHealth data integration platform is shown in Table S8.2 (see page 61).

Of this report, two things should be recognized. First, the listed capabilities of the platforms may not be complete, as these systems are under continuous development and new features may be added. Secondly, those indicated capabilities are based solely on the vendors' claims and publicly available information. The limited timeframe available for this analysis did not allow us to perform an independent and objective assessment of the platforms.

8.3.1 MHEALTH DATA ACQUISITION AND MANAGEMENT

The first question that needs to be answered when selecting a mHealth data integration solution is whether the platform can acquire the data needed to make the necessary clinical decisions [13]–[16]. In the case of COVID-19, of interest are the data types belonging to one of the following categories:

- Continuous streaming physiological waveform data (e.g. electrocardiogram (ECG), accelerometry, photoplethysmography (PPG), acoustic).
- High-frequency periodic sensor-firmware generated vital signs (e.g. Heart Rate (HR) or Respiratory Rate (RR) @1Hz – 1/60Hz, Skin Temperature, etc.)
- High frequency, cloud-generated vital signs from sensor waveforms (e.g. HR, RR, Heart Rate Variability (HRV), posture, body tilt, activity level, step counts, sleep, heart activity monitoring measures, such as Atrial Fibrillation (A-Fib) or Interbeat Intervals (IBI) @1Hz – 1/60Hz, etc.)
- Periodic cloud-generated statistical aggregates (e.g. hourly N^{th} percentile of HR or RR, Activity aggregates, etc.).
- Periodic/Aperiodic point measures (e.g. single/repeated measure(s) from weight scale or blood pressure cuff).
- Notifications and alarms triggered by stand-alone apps (e.g. warning notifications sent by a monitoring app when a parameter of interest exceeds a danger threshold, etc.)
- Questionnaire responses (ePROs) via apps or text messaging which include numeric values, text, sliding scale, single-select radio button, multi-select checkbox, date/time, body map, voice/audio capture, etc.

These types of data can be captured in a variety of different ways, including:

- Continuous streaming acquisition via Bluetooth from sensing devices to a phone app, uploaded at configurable intervals over Wi-Fi or cellular to a cloud platform.
- Continuous streaming acquisition from sensors and the direct upload of data over Wi-Fi or cellular when radio connectivity is built-in the devices.
- Aperiodic point measure captured via Bluetooth acquisition from a smart or medical device or manually entered into questionnaires on phone apps. Some

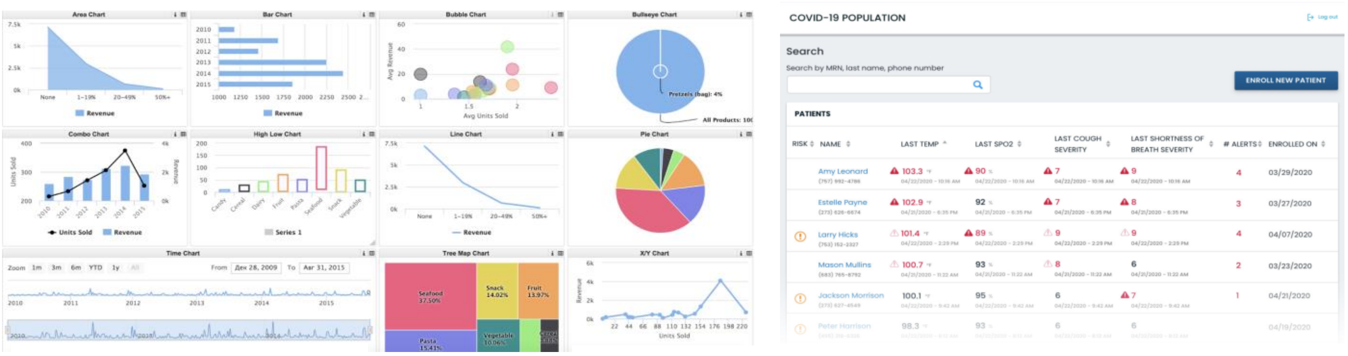

Figure S8.2. Example of web-based data visualization dashboards: (a) InterSystems; (b) Validic's COVID-19 specific solution.

solutions, such as Validic, allow the user to upload pictures of medical devices' screens showing the measurement value and automatically integrate the point measure in the system.

- Cloud-to-cloud integration via platform APIs. For sensors that only upload to their cloud, data must be exported from there. In this category fall the integration of data through health data hubs, such as Google Fit and Apple HealthKit.
- 5G-enabled sensors, when available, will offer 'direct-to-cloud' data export, in areas where 5G service is available. 5G may eliminate the 'data-relay' challenges of modalities listed above, but further evidence is needed to prove the potential advantages of this approach.

The exchange of data with the platforms using these modalities can be technically achieved in different ways. In most cases, APIs compatible with standards such as Health Level Seven (HL7) (CDA, CCD, etc.) [17] or Fast Healthcare Interoperability Resources (FHIR) [18] are used. Some platforms, such as Validic, Adaptive Clinical, and PhyIQ, establish direct partnerships with the device's vendors and create ad-hoc APIs to be integrated into their SDKs.

Data integration platforms need to address malfunctions, data and connectivity loss, and implement, when not done directly by 3rd party software, robust strategies to deal with missing data. For example, if cellular signal or Wi-Fi connectivity is lost, acquired data is stored locally onboard the smart device and will be uploaded when a connection is reestablished.

Another essential aspect to be considered to achieve robust interoperability between these data types and sensing devices is the synchronization between the different technologies. In some applications, precise temporal device synchronization may be critical to provide a robust and reliable monitoring system. For example, a monitoring system for COVID-19 might include ECG, thoracic bioimpedance, activity levels, oxygen saturation level (SpO2), and periodic temperature measurements. It is unlikely that all these sensing sources would be included in a single system. Orthogonal measures of the same physiological parameter and data fusion by different sources could help, in this case, optimally handle motion artifacts (e.g. HR by ECG, verified by acoustic sensing of heart tones, or PPG). In this scenario, the data integration platform should act as a "mediator" between the different technologies and provide the capability to accurately synchronize the devices' signals. In other cases, it would be sufficient to identify the coarse temporal sequence of events, where a slight time offset between diverse data sources can be tolerated (e.g. skin temperature reading and blood pressure measurements).

None of our questions explicitly addressed this aspect. However, our understanding is that these platforms generally rely on the timestamps provided by the internal clock of the devices or applications (which are often synched to an online clock) and perform checks for the validity and consistency of the temporal information without explicit synchronization between devices. More advanced synchronization would need to be further discussed with the platforms' vendors.

Data from these heterogeneous sources need to be adequately processed and transformed before they can be integrated into the decision-making process. Data harmonization is an important feature provided by these platforms, and it is achieved in a variety of ways. In most cases, ad-hoc modules or APIs for a particular device or application are developed for parsing and converting the original data into a platform-specific data format. In the Adaptive-Clinical solution (Table S8.2, page 61), for example, interoperability is achieved exploiting "connector" modules, designed for a precise eSource, which extract and transform the underlying data in a standard format and move it across a "virtual bus," to which all the other elements of the system are connected. Once the standard data packet reaches its destination(s), it is reconverted to its original format.

In addition to sensor-generated data and ePROs, demographic, comorbidity, and other related information that exists in the EHR system may be useful to support clinical decisions.

8.3.2 mHealth Data Visualization and Integration with Existing EHR Systems

Once the data has been acquired, the next question is whether it can be transformed into interpretable information which clinical personnel could use to make timely decisions.

The first element that needs to be considered is the how the information is presented to clinicians. Clinical staff cannot be expected to log in to multiple systems and integrate the information through a cumbersome process any time they need to utilize such information. Moreover, the data needs to be

displayed in an organized, clean, and intuitive way to avoid overwhelming users with unimportant information [19]–[22].

Ideally, all relevant data should be integrated into an existing EHR system, which clinicians are like to already utilize in their day-to-day. However, EHR systems are generally not designed to manage and present large amounts of continuous data, such as physiological time-series, from heterogeneous sources and do not have the built-in capabilities to automatically aggregate these types of data to increase their interpretability. Relying on external, stand-alone tools for data visualization, in this case, may be a better option.

The solutions considered in this report would allow clinicians to visualize mHealth data 1) through a web-based portal and 2) directly from the EHR user interface. In the first case, the platforms' vendors would have created custom user interfaces that can efficiently and intuitively display all the types of data supported by the platform, time-series data, aggregated statistics, and advanced dashboards. A few examples of these web-based dashboards are shown in Figure S8.2.

Data visualization that has been integrated with an EHR user interface, on the other hand, is more limited and constrained by the capabilities of the EHR system itself. Only high-level or aggregated data will likely be displayed via the EHR interface since the ability to show continuous low-level data may be absent. One possible solution adopted by data integration platforms, such as Xealth, is to provide access to third-party mHealth data dashboards (e.g. the cloud-based portal of a wearable device) through iFrames integrated into the EHR interface. This method allows clinicians to view the mHealth data through a user interface specifically designed for that technology. The drawback is that the data can only be visualized and not directly integrated into the EHR systems.

The actual integration with the EHR systems can happen at multiple levels. For example, some platforms can only upload data from different eSources to the EHR system or download information such as demographics, comorbidities, etc. from the EHR system to their internal database. Other solutions support the bidirectional exchange of information between the two systems, integration of EHR data into the platform analytics and even creation/triggering of alerts within the EHR system.

These functionalities are mainly achieved using data transmission protocols compatible with HL7 or FHIR standards.

8.3.3 mHealth Data Analysis

The ability to perform different levels of analysis on mHealth data [23], [24] is the aspect in which we observed the most differences among the data platforms. Some of the considered solutions, such as Adaptive Clinical, Redox, ERT, and Xealth, are strongly focused on the data transfer, interoperability, and integration with existing EHR systems and provide little to no analytics capabilities (but data can still be analyzed using 3rd party applications). Other platforms only support simple data analysis (e.g. based on thresholds, moving averages, etc.) and the creation of rule-based automatic care alerts. Most platforms, however, support complex data analysis (e.g. multivariate time-series analysis of trends). The use of Artificial Intelligence (AI) [25], [26] to extract easily interpretable information from the incredible amount of data generated by mHealth technologies may represent an additional tool to speed-up and increase the accuracy of the clinical decision-making process. For example, PhysIQ supports a variety of machine learning and deep learning-based analytics and has developed a proprietary AI algorithm with 510(k)-clearance by the Food and Drug Administration (FDA) to "learn" the relationships and patterns of an individual's vital signs, from physiological data captured by biosensors, to establish a dynamic personal baseline. Similarly, Care Evolution, InterSystems, and Medable support AI tools for the development of predictive algorithms [27] or the extraction of digital biomarkers [28].

One of the great promises of mHealth continuous monitoring is the ability to identify changes in patient conditions that might require urgent attention. This modality is in stark contrast to current use cases involving the uploading of non-life-critical patient data, such as body weight, which can be reviewed asynchronously and without consequences. To implement this important capability, however, the approach to generating and communicating alarms, alerts, and clinical guidance must be thought through very carefully. Some of the systems purport to have this option, but real-time alerting and clinical guidance to a responsible clinician is actually a significant paradigm shift. Implementing real-time clinical guidance for potentially life-critical patient status changes will encourage system designers to be very conservative in not missing a potential issue. However, if they are too conservative, the system will overload clinicians with unnecessary or duplicative alarms. As a result, integrated protocols will likely need to be designed, customized, implemented, and 'owned' by the provider organizations.

There is also the critical issue of whether the EHR system is architected to provide alarms to clinicians in a timely fashion. For example, some systems may be designed to be updated in a batch fashion once per day. This approach would not be frequent enough to provide timely alarms in many clinical situations.

8.3.4 Rapid Implementation

Although it may seem logical to look to mHealth technology vendors to enhance existing solutions, the reality has proven to be quite different. Existing solutions were designed for non-life-critical, non-real-time patient data upload. Adding mHealth data to patient contextual information from an EHR system to create alarms, alerts, and clinical guidance is effectively practicing medicine. The clinical practice of medicine, and the responsibility for it, is owned by medical professionals who practice under the guidance of sophisticated healthcare provider organizations whose principal responsibility is to aggregate, codify, disseminate, and enforce clinical practice guidelines under their enterprise governance and self-allocation of liability.

During the COVID-19 crisis, however, implementation speed may be more important than designing the best system. This condition may create a situation where some of the traditional approaches are adjusted. On this note, the FDA recently issued the Emergency Use Authorizations (EUAs) for medical devices in an attempt to relax the regulatory constraints for the use of these technologies to diagnose, treat, or prevent COVID-19.[1] However, there remain regulatory, liability, and

practical constraints about how far the implementation can stray from the accepted process. We evaluated multiple factors that may impact the timely implementation and deployment of data integration solutions.

8.3.5 Compliance and Regulatory

Most of the considered platforms are compliant with the Health Insurance Portability and Accountability Act (HIPAA). Care Evolution and Adaptive Clinical have similar certifications for privacy protection and data security (GDPR and GxP, respectively). Many of the providers have additional certifications (e.g. FDA 21 CRF Part 11, SOC1/SOC2, etc.). The continuous efforts of vendors to obtain these certifications are an indicator of the increasing quality that these platforms are trying to achieve in providing their services.

Another element that could help to accelerate the adoption of these technologies is the availability of specific COVID-19 solutions. These are meant to be adaptations of the original platforms offering mostly simplified and targeted capabilities tailored to the needs of healthcare systems for fighting COVID-19, and that could be quickly deployed. Seven out of the eleven vendors have already implemented solutions specific to COVID-19.

Finally, the last two elements to consider for the rapid implementation of the examined solutions are deployment timelines and costs. Deployment can range from a few days for simple solutions that include only modules specific for COVID-19 (e.g. Validic's Covid-19 Rapid Deployment Remote Monitoring, which can be deployed in less than a day) to months for a customized total-health solution, including all the levels of integration described in this report. Costs can also be highly variable. Many of the companies that developed these platforms already have agreements in place with healthcare networks, research institutions, and pharmaceutical companies. Since all the considered platforms have the capability to integrate data from mHealth technologies, even with different levels of complexity and features, we believe that exploiting existing collaborations between healthcare systems and data integration platforms, when present, can bring a considerable advantage in terms of deployment time and costs.

8.4 Conclusions

Our analysis has shown that most of the currently available mHealth data integration platforms on the market have similar capabilities and can enable, with slightly different approaches, 1) the acquisition of multiple data types that could be relevant for the management of COVID-19; 2) the integration of this data into existing EHR systems; 3) the efficient visualization and analysis of mHealth data; and 4) a rapid and secure implementation of the solutions.

The precise specifications on the kind of technologies that need to be adopted are required in order to identify the most suitable solution. Moreover, integration with EHR systems is necessary and desirable, but relying solely on EHR for managing, visualizing, and analyzing this enormous amount of data may not be the best option, especially when complex, time-series-derivate signals are considered. Dashboards specifically designed for this kind of data and advanced analytics tools may elevate the quality and interpretability of the information that is possible to extract from mHealth data. Finally, for timely and cost-effective implementation, aspects such as the platform's compliance status, the availability of specific COVID-19 solutions, and the presence of in-progress collaboration between healthcare systems and mHealth data integration platforms, should be considered.

References

[1] R. S. Istepanian and B. Woodward, *M-health: Fundamentals and Applications*. John Wiley & Sons, 2016

[2] X.-R. Ding *et al.*, "Wearable Sensing and Telehealth Technology with Potential Applications in the Coronavirus Pandemic," *IEEE Rev. Biomed. Eng.*, 2020 [ePUB]

[3] S. J. Coons, S. Eremenco, J. J. Lundy, P. O'Donohoe, H. O'Gorman, and W. Malizia, "Capturing Patient-Reported Outcome (PRO) Data Electronically: The Past, Present, and Promise of ePRO Measurement in Clinical Trials.," *Patient*, vol. 8, no. 4, pp. 301–9, Aug. 2015

[4] A. Godfrey, V. Hetherington, H. Shum, P. Bonato, N. H. Lovell, and S. Stuart, "From A to Z: Wearable technology explained," *Maturitas*, vol. 113, pp. 40–47, Jul. 2018

[5] Sohmyung Ha *et al.*, "Integrated Circuits and Electrode Interfaces for Noninvasive Physiological Monitoring," *IEEE Trans. Biomed. Eng.*, vol. 61, no. 5, pp. 1522–1537, May 2014

[6] M. N. Kamel Boulos and G. Berry, "Real-time Locating Systems (RTLS) in Healthcare: a Condensed Primer," *Int. J. Health Geogr.*, vol. 11, no. 1, p. 25, 2012

[7] L. Ferretti *et al.*, "Quantifying SARS-CoV-2 Transmission Suggests Epidemic Control with Digital Contact Tracing," *Science*, vol. 368, no. 6491, p. eabb6936, May 2020.

[8] K. Ovaska *et al.*, "Large-scale Data Integration Framework Provides a Comprehensive View on Glioblastoma Multiforme.," *Genome Med.*, vol. 2, no. 9, p. 65, Sep. 2010

[9] P. Coorevits *et al.*, "Electronic Health Records: New Opportunities for Clinical Research," *J. Intern. Med.*, vol. 274, no. 6, pp. 547–560, Dec. 2013

[10] B. Rance, V. Canuel, H. Countouris, P. Laurent-Puig, and A. Burgun, "Integrating Heterogeneous Biomedical Data for Cancer Research: the CARPEM infrastructure," *Appl. Clin. Inform.*, vol. 7, no. 2, pp. 260–274, Apr. 2016

[11] B. Haarbrandt *et al.*, "HiGHmed – An Open Platform Approach to Enhance Care and Research across Institutional Boundaries," *Methods Inf. Med.*, vol. 57, no. S01, pp. e66–e81, Jul 2018

[12] M. Jayaratne *et al.*, "A Data Integration Platform for Patient-Centered e-Healthcare and Clinical Decision

Support," *Futur. Gener. Comput. Syst.*, vol. 92, pp. 996–1008, Mar. 2019
[13] R. Whittaker, "Issues in mHealth: Findings From Key Informant Interviews," *J. Med. Internet Res.*, vol. 14, no. 5, p. e129, Oct. 2012
[14] G. Mehl and A. Labrique, "Prioritizing Integrated mHealth Strategies for Universal Health Coverage," *Science*, vol. 345, no. 6202, pp. 1284–1287, Sep. 2014
[15] J. Franz-Vasdeki, B. A. Pratt, M. Newsome, and S. Germann, "Taking mHealth Solutions to Scale: Enabling Environments and Successful Implementation," *J. Mob. Technol. Med.*, vol. 4, no. 1, pp. 35–38, Jan. 2015
[16] E.-H. Wang, L. Zhou, S.-H. K. Chen, K. Hill, and B. Parmanto, "An mHealth Platform for Supporting Clinical Data Integration into Augmentative and Alternative Communication Service Delivery: User-Centered Design and Usability Evaluation," *JMIR Rehabil. Assist. Technol.*, vol. 5, no. 2, p. e14, Jul. 2018
[17] J. Quinn, "An HL7 (Health Level Seven) Overview," *J. AHIMA*, vol. 70, no. 7, pp. 32–4, 1999
[18] M. L. Braunstein, "Patient - Physician Collaboration on FHIR (Fast Healthcare Interoperability Resources)," in *International Conference on Collaboration Technologies and Systems (CTS)*, pp. 501–503, 2015
[19] G. Kopanitsa, C. Hildebrand, J. Stausberg, and K. H. Englmeier, "Visualization of Medical Data Based on EHR Standards," *Methods Inf. Med.*, vol. 52, no. 1, pp. 43–50, Jan. 2013
[20] V. L. West, D. Borland, and W. E. Hammond, "Innovative Information Visualization of Electronic Health Record Data: A Systematic Review," *J. Am. Med. Informatics Assoc.*, Oct. 2014
[21] D. F. Sittig, D. R. Murphy, M. W. Smith, E. Russo, A. Wright, and H. Singh, "Graphical Display of Diagnostic Test Results in Electronic Health Records: a Comparison of 8 Systems," *J. Am. Med. Informatics Assoc.*, vol. 22, no. 4, pp. 900–904, Jul. 2015
[22] C. E. Kuziemsky, D. G. Schwartz, S. Airan-Javia, and R. Koppel, "Context and Meaning in EHR Displays," *Stud. Health Technol. Inform.*, vol. 265, pp. 69–73, Aug. 2019
[23] R. S. H. Istepanian and T. Al-Anzi, "m-Health 2.0: New Perspectives on Mobile Health, Machine Learning and Big Data Analytics," *Methods*, vol. 151, pp. 34–40, Dec. 2018
[24] S. Madanian, D. T. Parry, D. Airehrour, and M. Cherrington, "mHealth and Big-Data Integration: Promises for Healthcare System in India," *BMJ Heal. Care Informatics*, vol. 26, no. 1, p. e100071, Sep. 2019
[25] W. Nilsen *et al.*, "Advancing the Science of mHealth," *J. Health Commun.*, vol. 17, no. sup1, pp. 5–10, May 2012
[26] K. Naseer Qureshi, S. Din, G. Jeon, and F. Piccialli, "An Accurate and Dynamic Predictive Model for a Smart M-Health System Using Machine Learning," *Inf. Sci.*, vol. 538, pp. 486–502, Oct. 2020
[27] G. S. Collins and K. G. M. Moons, "Reporting of Artificial Intelligence Prediction Models," *Lancet*, vol. 393, no. 10181, pp. 1577–1579, Apr. 2019
[28] A. Coravos, S. Khozin, and K. D. Mandl, "Developing and Adopting Safe and Effective Digital Biomarkers to Improve Patient Outcomes.," *NPJ Digit. Med.*, vol. 2, no. 1, 2019

Platform	ePRO Support	Data Capture Modalities	Supported Data Types	EHR Integration Capabilities	Data Visualization Capabilities	Data Analysis Capabilities	COVID-19 Specific Solutions
Adaptive Clinical (Cherry Hill, NJ)	3rd party	Cloud to Cloud<br>Via Vendor's App/Hub	Manual Input of Data<br>Periodic/Aperiodic Test Results<br>Time-series Data	Download Data from EHR<br>Integrate EHR Data into Analysis<br>Send/Receive Alerts<br>Upload data to EHR	Not provided	Little to none	no
Artemis (Salt Lake City, UK)	Not supported	Cloud to Cloud	Periodic/Aperiodic Test Results<br>Time-series Data	Upload data to EHR	Integrated into EHR	Full Multivariate Time-series	no
Care Evolution (Ann Arbor, MI)	Included<br>3rd party	Cloud to Cloud<br>Via Vendor's App/Hub	Display interface from App<br>Manual Input of Data<br>Periodic/Aperiodic Test Results<br>Time-series Data	Download Data from EHR<br>Integrate EHR Data into Analysis<br>Send/Receive Alerts<br>Upload data to EHR	Web-based portal<br>Integrated into EHR	Full Multivariate Time-series	yes
ERT Clinical (Philadelphia, PA)	Included<br>3rd party	Direct from Sensor<br>Via Vendor's App/Hub	Manual Input of Data<br>Periodic/Aperiodic Test Results<br>Time-series Data	Download Data from EHR<br>Upload data to EHR	Web-based portal<br>Integrated into EHR	Little to none	no
Intersystems (Cambridge, MA)	Included<br>3rd party	Cloud to Cloud*<br>Direct from Sensor*<br>Via Vendor's App/Hub*<br>*Via Validic	Alarms from Sensors/Apps<br>Display interface from App<br>Manual Input of Data<br>Periodic/Aperiodic Test Results<br>Time-series Data	Download Data from EHR<br>Integrate EHR Data into Analysis<br>Send/Receive Alerts<br>Upload data to EHR	Web-based portal<br>Integrated into EHR	Full Multivariate Time-series	yes
Medable (Palo Alto, CA)	Included<br>3rd party	Cloud to Cloud<br>Direct from Sensor<br>Via Vendor's App/Hub	Alarms from Sensors/Apps<br>Display interface from App<br>Manual Input of Data<br>Periodic/Aperiodic Test Results<br>Time-series Data	Download Data from EHR<br>Integrate EHR Data into Analysis<br>Send/Receive Alerts<br>Upload data to EHR	Web-based portal	Full Multivariate Time-series	yes
PhysIQ (Chicago, IL)	Included	Cloud to Cloud<br>Direct from Sensor<br>Via Vendor's App/Hub	Manual Input of Data<br>Periodic/Aperiodic Test Results<br>Time-series Data	Send/Receive Alerts<br>Upload data to EHR	Web-based portal	Full Multivariate Time-series	yes
Redox (Madison, WI)	3rd party	Via Vendor's App/Hub	Manual Input of Data<br>Periodic/Aperiodic Test Results<br>Time-series Data	Download Data from EHR<br>Integrate EHR Data into Analysis<br>Send/Receive Alerts<br>Upload data to EHR	Not provided	Little to none	yes
TrialStat (Ottawa, Canada)	Included<br>3rd party	Cloud to Cloud<br>Via Vendor's App/Hub	Alarms from Sensors/Apps<br>Display interface from App<br>Manual Input of Data<br>Periodic/Aperiodic Test Results<br>Time-series Data	Download Data from EHR<br>Upload data to EHR	Web-based portal	Full Multivariate Time-series	no
Validic (Durham, NC)	3rd party	Cloud to Cloud<br>Direct from Sensor<br>Via Vendor's App/Hub	Alarms from Sensors/Apps<br>Display interface from App<br>Manual Input of Data<br>Periodic/Aperiodic Test Results<br>Time-series Data	Download Data from EHR<br>Send/Receive Alerts<br>Upload data to EHR	Integrated into EHR	Simple - Single variable	yes
Xealth (Seattle, WA)	3rd party	Cloud to Cloud	Display interface from App	Download Data from EHR<br>Send/Receive Alerts	Integrated into EHR	Little to none	yes

Table S8.2. Summary of the key capabilities of the considered mHealth data integration platforms

Summary Comments Including Issues and Opportunities

9.1 Introduction

The previous Sections of the Supplementary Materials have identified potential mHealth technologies by setting and use case. This compendium has highlighted the importance of appropriately matching and integrating such technologies to the environment and demands to which they are intended to be deployed.

The needs, complexity, and difficulties of diagnosing, treating, and tracing the individuals who have been exposed to and afflicted with COVID-19, are, like the virus itself, still evolving; with new information being made available every day. Harnessing the power and potential of mHealth technologies might aid in making our collective response to this and future crises less reactive and more proactive.

The different Sections of this report, as drafted by a multi-disciplinary team of experts across institutions and around the world, have attempted to address the core questions at the root of utilizing mHealth technologies in the context of the COVID-19 pandemic described in Section 1 of the Supplementary Materials. In the following, we provide a short section-by-section summary of the main conclusions drawn from the material presented in Sections 2-8.

9.2 mHealth in Disaster Scenarios

Field hospitals and alternative healthcare facilities are playing a vital role during the COVID-19 pandemic, relieving the pressure on healthcare systems. They serve as an additional triage layer accommodating patients with mild to moderate symptoms and isolating healthy individuals that are particularly at risk. In this scenario, wearable mHealth technologies can enhance field hospitals' patient care by providing continuous high-quality vital sign data and information. The authors, building from the Chinese experience of the Wuhan shelter identified three main areas of application for mHealth: 1) wearable sensors for vital sign monitoring; 2) portable imaging devices for advanced diagnostics; and 3) tracking systems for tracing staff and patients inside the facility. The fact that the optimal monitoring technology must be tailored to the specific use case was taken into consideration for the solutions presented for each of these categories. Additional pilot studies will be necessary to validate the use of these devices in the temporary, quickly constructed facilities that constitute most field hospitals.

9.3 ePRO: Electronic Patient-Reported Outcomes

The implementation of electronic Patient-Reported Outcomes (ePRO) solutions during the COVID-19 pandemic provides the opportunity for remote patient identification, classification, monitoring and management. The flexibility of ePRO to customize and update clinical algorithms based on the latest medical guidelines facilitates the proper identification and monitoring of populations which have a high probability of requiring medical attention. Geolocalization capabilities offer an opportunity to develop contact tracing programs and to visualize the surge of potential cases in specific areas. The modularity of these platforms enables their integration with multiple services and technologies such as telehealth, as well as Bluetooth-connected and other wireless wearable devices for real-time patient monitoring. These features, in conjunction with data collection and analysis, have the potential to empower healthcare systems and decision-makers with invaluable information to execute early interventions, making these platforms ideal to contain and mitigate pandemics such as COVID-19.

9.4 Monitoring Health Status of Patients and Healthcare Workers

In this Section, the authors discussed how mHealth technologies can be deployed for remote monitoring of individuals using solutions currently available on the market. Important attributes considered in evaluating these systems included: 1) the capability to measure relevant biosignals that may reflect the clinical status of patients affected by COVID-19; 2) technical/clinical validation of the measurable parameters; 3) ease of use; and 4) integration flexibility into existing hospital systems. To that end, a total of 27 candidate solutions were surveyed, of which ten example technologies met the functional criteria. The technologies that met the review criteria showed promise for monitoring pre-symptomatic healthcare workers during COVID-19's incubation and early prodromal stages, and for monitoring the physiological states of diagnosed patients in real-world settings.

9.5 Emerging mHealth Technologies

The COVID-19 pandemic has had a massive and sometimes overwhelming impact on healthcare systems around the world. There is an urgent need for innovative technologies, complementing established mobile and digital health systems, to cover the wide range of clinical needs emerging from this pandemic. In this Section, examples of these emerging technologies have been structured into five approaches: 1) contactless vital sign sensing; 2) monitoring of vital signs using wearable sensors; 3) audio- and spirometry-based characterization of lung function; 4) quantifying mental health via text-based analysis; and 5) robotic systems for patient management. Some of these technologies have already been successfully integrated into COVID-19 management strategies. But there are still open challenges that must be addressed before these systems can see wide adoption, from privacy concerns to data integration and, for some emergent technology, technical

maturation. Once these general issues have been hammered out, the full potential of mHealth and digital health technologies for managing the COVID-19 pandemic can hopefully be fulfilled.

9.6 TRACING PEOPLE IN THE HOSPITAL SETTING

Contact tracing is an established and effective means of monitoring and controlling the spread of infection. However, the process can be cumbersome and potentially unsustainable in the face of a pandemic on the scale of COVID-19. Technologies that enable indoor location tracking could be deployed alongside contact tracing software to streamline and automate the process within a hospital setting. The authors utilized publicly available information; contacted healthcare institutions that have already implemented indoor location systems; and spoke with engineers and clinicians familiar with internal tracing systems to determine requirements and identify candidate solutions. Several viable healthcare-specific location-based tracking systems with contact tracing capabilities were identified and surveyed. The effectiveness of such systems is highly dependent on system configuration and integration into hospital workflows. Currently, no existing implementations fully satisfy the needs for contact tracing of the current or future pandemics. The implementation of such systems should carefully consider specific use cases and institutional factors (e.g. infrastructure) during the initial design phase to ensure that the institution's unique needs and circumstances are met.

9.7 TRACING PEOPLE IN THE COMMUNITY

Proliferation of smartphones in the population has enabled the development of solutions that can aid manual contact tracing efforts, an invaluable boon to an essential aspect of managing and containing the spread of highly contagious diseases like COVID-19. Utilizing the technology inherent to smart devices and other such solutions can provide valuable information for rapid and accurate contact tracing. However, these solutions do capture sensitive data (e.g. proximity, location, phone number) and therefore present a noted risk to user privacy. The authors performed a detailed assessment of more than 40 solutions that fell into three categories: 1) smartphone apps, 2) online surveys, and 3) data aggregators. These solutions were assessed for characteristics such as: type of data captured, privacy policy, location tracing requirement, development status and geographical availability. The authors found significant differences between solutions and identified several limitations that can be addressed to improve the sensitivity and specificity of the information that can be used for community contact tracing.

9.8 MHEALTH DATA INTEGRATION PLATFORMS

To realize the benefits of mHealth technologies, it will be necessary to fully integrate the information from mobile health devices into the clinical decision-making process. Many platforms are being rapidly developed to try to address this challenge. And there are many factors that must be considered when selecting a platform, including: the ability to capture the right type of data; ability to analyze the data appropriately; and present the results to healthcare workers in a timely and actionable fashion. The time required to implement the platform into existing clinical systems and the ability of those system to manage this information are also critical to successfully adopting any mHealth technology. The authors reviewed eleven popular data integration platforms and provided summary information on these key issues.

9.9 A WORD OF CAUTION

Time is a huge factor when presenting the findings contained in the above Sections. Every day, new information about the virus is coming to light; and technologies are being developed and refined to meet an ever-evolving demand. As more information is made available, it will be necessary to reexamine the information presented herein, reconsider the entire enterprise, or redouble our efforts to see the wide adoption of mHealth technologies.

As it stands, there are clear trends towards early recognition of the virus including the detection of subtle symptoms [1]. For example, a unique feature of the disease is asymptomatic hypoxemia, which may be an early indicator of infection [2]. Even before the patient feels short of breath, it has been noted that they may experience desaturation which could be easily identified and monitored through an oximeter inside a healthcare facility, as well as in the home setting [2].

From the current vantage point, the conclusions contained in this report highlight the need to have means in place to monitor the population at-large and recognize the spread of the infectious disease; the need to monitor the well-being of healthcare workers; the need to adjust how the clinicians interact with their patients; and the need to recognize the rapidity of progression of COVID-19. Aspects that might be addressed through the wider adoption of mHealth technologies.

9.10 MHEALTH, WHAT IS NEXT?

When considering the recommendations outlined in this report, one must take into account what their respective healthcare system already has implemented as part of their clinical routine and any new technology should be able to synchronize with what is already deployed.

As more knowledge regarding the spread of this disease is obtained and the needs of patients (from the intensive care unit through discharge and home care) are made ever more evident, it is clear that the sequelae are significant [3], [4]. Complications from COVID-19 span from neurologic and cardiac affections to extreme deconditioning and loss of motor planning. The road to recovery for those afflicted will be long and more complicated than originally assumed [5]–[9]. A wide range of impairments (physical, cognitive, ...) requiring rehabilitation are expected post-COVID-19 infection. Healthcare providers will play an important role in following patients across different settings to maximize their functional return. It is anticipated that mHealth will become increasingly used to care for patients post-discharge, including for rehabilitation needs, through virtual visits for example [10].

These proposed uses of mHealth technologies have truly revolutionary potential. The decision-making ability of healthcare workers is significantly enhanced by the power of this added information when integrated into existing patient care algorithms. However, there is a sizable monetary cost to

the wide adoption of mHealth technology, as well as the fact that technology will eventually get outpaced by new advances in the industry and therefore would need to be replaced. Information technology, health literacy, and language barriers also present hurdles to mHealth implementation.

Some of the largest issues, as continually expressed in nearly every Section of this report, in the adoption of mHealth technologies are related to preserving privacy, establishing data sharing, maintaining accessibility and ensuring data security and safety. And while the technology rises to meet this challenge, regulations and policies will need to be enacted to ensure their safe use and smooth implementation into routine clinical care.

During this pandemic, hospitals around the world have implemented telehealth solutions often leveraging mHealth technologies to decrease the risk of exposure and contamination between healthcare workers and patients. For instance, tablets in patients' rooms allow nurses to communicate and check on patients, leading to a significant reduction in the use of personal protective equipment [11]. It should also be noted that this report did not include an in-depth update on the current uses of telehealth, which will prove to be equally as important and essential to the overall care of patients, from pre-hospital care to post-care. In fact, many healthcare systems have seen a drastic increase in telehealth visits to meet demand.

Through this current COVID-19 pandemic and into whatever the return to normalcy will be like, mHealth technologies will likely play a critical role. The effectiveness of mHealth will rely on large-scale adoption of technology integrated in clinical practice and overcoming the issues well-noted throughout this report. Yet preparedness is key. Be it for the next pandemic or other natural disaster, the effort made to prepare by leveraging the technical advancements of recent decades will repay the cost many times over in lives saved. This pandemic has accelerated the process towards implementation of wide-ranging modern medical technologies, mHealth included. The need for future preparedness will not abate.

References

[1] X. Li *et al.*, "Digital Health: Tracking Physiomes and Activity Using Wearable Biosensors Reveals Useful Health-Related Information," *PLOS Biol.*, vol. 15, no. 1, p. e2001402, Jan. 2017

[2] R. Levitan, "The Infection That's Silently Killing Coronavirus Patients," *The New York Times*, New York, NY, 20-Apr-2020

[3] F. J. Freyer, "Surviving Ventilators, Only to Find Lives Diminished," *Boston Globe*, Boston, 25-Apr-2020

[4] B. B. Kamdar *et al.*, "Joblessness and Lost Earnings After Acute Respiratory Distress Syndrome in a 1-Year National Multicenter Study," *Am. J. Respir. Crit. Care Med.*, vol. 196, no. 8, pp. 1012–1020, Oct. 2017

[5] H. Siddique, "Learning to Breathe Again: The Long Road to Recovery from Covid-19," *The Guardian*, 07-Apr-2020

[6] L. Brugliera *et al.*, "Rehabilitation of COVID-19 Patients," *J. Rehabil. Med.*, vol. 52, no. 4, p. jrm00046, 2020

[7] H. J. Stam, G. Stucki, and J. Bickenbach, "Covid-19 and Post Intensive Care Syndrome: A Call for Action," *J. Rehabil. Med.*, vol. 52, no. 4, p. jrm00044, Apr. 2020

[8] J. Helms *et al.*, "Neurologic Features in Severe SARS-CoV-2 Infection," *N. Engl. J. Med.*, vol. 382, no. 23, pp. 2268-2270, Apr. 2020

[9] T. Guo *et al.*, "Cardiovascular Implications of Fatal Outcomes of Patients With Coronavirus Disease 2019 (COVID-19)," *JAMA Cardiol.*, e201017, Mar. 2020

[10] R. Simpson and L. Robinson, "Rehabilitation Following Critical Illness in People with COVID-19 Infection," *Am. J. Phys. Med. Rehabil.*, 2020 [ePUB]

[11] P. Martineau, "iPads Are Crucial Health Care Tools in Combating Covid-19," *Wired*, 2020. [Online]. Available: https://www.wired.com/story/ipads-crucial-health-tools-combating-covid-19/. [Accessed: 28-June-2020]

AFFILIATIONS

Catherine P. Adans-Dester
Dept of Physical Medicine and Rehabilitation
Harvard Medical School at Spaulding Rehabilitation Hospital
Boston MA, USA

Stacy Bamberg
Veristride, Inc
Salt Lake City, UT

Francesco P Bertacchi
Wyss Institute for Biologically Inspired Engineering
Harvard University
Boston MA, USA

Brian Caulfield
University College Dublin
Dublin, Ireland

Kara Chappie
Individual contributor

Danilo Demarchi
Department of Electronics and Telecommunications
Politecnico di Torino
Turin, Italy

M Kelley Erb
Individual contributor

Juan Estrada
Center for TeleHealth
Massachusetts General Hospital
Boston MA, USA

Eric E Fabara
Dept of Physical Medicine and Rehabilitation
Harvard Medical School at Spaulding Rehabilitation Hospital
Boston MA, USA

Michael Freni
Mass General Brigham, Innovation, Business Development
Boston MA, USA

Karl E Friedl
U.S. Army Research Institute of Environmental Medicine (USARIEM)
Natick MA, USA
Department of Neurology
University of California, San Francisco
San Francisco CA, USA

Roozbeh Ghaffari
Epicore Biosystems, Inc
Cambridge MA, USA
Department of Biomedical Engineering
Northwestern University
Querrey-Simpson Institute for Bioelectronics
Evanston IL, USA

Geoffrey Gill
Shimmer Research, Inc
Cambridge MA, USA

Mark S Greenberg
Department of Psychiatry
Massachusetts General Hospital
Harvard Medical School
Boston MA, USA

Reed W Hoyt
U.S. Army Research Institute of Environmental Medicine (USARIEM)
Natick MA, USA

Emil Jovanov
Electrical and Computer Engineering Department
University of Alabama in Huntsville
Huntsville AL, USA

Christoph Kanzler
Rehabilitation Engineering Laboratory
Institute of Robotics and Intelligent Systems
Department of Health Sciences and Technology
ETH Zurich
Zurich, Switzerland

Dina Katabi
Computer Science and Artificial Intelligence Lab
Massachusetts Institute of Technology
Cambridge MA, USA

Meredith Kernan
Mass General Brigham, Innovation, Business Development
Boston MA, USA

Colleen Kigin
Department of Medicine and Rehabilitation
University of Colorado School of Medicine
Aurora CO, USA

Sunghoon I Lee
College of Information and Computer Sciences
University of Massachusetts
Amherst MA, USA

Steffen Leonhardt
Chair for Medical Information Technology
Helmholtz-Institute for Biomedical Engineering
RWTH Aachen University
Aachen, Germany

Nigel H Lovell
Graduate School of Biomedical Engineering
The University of New South Wales
Sydney NSW, Australia

Jose Mantilla
Motus Science
Melbourne, Australia

Thomas H. McCoy, Jr.
Center for Innovation in Digital HealthCare
Massachusetts General Hospital
Boston MA, USA

Nell Meosky Luo
Folia Health, Inc
Cambridge MA, USA

Glenn A Miller
Mass General Brigham
Boston MA, USA

John Moore
Fitbit
San Francisco CA, USA

Derek O'Keeffe
Galway University Hospitals
National University of Ireland
Galway, Ireland

Jeffrey Palmer
Human Health & Performance Systems
MIT Lincoln Laboratory
Lexington MA, USA

Federico Parisi
Dept of Physical Medicine and Rehabilitation
Harvard Medical School at Spaulding Rehabilitation Hospital
Boston MA, USA
Wyss Institute for Biologically Inspired Engineering
Harvard University
Boston MA, USA

Shyamal Patel
Pfizer Digital Medicine and Translational Imaging
Cambridge MA, USA

Jack Po
Alphabet Inc
Mountain View, CA

Benito L Pugliese
Dept of Physical Medicine and Rehabilitation
Harvard Medical School at Spaulding Rehabilitation Hospital
Boston MA, USA

Thomas Quatieri
Human Health & Performance Systems
MIT Lincoln Laboratory
Lexington MA, USA
Harvard-MIT Speech and Hearing Bioscience and Technology Program
Harvard Medical Sciences
Boston MA, USA

Tauhidur Rahman
College of Information and Computer Sciences
University of Massachusetts Amherst
Amherst MA, USA

Nathan Ramasarma
ArcSecond, Inc (dba formsense)
San Diego CA, USA

John A Rogers
Departments of Materials Science and Engineering, Biomedical Engineering and Neurological Surgery
Northwestern University
Querrey-Simpson Institute for Bioelectronics
Evanston IL, USA

Guillermo U Ruiz-Esparza
Division of Engineering in Medicine and Division of Renal Medicine
Brigham and Women's Hospital
Boston MA, USA
Division of Health Sciences and Technology
Harvard University - Massachusetts Institute of Technology,
Boston MA, USA

Stefano Sapienza
Dept of Physical Medicine and Rehabilitation
Harvard Medical School at Spaulding Rehabilitation Hospital
Boston MA, USA

Gregory Schiurring
Dept of Physical Medicine and Rehabilitation
Harvard Medical School at Spaulding Rehabilitation Hospital
Boston MA, USA

Lee Schwamm
Dept of Neurology and Center for TeleHealth
Massachusetts General Hospital
Harvard Medical School
Boston MA, USA
Virtual Care
Mass General Brigham
Somerville MA, USA

Hadi Shafiee
Division of Engineering in Medicine
Department of Medicine
Brigham and Womens' Hospital
Harvard Medical School
Cambridge MA, USA

Sara Kelly Silacci
Massachusetts General Hospital
Boston MA, USA
Mass General Brigham Center for COVID Innovation
Boston MA, USA

Nathaniel M Sims
Massachusetts General Hospital
Department of Anesthesia, Critical Care, and Pain Medicine
Associate Professor of Anesthesiology, Harvard Medical School
Ronald S. Newbower, PhD and Eitan Family Endowed Chair in Biomedical Technology Innovation, Massachusetts General Hospital
Boston MA, USA

Tanya Talkar
Speech and Hearing Bioscience and Technology
Harvard Medical School
Boston MA, USA
Human Health and Performance Systems
MIT Lincoln Laboratory
Lexington MA, USA

William J Tharion
U.S. Army Research Institute of Environmental Medicine (USARIEM)
Natick MA, USA

James A Toombs
Brigham Research Institute
Brigham and Women's Hospital
Boston MA, USA

Christopher Uschnig
Broad Institute of MIT and Harvard
Cambridge MA, USA
Department of Immunology and Infectious Diseases
Harvard T. H. Chan School of Public Health
Boston MA, USA

Gloria P. Vergara-Diaz
Dept of Physical Medicine and Rehabilitation
Harvard Medical School at Spaulding Rehabilitation Hospital
Boston MA, USA

Paul Wacnik
Digital Medicine and Translational Imaging
Pfizer Early Clinical Development
Cambridge MA, USA

May Wang
The Wallace H. Coulter Department of Biomedical Engineering
Georgia Institute of Technology and Emory University
Atlanta GA, USA

James Welch
Arc Biomedical Consulting
Mission Viejo CA, USA

Lina Williamson
TMAcceletator company LLC (TMA Precision Health)
Boston MA, USA

Ross Zafonte
Dept of Physical Medicine and Rehabilitation
Harvard Medical School at Spaulding Rehabilitation Hospital
Boston MA, USA

Adrian Zai
eCARE, Mass General Brigham
Boston MA, USA

Yuan-Ting Zhang
Department of Biomedical Engineering
City University of Hong Kong and Hong Kong Centre for Cerebro-cardiovascular Health Engineering (COCHE)
Hong Kong SAR, China

Guillermo J Tearney
Massachusetts General Hospital
Boston MA, USA

Rushdy Ahmad
Wyss Institute for Biologically Inspired Engineering
Harvard University Center for Life Science Boston
Boston MA, USA

David R. Walt
Brigham and Women's Hospital
Harvard Medical School
Wyss Institute
Harvard University
Boston MA, USA

Paolo Bonato
Dept of Physical Medicine and Rehabilitation
Harvard Medical School at Spaulding Rehabilitation Hospital
Boston MA, USA
Wyss Institute for Biologically Inspired Engineering
Harvard University
Boston MA, USA

DISCLOSURES

Catherine P. Adans-Dester
No disclosures.

Stacy Bamberg
SB is Founder & CEO of Veristride, Inc., which is in the field of gait analysis using proprietary wearable technology and analysis, specifically for neurorehabilitation patients, including Stroke survivors, persons with Parkinson's Disease, persons with Cerebral Palsy, etc. Veristride has been collaborating with Harvard Medical School/Spaulding Rehabilitation Hospital and University of Maryland for the past several years, supported in part by funding from the National Science Foundation and the National Institutes of Health (NIH).

Francesco P Bertacchi
No disclosures.

Brian Caulfield
No disclosures.

Kara Chappie
KC was a contractor to Pfizer and is a Pfizer shareholder.

Danilo Demarchi
No disclosures.

M. Kelley Erb
MKE is a paid employee of Biogen, Inc.

Juan Estrada
No disclosures.

Eric E. Fabara
No disclosures.

Michael Freni
No disclosures.

Karl E. Friedl
KF has previously been involved in Army funding decisions for biotechnology development and applications research and is compensated only as a government employee.

Roozbeh Ghaffari
RG has received grant support from the NextFlex Manufacturing Institute, the National Institutes of Health (NIH), the Bill and Melinda Gates Foundation, and the Department of Defense. He has active collaborations with Leo Pharma Science and Tech Hub, PepsiCo, L'Oreal, 3M, and US Air Force Research Laboratory. He is co-founder of Epicore Biosystems and MC10, and serves in a scientific role and advisor for Rhaeos, Sibel Health, and Neurolux.

Geoffrey Gill
GG is the President of Shimmer Research Inc, a manufacturer of wearable sensors for research and medical applications.

Mark S. Greenberg
MSG holds a minor equity stake in Personal Health Insights (PHI), Inc., a start-up company involved in designing platforms for the electronic data capture of health-related information.

Reed W. Hoyt
No disclosures.

Emil Jovanov
No disclosures.

Christoph Kanzler
No disclosures.

Dina Katabi
No disclosures.

Meredith Kernan
No disclosures.

Colleen Kigin
CK is the Chair of the Physical Therapy Program Scholarship and Endowment Board, and Visiting Clinical Professor, Physical Therapy Program, Department of Physical Medicine and Rehabilitation,, University of Colorado, and Member Governance Committee, Easter Seals of Massachusetts.

Sunghoon I. Lee
SIL has received grant support from the National Science Foundation (NSF), the National Institutes of Health (NIH), and he Armstrong Fund for Research.

Steffen Leonhardt
No disclosures.

Nigel H. Lovell
No disclosures.

Jose Mantilla
JM is founder of Motus Science, a technology company that leverages advances in artificial intelligence and smartphone sensors to understand movement and behavioral patterns at individual and aggregated levels.

Thomas H. McCoy, Jr.
TM receives research funding from the Stanley Center at the Broad Institute, the Brain and Behavior Research Foundation, National Institute of Mental Health, National Human Genome Research Institute Home, and Telefonica Alfa.

Nell Meosky Luo
NML is the Founder and CEO of Folia Health Inc. The company develops ePRO products.

Glenn A. Miller
No disclosures.

John Moore
JM serves as Medical Director at Fitbit.

Derek O'Keeffe
No disclosures.

Jeffrey Palmer
DISTRIBUTION STATEMENT A. Approved for public release. Distribution is unlimited.
This material is based upon work supported by the Under Secretary of Defense for Research and Engineering under Air Force Contract No. FA8702-15-D-0001. Any opinions, findings, conclusions or recommendations expressed in this material are those of the author(s) and do not necessarily reflect the views of the Under Secretary of Defense for

Research and Engineering.
© 2020 Massachusetts Institute of Technology.
Delivered to the U.S. Government with Unlimited Rights, as defined in DFARS Part 252.227-7013 or 7014 (Feb 2014). Notwithstanding any copyright notice, U.S. Government rights in this work are defined by DFARS 252.227-7013 or DFARS 252.227-7014 as detailed above. Use of this work other than as specifically authorized by the U.S. Government may violate any copyrights that exist in this work.

Federico Parisi
No disclosures.

Shyamal Patel
SP is an Employee and shareholder of Pfizer Inc.

Jack Po
JP is an employee and a shareholder of Alphabet Inc.

Benito L. Pugliese
No disclosures.

Thomas Quatieri
DISTRIBUTION STATEMENT A. Approved for public release. Distribution is unlimited.
This material is based upon work supported by the Under Secretary of Defense for Research and Engineering under Air Force Contract No. FA8702-15-D-0001. Any opinions, findings, conclusions or recommendations expressed in this material are those of the author(s) and do not necessarily reflect the views of the Under Secretary of Defense for Research and Engineering.
© 2020 Massachusetts Institute of Technology.
Delivered to the U.S. Government with Unlimited Rights, as defined in DFARS Part 252.227-7013 or 7014 (Feb 2014). Notwithstanding any copyright notice, U.S. Government rights in this work are defined by DFARS 252.227-7013 or DFARS 252.227-7014 as detailed above. Use of this work other than as specifically authorized by the U.S. Government may violate any copyrights that exist in this work.

Tauhidur Rahman
No disclosures.

Nathan Ramasarma
NR is the founder & CEO of ArcSecond, Inc. (dba formsense). ArcSecond, Inc. is a party to an expcted grant from the National Institutes of Health (NIH). Formsense has been collaborating with Harvard Medical School/Spaulding Rehabilitation Hospital, University of Massachusetts at Amherst for the past few years and recetntly in addition, with the University of Maryland-College Park in the field of stroke rehabilitation aided by formsense proprietary wearable technology.

John A. Rogers
JAR has received grant support from the the Department of Defense, the National Institutes of Health (NIH), the National Science Foundation (NSF), the Bill and Melinda Gates Foundation, Save the Children Foundation, and the Gerber Foundation. He serves on the Board of Directors for Epicore Biosystems, MC10, Sibel Health, Rhaeos, Sonaco, Wearifi, and Neurolux in the field of biointegrated medical systems and devices.

Guillermo U. Ruiz-Esparza
No disclosures.

Stefano Sapienza
No disclosures.

Gregory Schiurring
No disclosures.

Lee Schwamm
LHS is a full-time employee of Massachusetts General Hospital and Mass General Brigham and serves as a consultant on user interface design and usability to LifeImage (privately held teleradiology company).

Hadi Shafiee
HS has received funding from the National Institute of Health (NIH), Partners Halthcare, and Brigham and Women's Hospital Precision Medicine Program.

Sara Kelly Silacci
No disclosures.

Nathaniel M. Sims
No disclosures.

Tanya Talkar
DISTRIBUTION STATEMENT A. Approved for public release. Distribution is unlimited.
This material is based upon work supported by the Under Secretary of Defense for Research and Engineering under Air Force Contract No. FA8702-15-D-0001. Any opinions, findings, conclusions or recommendations expressed in this material are those of the author(s) and do not necessarily reflect the views of the Under Secretary of Defense for Research and Engineering.
© 2020 Massachusetts Institute of Technology.
Delivered to the U.S. Government with Unlimited Rights, as defined in DFARS Part 252.227-7013 or 7014 (Feb 2014). Notwithstanding any copyright notice, U.S. Government rights in this work are defined by DFARS 252.227-7013 or DFARS 252.227-7014 as detailed above. Use of this work other than as specifically authorized by the U.S. Government may violate any copyrights that exist in this work.
Supported by NIH-NIDCD T32 DC000038.

William J. Tharion
WJT serves as a U.S. Army's Contracting Officer Representative (COR) on U.S. Army contracts awarded to Human Systems Integration (Walpole, MA) and Cornerstone Research Group (Miamisburg, OH) and formerly served as a COR on a contract awarded to Equivital Ltd. (Cambridge, UK). In these roles he provides advice to these contractors to meet U.S. Army requirements. He is compensated by the U.S. Army for his roles but not by the companies.

James A. Toombs
No disclosures.

Christopher Uschnig
No disclosures.

Gloria P. Vergara-Diaz
GVD has received fellowship support from the Alfonso Martin Escudero Foundation (Spain) and from the Real

Colegio Complutense at Harvard. She is the recipient of a grant from the Foundation for Physical Medicine & Rehabilitation (PM&R) and serves as an investigator on a National Institutes of Health (NIH) SBIR grant awarded to Veristride (Salt Lake City UT).

Paul Wacnik
PW is an Employee and shareholder of Pfizer Inc

May Wang
MDW received funding from CDC, Enduring Heart, Amazon Research, Georgia Tech Petit Institute Faculty Fellow Fund, Carol Ann and David Flanagan Faculty Fellow Fund, Children's Healthcare of Atlanta, Shriners Hospitals for Children, and NIH.

James Welch
JW provides regulatory and design consulting services to emerging medical device companies.

Lina Williamson
No disclosures.

Ross Zafonte
RZ has received royalties from 1) Oakstone for an educational CD-Physical Medicine and Rehabilitation a Comprehensive Review; 2) Demos publishing for serving as co-editor of the text Brain Injury Medicine. Dr. Zafonte serves on the Scientific Advisory Board of Myomo, Oxeia Biopharma, and ElMINDA. He also evaluates patients in the MGH Brain and Body-TRUST Program which is funded by the NFL Players Association.

Adrian Zai
No disclosures.

Yuan-Ting Zhang
No disclosures.

Guillermo J. Tearney
No disclosures.

Rushdy Ahmad
No disclosures.

David R. Walt
DRW receives grant support from the Michael J Fox Foundation, the National Institutes of Health (NIH), Gates Foundation, Open Philanthropy, Chan-Zuckerberg Foundation. He is a Director, equity holder, and/or receives payments from Quanterix, Exicure, Arbor Biotechnologies, Ultivue, Sherlock Biosciences, and Vizgen.

Paolo Bonato
PB has received grant support from the American Heart Association, the Department of Defense, the Michael J Fox Foundation, the National Institutes of Health (NIH), the National Science Foundation (NSF), and the Peabody Foundation including sub-awards on NIH and NSF SBIR grants from Barrett Technology (Newton MA), BioSensics (Watertown MA), and Veristride (Salt Lake City UT). He has also received grant support from Emerge Diagnostics (Carlsbad CA), MC10 (Lexington MA), Mitsui Chemicals (Tokyo Japan), Pfizer (New York City NY), Shimmer Research (Dublin Ireland), and SynPhNe (Singapore). He serves in an advisory role the Michael J Fox Foundation, the NIH-funded Center for Translation of Rehabilitation Engineering Advances and Technology, and the NIH-funded New England Pediatric Device Consortium. He also serves on the Scientific Advisory Boards of Hocoma AG (Zurich Switzerland), Trexo (Toronto Canada), and ABLE Human Motion (Barcelona, Spain) in an uncompensated role.
